# Measuring and Monte Carlo Modelling of X-Ray and Gamma-Ray Attenuation in Personal Radiation Shielding Protective Clothing

**DOI:** 10.1155/2019/1641895

**Published:** 2019-11-19

**Authors:** Michaela Kozlovska, Jaroslav Solc, Petr Otahal

**Affiliations:** ^1^Nuclear Protection Department, National Institute for NBC Protection (SUJCHBO v.v.i.), Kamenna 262 31, Pribram, Czech Republic; ^2^Czech Metrology Institute, Brno 638 00, Czech Republic

## Abstract

A collection of personal protective equipment (PPE), suitable for use in case of accident in nuclear facilities or radiological emergencies, was gathered at the National Institute for Nuclear, Chemical and Biological Protection, Czech Republic. The shielding characteristics of the various PPE materials were measured via narrow geometry spectral attenuation measurements with point radionuclide sources covering a broad range of photon energies. Photon relative penetration and attenuation for relevant energies of the spectra were the principal experimentally determined quantities for tested PPE. Monte Carlo simulations in the MCNPX™ code were carried out to determine photon attenuation for respective energies in the tested PPE, and the results were compared to those determined experimentally. Energy depositions in a unit volume of an ORNL phantom were simulated in a radioactive aerosols atmospheric environment to determine effective doses both for the whole body and in various organs in the human torso during exposure to different dispersed radioactive aerosols while wearing one of the PPE protecting against X- and gamma-ray. This work aimed to determine the effective dose and its decrease for individual PPE protecting against X- and gamma-ray.

## 1. Introduction

Some samples of personal protective equipment (PPE), protecting against X- and gamma-ray, were collected and tested at the National Institute for NBC Protection (SUJCHBO v.v.i.), Czech Republic. This type of PPE can be used by first responders in emergencies such as an accident during radioactive material transportation, terrorist incidents involving radiological dispersal devices (RDD) or nuclear weapons, or by specialised emergency response workers during accidents in nuclear facilities.

The authors are not aware of any other published studies concerning X- and gamma-ray attenuation in PPE.

The collection of PPE protecting against X- and gamma-ray due to the presence of shielding layers comprising heavy metals or their alloys can be divided into two groups: (a) body overalls, covering the whole body and (b) local garments, primarily covering the torso and shielding radiation-sensitive body organs, together with nonworn equipment, such as a radiation protection shield. OPCH-90 PPE (without a shielding layer) was chosen as a reference PPE.  Table 1 presents individual tested PPE together with their parameters from the manufacturers' materials and experimentally determined density thicknesses.

Relative penetration and attenuation of X- and gamma-ray, penetrating the samples and originating from different radionuclide point sources, were determined for a broad energy range, together with mass attenuation coefficients, which describe the attenuating qualities of shielding layer materials in individual PPE. Corresponding lead equivalents of individual shielding layers, which express the thickness of the equally attenuating lead sheet, were calculated as well.

The experimentally determined values of these abovementioned quantities for the first part of the collection of PPE protecting against X- and gamma-ray were presented by Kozlovska [2].

Together with experimental testing, Monte Carlo (MC) simulations of selected individual PPE were carried out. The following quantities were simulated: relative penetration and attenuation of monoenergetic photons by individual shielding layers and energy deposition in a unit volume of the ORNL phantom while wearing or not-wearing an individual PPE protecting against X- and gamma-ray.

The X- and gamma-ray penetration rates in simulated shielding layer material of individual PPE were determined for the main energies of the spectra. The decrease of effective dose both in the whole body and, due to the presence of PPE, the human torso alone were also assessed by the MC modelling of a human body during exposure to selected dispersed radioactive aerosols.

## 2. Materials and Methods

### 2.1. Measurement of X- and Gamma-Ray Spectra

The spectra of X- and gamma-ray, penetrating tested PPE samples and originating from different radionuclide point sources, were measured by two handheld spectrometers: an InSpector 1000 with detachable IPROL-1 scintillation LaBr_3_ probe, and a Falcon 5000 HPGe detector, both manufactured by Canberra Industries, Inc., USA.

**Figure 1:**
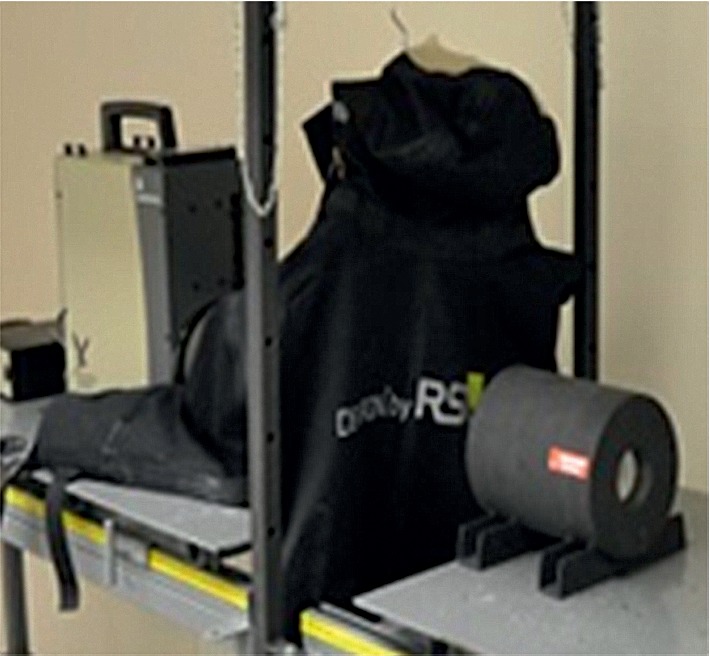
Spectra measurement arrangement.

Samples of tested PPE were placed on a holder between the source and the detector at the defined distance to meet the lowest possible dead time of the detector and avoid false pulse coincidence. Photon spectra were measured on a testing bench, which enables the precise coaxial narrow beam geometry settings of the source, the detector, and the set of both outer and inner cylindrical collimators (Figure 1). Outer collimators were made from lead, and their outer and inner diameters were 16.5 cm and 6.0 cm, respectively; the lengths were 22.5 cm and 40.5 cm, respectively, in the case of IPROL-1 LaBr_3_ probe measurement, which was inserted into the collimator. Inner collimators were also made of lead at lengths of 8.5 cm, and their outer and inner aperture diameters were 6.0 cm and 1.2 cm, respectively.

The narrow beam geometry setting was used for spectra measurement to minimise the number of scattered photons contributing to the measured spectra unwantedly influencing peak areas and causing a build-up effect.

To cover the wide photon energy range from 30.5 keV to 1,408 keV, the following radioactive point sources activation ranging from 9.5 to 40.0 MBq were used: ^241^Am, ^133^Ba, ^152^Eu, ^137^Cs, and ^60^Co.

Measurement live times ranged from 120 to 1,800 s to obtain net peak areas for all significant sufficiently high full-energy peaks, achieving their relative uncertainty below 1%.

Measured spectra were subsequently analysed using Genie 2000 Gamma Analysis Software v. 3.2, manufactured by Canberra Industries, Inc. For the primary spectra energies, corresponding to the main peaks of total absorption, photon penetration and attenuation rates in a tested PPE sample were determined. These quantities were determined via comparing net peak areas in the spectra, measured with the sample placed between the source and the detector, and the corresponding net peak areas in the spectra, measured without any samples, for the same time between the source and the detector.

The energy dependency of mass attenuation coefficients was determined from the relative attenuation and experimentally assessed density thicknesses of individual PPE samples to describe shielding qualities of individual shielding layer materials. For each tested sample, a lead equivalent, which sets the thickness of the equally attenuating lead sheet, was determined using the values of the lead mass attenuation coefficient, stated by Johnson and Birty [3]. Mean lead equivalents for individual PPE protecting against X- and gamma-ray was set as mean of lead equivalents determined for the individual energies of significant peaks, except for absorption edges.

Together with the spectra of photons penetrating tested PPE samples, the spectra of photons penetrating a lead sheet (thickness of 1.25 mm) were similarly measured, and the same quantities were determined to test the measurement and spectra evaluation methods.

### 2.2. Radon-Aerosol Chamber

The Radon-Aerosol Chamber (RAC) at SUJCHBO v.v.i. we used for PPE testing in a dispersed radioactive atmosphere is a gas-tight testing box of 10 m^3^ volume with dimensions of 250 cm (length) × 200 cm (width) × 200 cm (height), strictly separated from the ambient atmosphere. The RAC enables the creation of a stable atmosphere containing high concentrations of radioactive or nonradioactive aerosol particles under various physical conditions. It is also possible for the created aerosol particles to be sampled and measured externally. The RAC parameters are described by Burian [4].

### 2.3. Monte Carlo Simulations

Monte Carlo (MC) simulations were performed using the general-purpose MC code MCNPX™ version 2.7.E [5]. Full photon and electron transport and the detailed treatment of bremsstrahlung radiation were considered in the whole volume of the RAC. Photon transport utilised the MCPLIB04 photoatomic data library described by White [6], and the low-energy cutoff was set to 10 keV. Electron transport utilised the el03 library described by Adams [7], and the low-energy cutoff was set to 50 keV.

### 2.4. Simulations of PPE Materials

The elemental compositions of PPE materials necessary for input to the MC model were taken from the results of X-ray fluorescence spectrometry (XRF) and scanning electron microscopy (SEM) measurements. In addition, during the later analysis, energy-dispersive X-ray spectrometry was also applied to collect additional material information.

A simple MC model consisting of a point source emitting monoenergetic photons perpendicularly to a layer of PPE material was used to calculate relative penetration in individual PPE samples. Material relative penetration for each photon energy was obtained using an F1-type tally located behind the layer counting the number of photons (per one photon emitted from the source) that did not undergo interaction in the layer. The statistical uncertainty of the F1-tally results was 0.1%. Consequently, the relative attenuation and relative penetration at different energies were compared to those measured experimentally.

For input to the MC model, the mass density of the samples was determined by finding the best match between measured and calculated relative penetration, keeping the elemental composition fixed.

### 2.5. Simulations of Effective Dose

MC simulations of energy deposition in a unit volume of an ORNL phantom described by Eckerman et al. [8] were performed in the chosen dispersed radioactive aerosol environment in the RAC. The ORNL phantom was positioned in a standing position at the centre of the RAC, and it was modelled to either wear one of the simulated PPE protecting against X- and gamma-ray (respectively, the simulated PPE together with a PPE preventing radioactive contamination over it in the case of local PPE protecting against X- and gamma-ray), or to solely wear PPE preventing radioactive contamination.

The following dispersed radionuclides were simulated: ^99m^Tc, ^131^I, ^137^Cs, ^140^La, and ^24^Na. ^131^I and ^137^Cs radionuclides emit both beta particles and gamma-ray of medium energies, while ^140^La and ^24^Na radionuclides emit both beta particles and gamma ray of high energies. However, the ^99m^Tc radionuclide only emits gamma ray of low energy.

Both gamma and beta contributions to the effective dose were taken into account in the simulations. Energy distributions were taken from Radiological Toolbox [9], which contains data from the ICRP 38 publication [10]. Three radiation sources were considered in the RAC: (1) dispersion of the radionuclide in the air; (2) deposition on the RAC walls; and (3) deposition on the outer surface of the tested PPE or covering PPE preventing radioactive contamination. For the evaluation of the results, it was assumed that 90% of the total activity was dispersed in the air and that the surface activity of the RAC walls and PPE were equal.

The equivalent dose was determined by calculation of the absorbed dose in the ORNL phantom organs. The deposited energy in each organ was initially obtained using the ^*∗*^F8-type tally independently for each of three radiation sources and both gamma and beta contributions. The deposited energies in each organ were then recalculated into the equivalent dose per unit activity. The equivalent doses were finally weighted by the tissue weighting factors defined in the ICRP 103 publication [11] and summed into the effective dose.

The resulting calculated values of the whole body effective dose and equivalent doses on various organs were compared to the values of the same quantities, simulated in the same way for the ORNL phantom without the PPE. The effective dose on the human torso alone was also considered to compare individual whole-body PPE protecting against X- and gamma-ray and local ones. The following organs were included in the effective dose on the human torso calculation: lungs, stomach, colon, testes and genitalia, liver, oesophagus, urinary bladder, small intestine, gall bladder, pancreas, spleen, heart, adrenals, kidneys, and thymus.

The decrease of equivalent dose on various organs, as well as whole-body effective dose decrease and decrease of effective dose on the human torso for individual PPE protecting against X- and gamma-ray, used in atmospheres of various dispersed radionuclides, were subsequently calculated from the resulting values of corresponding equivalent and effective doses.

## 3. Results and Discussion

### 3.1. Measurement of X- and Gamma-Ray Spectra

**Figure 2:**
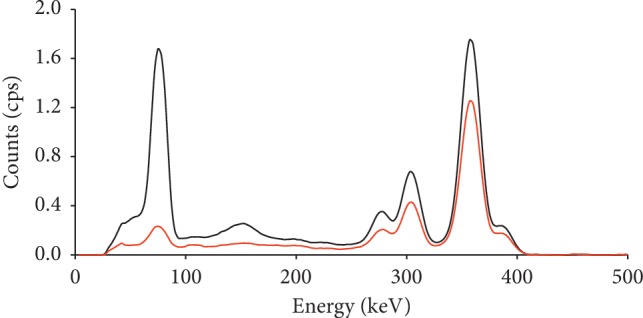
Smoothed spectra of radionuclide ^133^Ba gamma-ray without a PPE (black line) and penetrating BIORUBBER E-600 Vest (red line), measured with an InSpector 1000 spectrometer with a detachable scintillation LaBr_3_ probe IPROL-1.

Figure 2 presents the spectra of the ^133^Ba radionuclide gamma-ray without PPE and the penetrating BIORUBBER E-600 Vest PPE sample. The spectra were measured with an InSpector 1000 spectrometer with a detachable IPROL-1 scintillation LaBr_3_ probe and smoothed by averaging over three adjacent channels. A significant decrease of relative attenuation of more energetic photons, penetrating the sample, is evident from the spectra.

**Figure 3:**
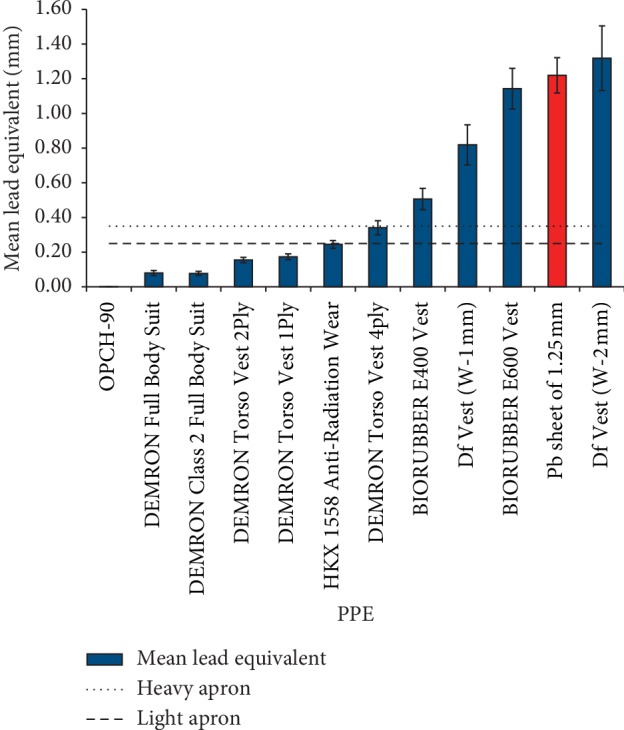
Mean lead equivalents of tested PPE (the mean lead equivalent of reference Pb sheet is red) together with CENELEC requirements for heavy and light aprons. Error bars represent the standard deviation of measurements.

Experimentally determined mean lead equivalents for individual PPE protecting against X- and gamma-ray, together with a reference PPE without a shielding layer and a reference lead sheet, are presented in Figure 3. The mean lead equivalent for the DEMRON IED RDD Shield was not determined due to a strong build-up factor in the thick material, inversely depending on penetrating photon energy.

From the mean lead equivalent values of tested PPE samples, it is evident that the higher the density thickness of a tested sample, the higher its lead equivalent (Figure 3 and Table 1). The mean lead equivalent of some of the tested PPE protecting against X- and gamma-ray does not even meet the requirements of 0.35 mm, respectively, 0.25 mm, for the lead equivalent of heavy protective aprons, respectively, light protective aprons, used by radiological operators, respectively, used in operating rooms, and specified by CENELEC 1999 [12].

The lead equivalent of the reference OPCH-90 PPE (without any shielding layer) is almost negligible; the mean lead equivalent of the reference Pb sheet of 1.22 ± 0.10 mm corresponds to its thickness of 1.25 mm. The experimentally assessed mean lead equivalent value for HKX 1558 Whole-Body Anti-Radiation Wear equal to 0.24 ± 0.02 mm corresponds to the value of 0.25 mm stated by the manufacturer (Table 1).

The experimentally determined lead equivalent for Df Vest W-1 mm equal to 0.99 ± 0.10 mm at the energy of 1,332.5 keV corresponds to the value of 1.0 mm stated by the manufacturer, while the same quantity for Df Vest W-2 mm of 1.71 ± 0.06 mm is 14.5% lower than the value of 2.0 mm stated by the manufacturer (Table 1). However, lead equivalent values determined at lower energies for both these PPE, as well as mean lead equivalents, are lower, which indicates the presence of a build-up factor in these relatively thick materials.

## 4. Monte Carlo Simulations of PPE Material

**Figure 4:**
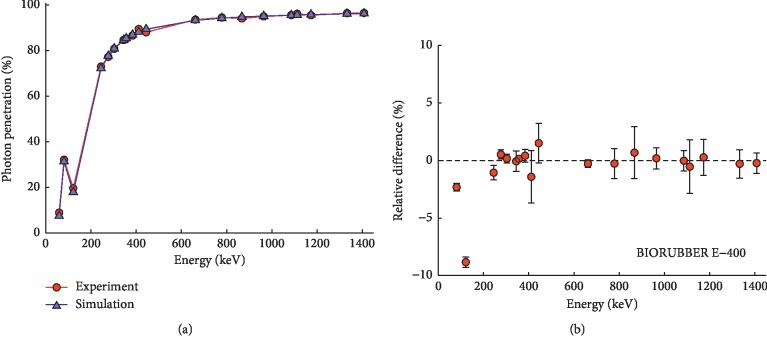
BIORUBBER E-400 Vest material. (a) Measured and simulated photon relative penetration. Error bars are equivalent to data point size. (b) Relative difference between measured and simulated relative photon penetration.

Figure 4 presents the energy dependence of measured and simulated photon relative penetration in the BIORUBBER E-400 Vest and their relative difference. Both measured and simulated penetration rates are in excellent agreement, which proves the correctness of (1) the PPE material composition determined by XRF and SEM measurements and (2) the estimated density thickness of the material. The decreased relative photon penetration (and therefore increased attenuation) at the energy of about 88 keV corresponds to the lead absorption edge, which occurs at photoelectric absorption when there is sufficient photon energy to eject an electron from the K-shell.

Similar agreement between measured and simulated attenuation and penetration was obtained for all other studied materials as well.

## 5. Monte Carlo Simulations of Effective Dose

**Figure 5:**
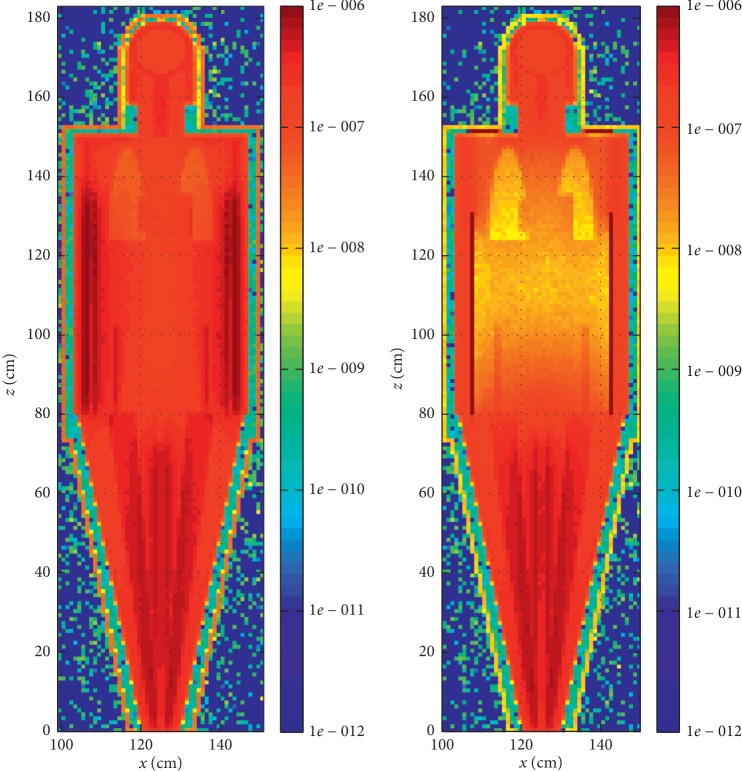
An example of simulated energy deposition in ORNL phantom, wearing PPE preventing radioactive contamination only (a) and the same PPE together with BIORUBBER E-600 Vest (b) in dispersed ^99m^Tc aerosol atmosphere. Colorbar in relative units.

Figure 5 presents an example of visualisation of simulated ORNL phantom energy depositions while only wearing PPE preventing radioactive contamination, and the same PPE together with the BIORUBBER E-600 Vest under it, in a dispersed ^99m^Tc aerosol atmosphere. Visualisations for other PPE or other radionuclides are presented in the electronic annex.

For the selected dispersed radioactive atmosphere, a decrease of energy deposition due to the presence of PPE protecting against X- and gamma-ray depends on the PPE lead equivalent. On the other hand, the higher the beta and gamma particle energy emitted by dispersed radionuclide, the lower the decrease of energy deposition, as higher energy particles are much less attenuated by PPE (Figure 2).

**Figure 6:**
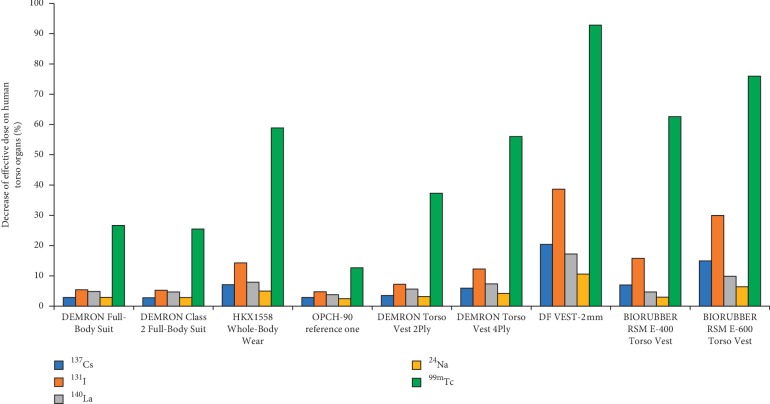
Simulated decrease of effective dose on human torso organs when protected with tested PPE exposed to various radionuclides dispersed in the atmosphere.

Table 2 presents decrease of the various organs' contribution to the effective dose when protected with individual tested PPE exposed to the ^131^I radionuclide dispersed in 10 m^3^ of the atmosphere in the RAC geometry. Numerical results for other studied radionuclides are presented in the electronic annex. However, the results for other radionuclides are graphically presented in Figure 6, which summarises values of the effective dose decrease on human torso organs when protected with individual tested PPE exposed to various radionuclides in the given geometry.

For individual simulated dispersed radionuclides, it is evident from Figure 6 that the higher the density thickness (and corresponding lead equivalent) of the PPE protecting against X- and gamma-ray, the higher the decrease of the effective dose. On the other hand, the higher the beta and gamma particle energy emitted by dispersed radionuclides, the lower the decrease of the effective dose.

For the simulated ^99m^Tc radionuclide, the decrease of the effective dose on the human torso organs of an ORNL phantom, protected by individual PPE protecting against X- and gamma-ray, is significantly higher than the resulting decrease when protected by the reference PPE OPCH-90 without a shielding layer. However, in the case of other simulated radionuclides, the decrease of the effective dose on human torso organs is only significantly higher than that of the reference PPE if the ORNL phantom is protected by PPE of a sufficient lead equivalent, which meets the CENELEC requirements for heavy and light aprons, used to shield against diagnostic medical X-ray radiation (Figures 6 and 3).

Most of the radiation-sensitive organs of the body are situated in the human torso, so the decrease of the whole-body effective dose obtained with vests is similar to the decrease of human torso effective dose only. For the same decrease of effective dose, it is more efficient to use vests instead of whole-body PPE because the vests are much lighter. For example, the Demron Radiation Torso Vest 4 Ply weighs 4.3 kg, while HKX 1558 Whole-Body Anti-Radiation Wear weighs 10.1 kg. However, the effective dose decrease on human torso organs gained with these two PPE is comparable.

## 6. Conclusions

A collection of personal protective equipment protecting against X- and gamma-ray was gathered and tested at the National Institute for NBC Protection, Czech Republic. Relative penetration of X- and gamma-ray in tested samples in the energy range from 30.5 to 1,408 keV was determined.

Measurements were supported with Monte Carlo simulations in the MCNPX™ code determining relative penetration and attenuation of PPE. The simulated data resulted in excellent agreement with measurements. After this validation of the MC models of tested PPE, simulations of energy deposition in organs of an ORNL phantom, in the chosen dispersed radioactive aerosol environment, were performed. Consequently, to estimate the efficiency of the PPE, whole-body effective dose and equivalent dose in various organs in the human torso were calculated and compared to the values of the same quantities, similarly simulated for the ORNL phantom without the simulated PPE in the various dispersed radioactive aerosol atmospheres.

## Figures and Tables

**Table 1:** Whole body and local PPE protecting against X- and gamma-ray.

	Whole-body PPE protecting against X- and gamma-ray				Local PPE protecting against X- and gamma-ray				
PPE	DEMRON Full Body Suit	DEMRON Class 2 Full Body Suit^*∗∗∗*^	HKX 1558 Whole Body Anti-Radiation Wear	OPCH-90 (the reference one)	DEMRON Radiation Torso Vest 1/2/4 Ply	Df Vest W-2mm/W-1mm	BIORUBBER E-400 Vest + Pants	BIORUBBER E-600 Vest	DEMRON IED RDD Shield^*∗∗∗∗∗*^
Picture	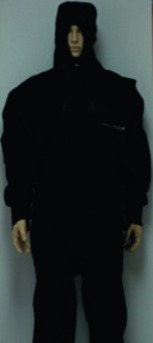	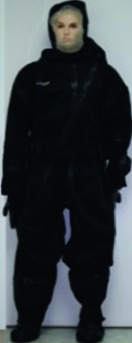	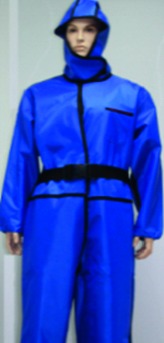	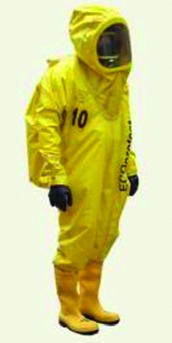	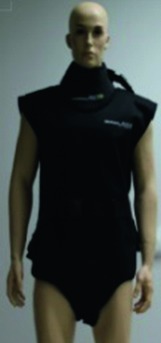	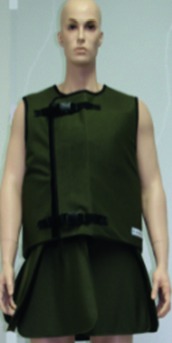	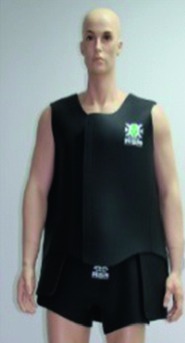	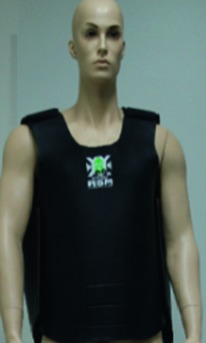	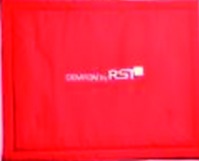
Producer	Radiation Shield Technology, USA	Radiation Shield Technology, USA	Guangzhou Hekang Biotechnology co. Ltd., China	Ecoprotect s.r.o., Czech Republic	Radiation Shield Technology, USA	Alpha Technical Research co. Ltd., Japan	YAMAMOTO Corporation, Japan	YAMAMOTO Corporation, Japan	Radiation Shield Technology, USA
Shielding layer material	DEMRON^*∗*^	DEMRON^*∗*^	Lead compounds, dispersed in rubber	—	DEMRON^*∗*^ 1/2/4 ply	Tungsten, dispersed in resin	BIORUBBER E-400^*∗∗*^	BIORUBBER E-600^*∗∗*^	DEMRON^*∗*^
Shielding properties	Not given	Not given	Lead equivalent 0.25 mm	—	Not given	Lead equivalent 2.0 mm/1.0 mm^*∗∗∗∗*^	^137^Cs dose rate decrease 4.4%	^137^Cs dose rate decrease 10.5%	Not given
Surface layer	Nonwoven textile	Nonwoven textile	Textile	Butyl rubber	Nonwoven textile	Nylon	Antiadhesive BRS	Antiadhesive BRS	Nonwoven textile
Density thickness (g·cm^−2^)	0.13	0.13	0.40	0.053	0.32/0.26/0.47	2.08/1.12	0.72	1.61	3.97

^*∗*^DEMRON is a water- and gas-tight polymer composite of PE, PVC, and inorganic salts of high-atomic number elements DEMRON Full Body Suit (except for lead). ^*∗∗*^BIORUBBER consists of heavy metal (primarily lead) alloys dispersed in synthetic material with a regular honeycomb structure of cells, created by pure limestone. ^*∗∗∗*^Meets the NFPA 1994/2007 requirements, presented by the National Fire Protection Association [1]. ^*∗∗∗∗*^Lead equivalent set for energy 1332.5 keV of radionuclide^60^Co. ^*∗∗∗∗∗*^Protects against improvised explosive devices (IEDs) and radiological dispersive devices (RDDs).

**Table 2:** Decrease of various organs' contribution to the effective dose when protected with individual tested PPE in an atmosphere of dispersed radionuclide^131^I. Organs included in “torso organs” are listed in the text.

PPE protecting against X- and gamma-ray	Torso organs (%)	Lungs (%)	Testes and genitalia (%)	Brain (%)	Thyroid (%)	Skin (%)
DEMRON Full Body Suit	−5.4	−4.9	−4.8	−1.5	−1.0	−84.8
DEMRON Class 2 Full Body Suit	−5.2	−4.7	−4.5	−1.5	−0.9	−84.8
HKX 1558 Whole Body Wear	−14.3	−13.5	−15.1	−8.0	−11.3	−88.0
OPCH 90 (the reference one)	−4.7	−4.3	−0.5	1.6	3.9	−82.1
DEMRON Torso Vest 2Ply	−7.2	−6.3	−8.5	−1.0	−4.3	−39.2
DEMRON Torso Vest 4Ply	−12.3	−11.0	−13.2	−1.6	−7.2	−39.6
DF Vest W-2 mm	−38.7	−34.0	−46.3	−3.9	−19.7	−50.8
BIORUBBER RSM E400 Torso Vest	−15.8	−16.2	−5.2	−1.9	−9.3	−36.1
BIORUBBER RSM E600 Torso Vest	−30.0	−30.9	−7.2	−3.3	−14.3	−35.9

## Data Availability

The measured spectra and simulated energy depositions in the ORNL phantom used to support the findings of this study are available from the corresponding author upon request. Visualisations of simulated energy depositions in the ORNL phantom are presented in the electronix annex.

## Supplementary Materials

Supplementary Materials The electronic annex presents (1) numerical decrease of the various organs' contribution to the effective dose when protected with individual tested PPE exposed to the other simulated radionuclides dispersed in 10 m3 of the atmosphere in the RAC geometry (Table 2) and (2) visualisations of simulated ORNL phantom energy depositions while only wearing PPE preventing radioactive contamination, and the same PPE together with individual PPE protecting against X- and gamma-ray under it, in a various-dispersed radionuclide's aerosol atmosphere.
