# Supplementary figures and images for: Measuring and Monte Carlo Modelling of X-Ray and Gamma-Ray Attenuation in Personal Radiation Shielding Protective Clothing (part 1 of 2)
Source: Comput Math Methods Med. 2019 Nov 19;2019:1641895. doi: 10.1155/2019/1641895 (PMC6885783; doi:10.1155/2019/1641895)

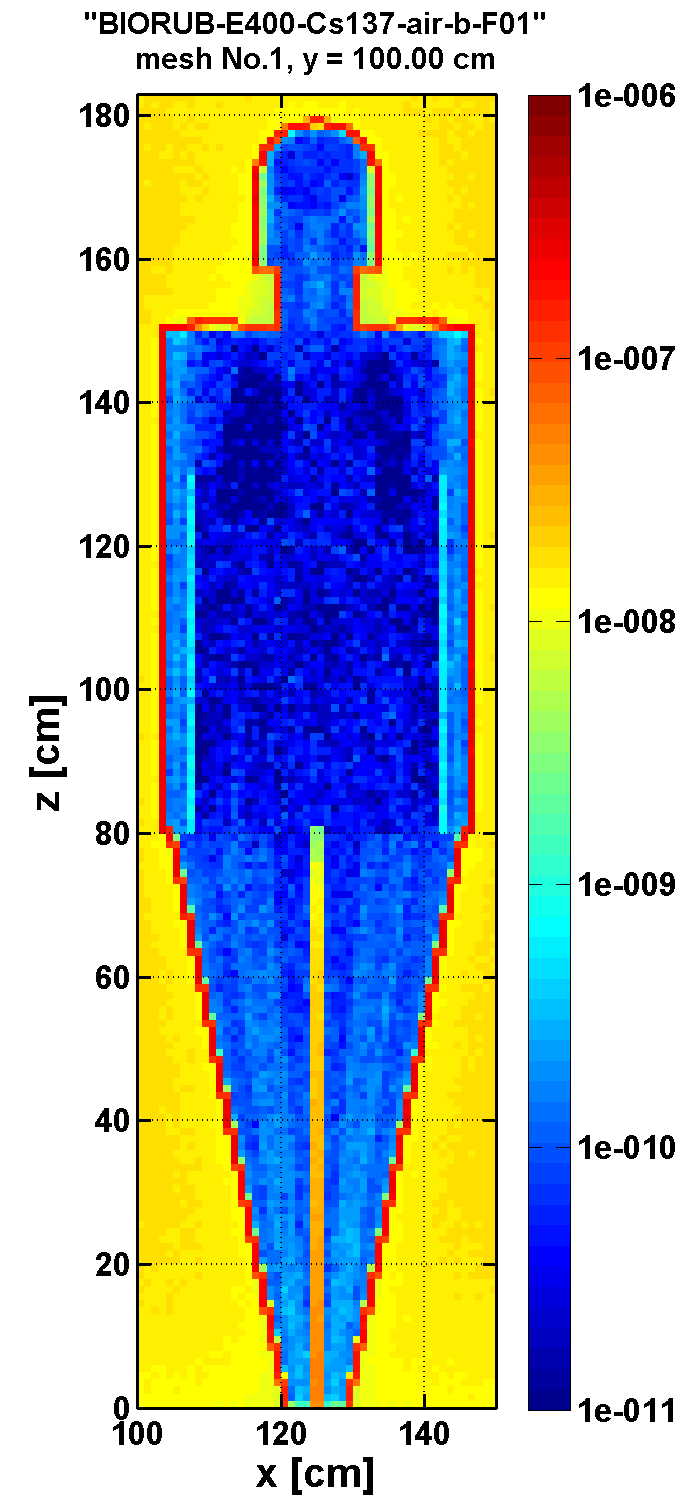

Supplement: Supplementary Materials — The electronic annex presents (1) numerical decrease of the various organs' contribution to the effective dose when protected with individual tested PPE exposed to the other simulated radionuclides dispersed in 10 m3 of the atmosphere in the RAC geometry (Table 2) and (2) visualisations of simulated ORNL phantom energy depositions while only wearing PPE preventing radioactive contamination, and the same PPE together with individual PPE protecting against X- and gamma-ray under it, in a various-dispersed radionuclide's aerosol atmosphere. [file 1641895.f1.zip › 1641895.f1/Electronic annex/Visualization of 2D distributions/Beta contribution/XZ/XZ-b-air-Cs137-BIORUB-E400.png]

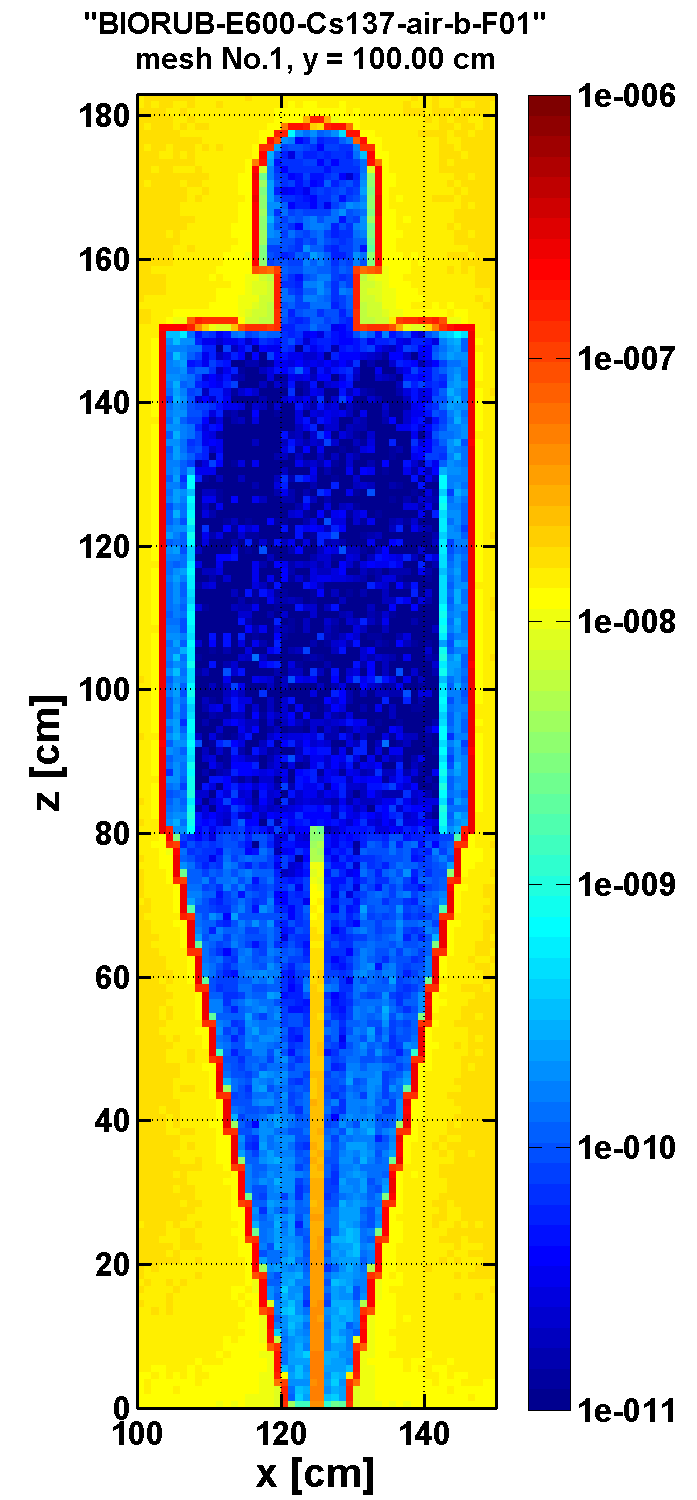

Supplement: Supplementary Materials — The electronic annex presents (1) numerical decrease of the various organs' contribution to the effective dose when protected with individual tested PPE exposed to the other simulated radionuclides dispersed in 10 m3 of the atmosphere in the RAC geometry (Table 2) and (2) visualisations of simulated ORNL phantom energy depositions while only wearing PPE preventing radioactive contamination, and the same PPE together with individual PPE protecting against X- and gamma-ray under it, in a various-dispersed radionuclide's aerosol atmosphere. [file 1641895.f1.zip › 1641895.f1/Electronic annex/Visualization of 2D distributions/Beta contribution/XZ/XZ-b-air-Cs137-BIORUB-E600.png]

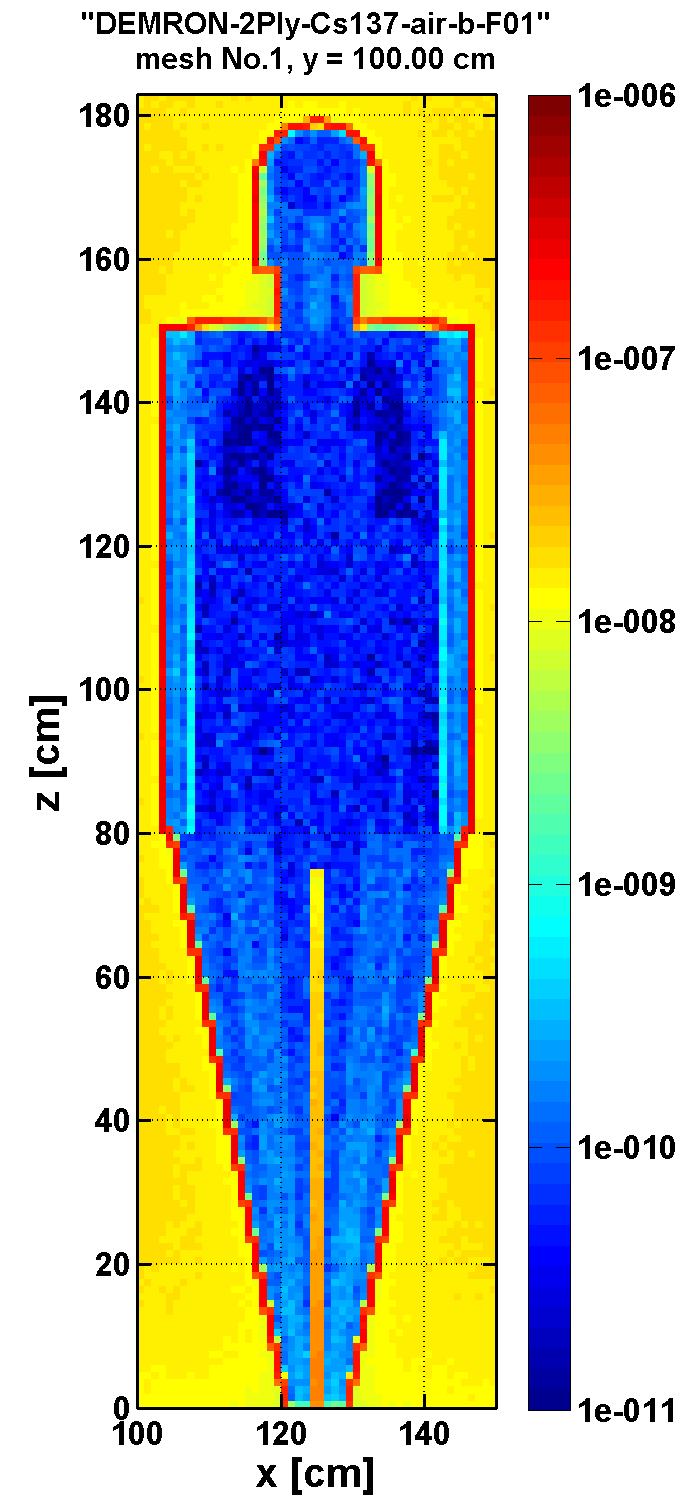

Supplement: Supplementary Materials — The electronic annex presents (1) numerical decrease of the various organs' contribution to the effective dose when protected with individual tested PPE exposed to the other simulated radionuclides dispersed in 10 m3 of the atmosphere in the RAC geometry (Table 2) and (2) visualisations of simulated ORNL phantom energy depositions while only wearing PPE preventing radioactive contamination, and the same PPE together with individual PPE protecting against X- and gamma-ray under it, in a various-dispersed radionuclide's aerosol atmosphere. [file 1641895.f1.zip › 1641895.f1/Electronic annex/Visualization of 2D distributions/Beta contribution/XZ/XZ-b-air-Cs137-DEMRON-2Ply.png]

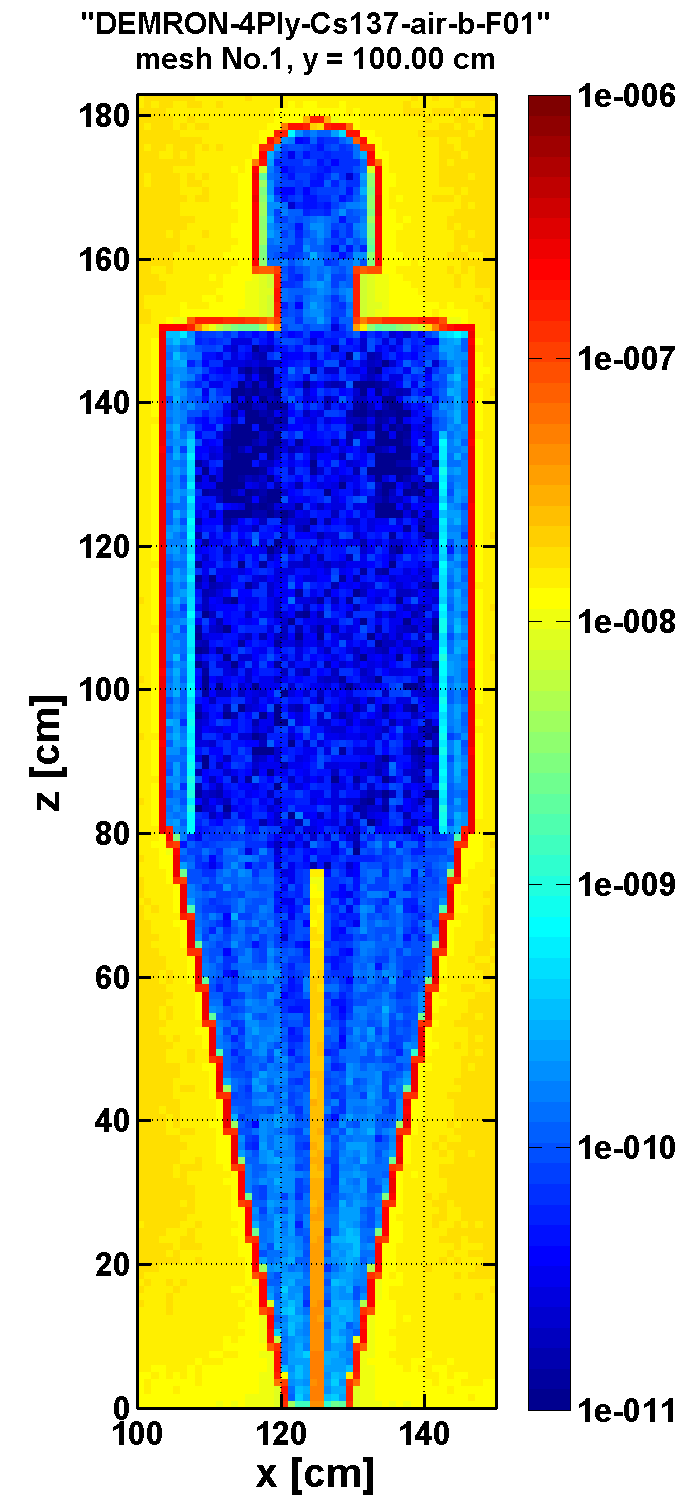

Supplement: Supplementary Materials — The electronic annex presents (1) numerical decrease of the various organs' contribution to the effective dose when protected with individual tested PPE exposed to the other simulated radionuclides dispersed in 10 m3 of the atmosphere in the RAC geometry (Table 2) and (2) visualisations of simulated ORNL phantom energy depositions while only wearing PPE preventing radioactive contamination, and the same PPE together with individual PPE protecting against X- and gamma-ray under it, in a various-dispersed radionuclide's aerosol atmosphere. [file 1641895.f1.zip › 1641895.f1/Electronic annex/Visualization of 2D distributions/Beta contribution/XZ/XZ-b-air-Cs137-DEMRON-4Ply.png]

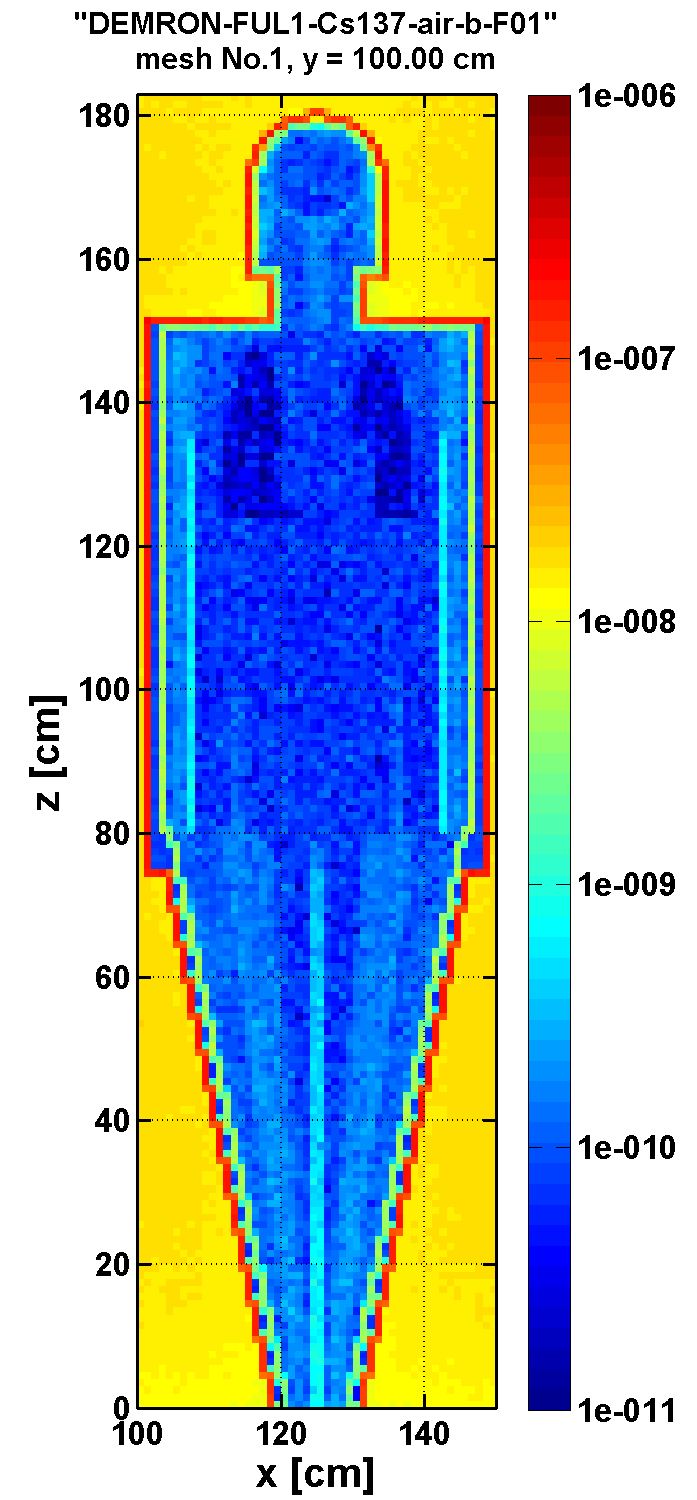

Supplement: Supplementary Materials — The electronic annex presents (1) numerical decrease of the various organs' contribution to the effective dose when protected with individual tested PPE exposed to the other simulated radionuclides dispersed in 10 m3 of the atmosphere in the RAC geometry (Table 2) and (2) visualisations of simulated ORNL phantom energy depositions while only wearing PPE preventing radioactive contamination, and the same PPE together with individual PPE protecting against X- and gamma-ray under it, in a various-dispersed radionuclide's aerosol atmosphere. [file 1641895.f1.zip › 1641895.f1/Electronic annex/Visualization of 2D distributions/Beta contribution/XZ/XZ-b-air-Cs137-DEMRON-FUL1.png]

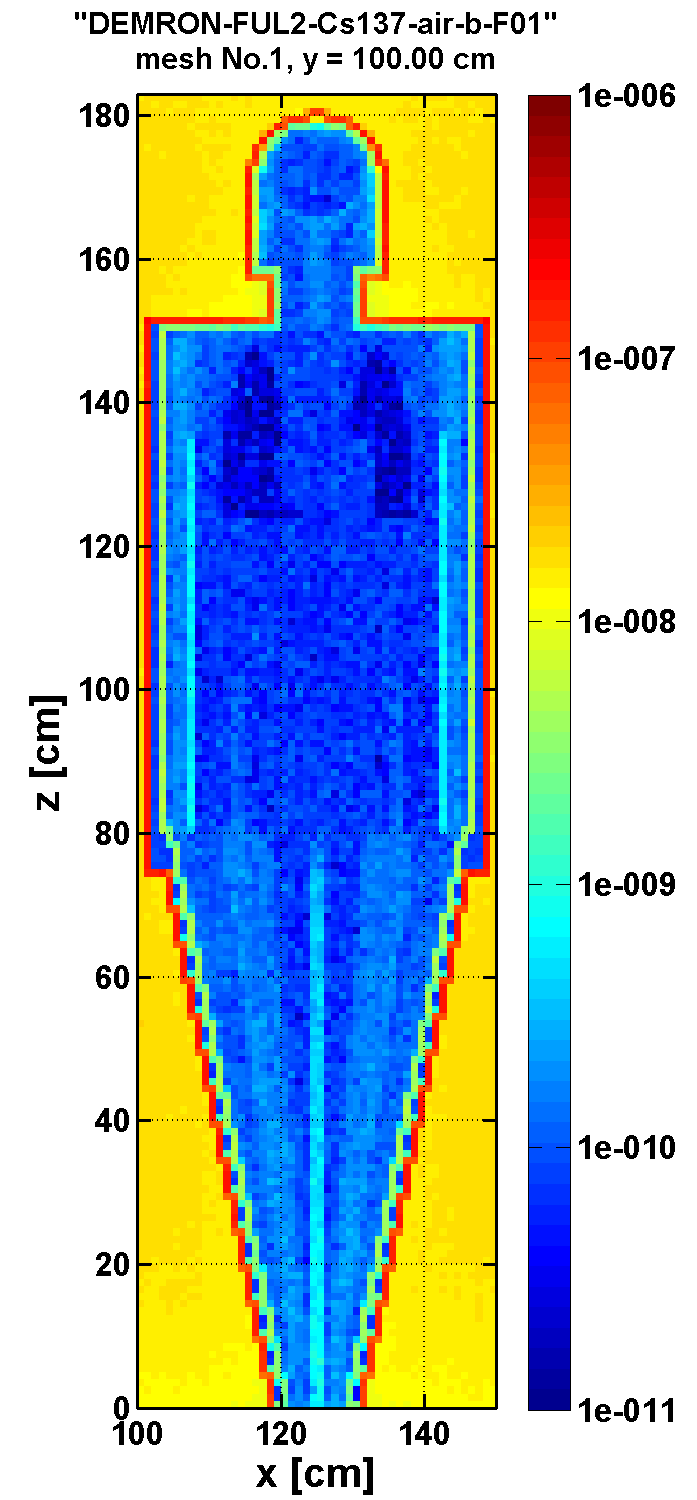

Supplement: Supplementary Materials — The electronic annex presents (1) numerical decrease of the various organs' contribution to the effective dose when protected with individual tested PPE exposed to the other simulated radionuclides dispersed in 10 m3 of the atmosphere in the RAC geometry (Table 2) and (2) visualisations of simulated ORNL phantom energy depositions while only wearing PPE preventing radioactive contamination, and the same PPE together with individual PPE protecting against X- and gamma-ray under it, in a various-dispersed radionuclide's aerosol atmosphere. [file 1641895.f1.zip › 1641895.f1/Electronic annex/Visualization of 2D distributions/Beta contribution/XZ/XZ-b-air-Cs137-DEMRON-FUL2.png]

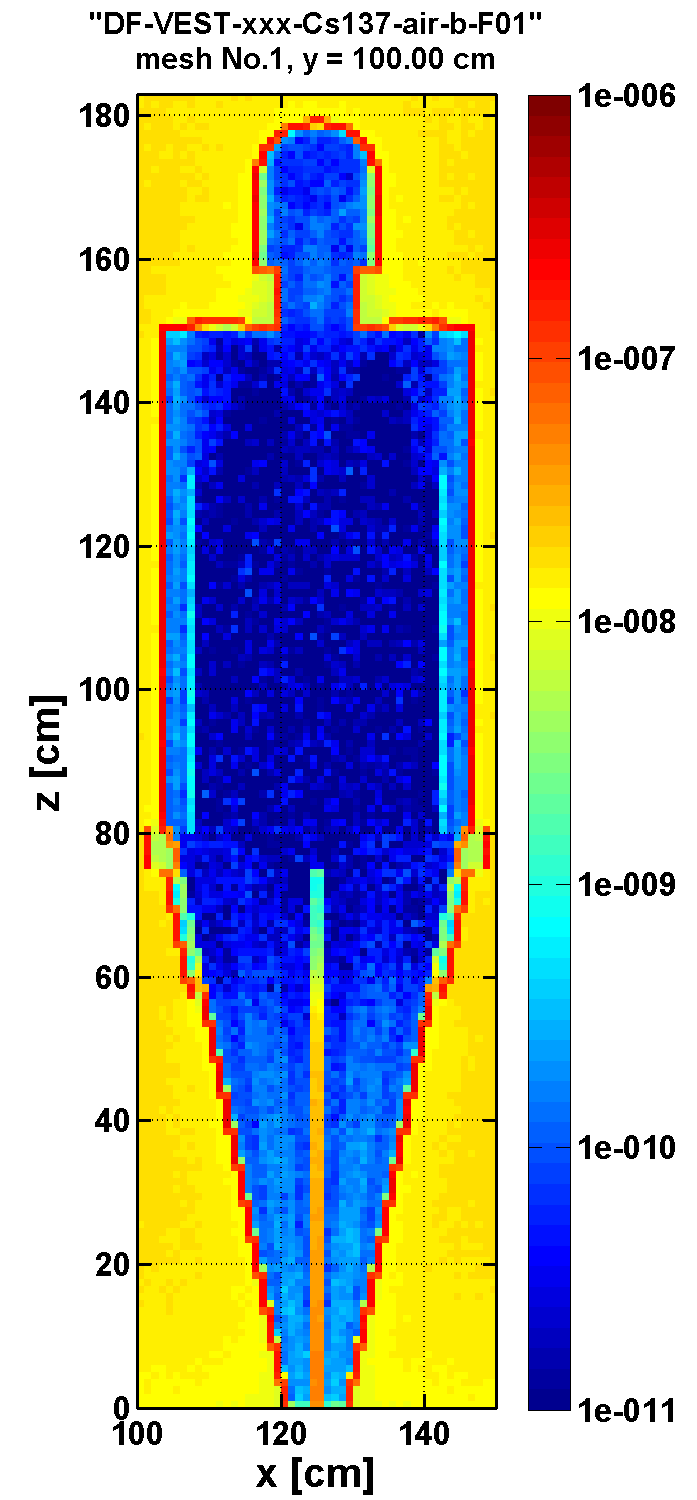

Supplement: Supplementary Materials — The electronic annex presents (1) numerical decrease of the various organs' contribution to the effective dose when protected with individual tested PPE exposed to the other simulated radionuclides dispersed in 10 m3 of the atmosphere in the RAC geometry (Table 2) and (2) visualisations of simulated ORNL phantom energy depositions while only wearing PPE preventing radioactive contamination, and the same PPE together with individual PPE protecting against X- and gamma-ray under it, in a various-dispersed radionuclide's aerosol atmosphere. [file 1641895.f1.zip › 1641895.f1/Electronic annex/Visualization of 2D distributions/Beta contribution/XZ/XZ-b-air-Cs137-DF-VEST.png]

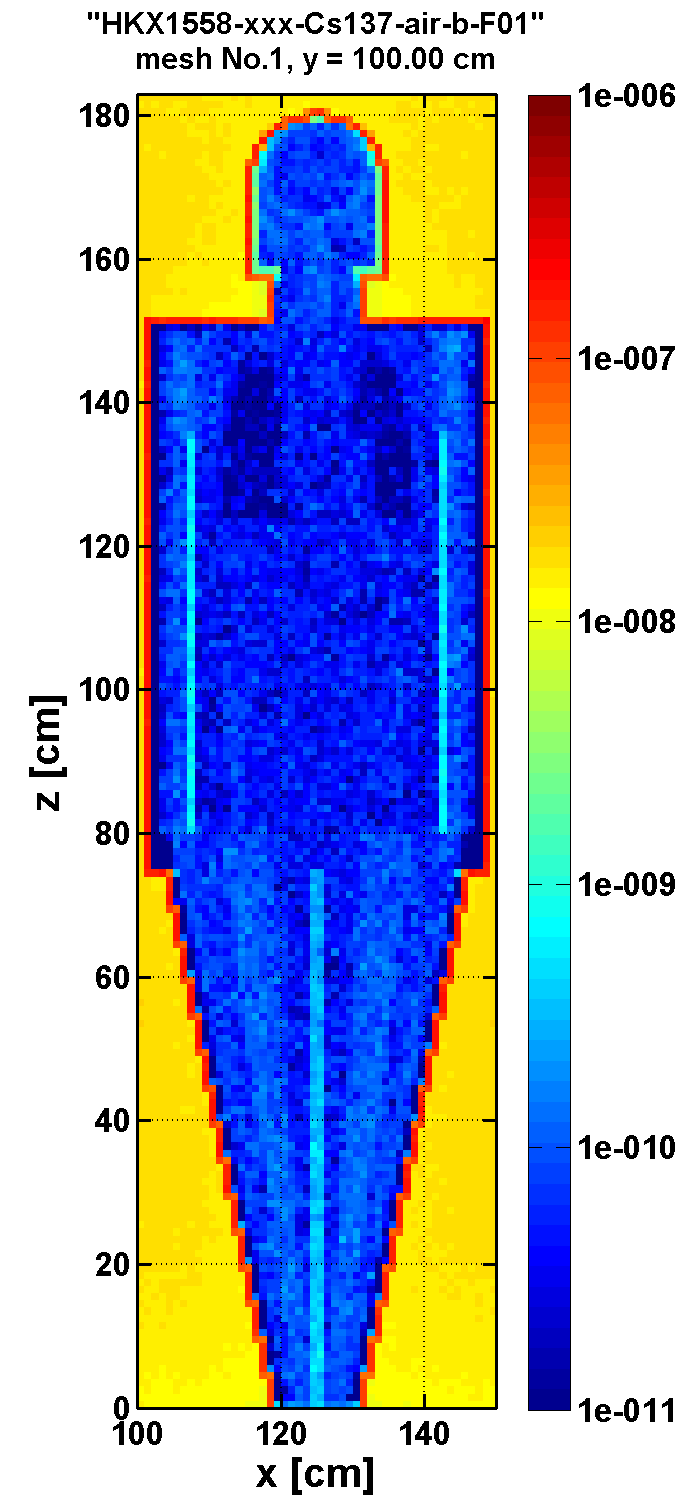

Supplement: Supplementary Materials — The electronic annex presents (1) numerical decrease of the various organs' contribution to the effective dose when protected with individual tested PPE exposed to the other simulated radionuclides dispersed in 10 m3 of the atmosphere in the RAC geometry (Table 2) and (2) visualisations of simulated ORNL phantom energy depositions while only wearing PPE preventing radioactive contamination, and the same PPE together with individual PPE protecting against X- and gamma-ray under it, in a various-dispersed radionuclide's aerosol atmosphere. [file 1641895.f1.zip › 1641895.f1/Electronic annex/Visualization of 2D distributions/Beta contribution/XZ/XZ-b-air-Cs137-HKX1558.png]

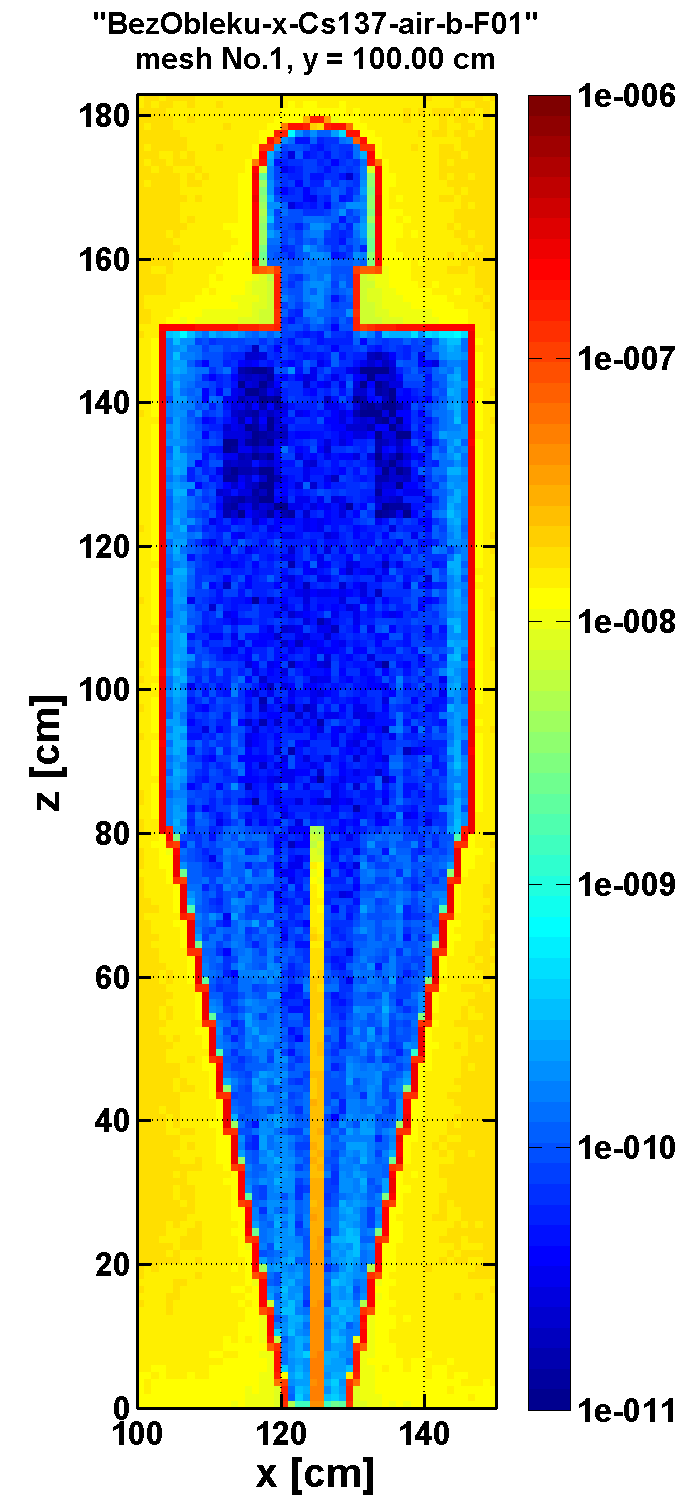

Supplement: Supplementary Materials — The electronic annex presents (1) numerical decrease of the various organs' contribution to the effective dose when protected with individual tested PPE exposed to the other simulated radionuclides dispersed in 10 m3 of the atmosphere in the RAC geometry (Table 2) and (2) visualisations of simulated ORNL phantom energy depositions while only wearing PPE preventing radioactive contamination, and the same PPE together with individual PPE protecting against X- and gamma-ray under it, in a various-dispersed radionuclide's aerosol atmosphere. [file 1641895.f1.zip › 1641895.f1/Electronic annex/Visualization of 2D distributions/Beta contribution/XZ/XZ-b-air-Cs137-NoPPE.png]

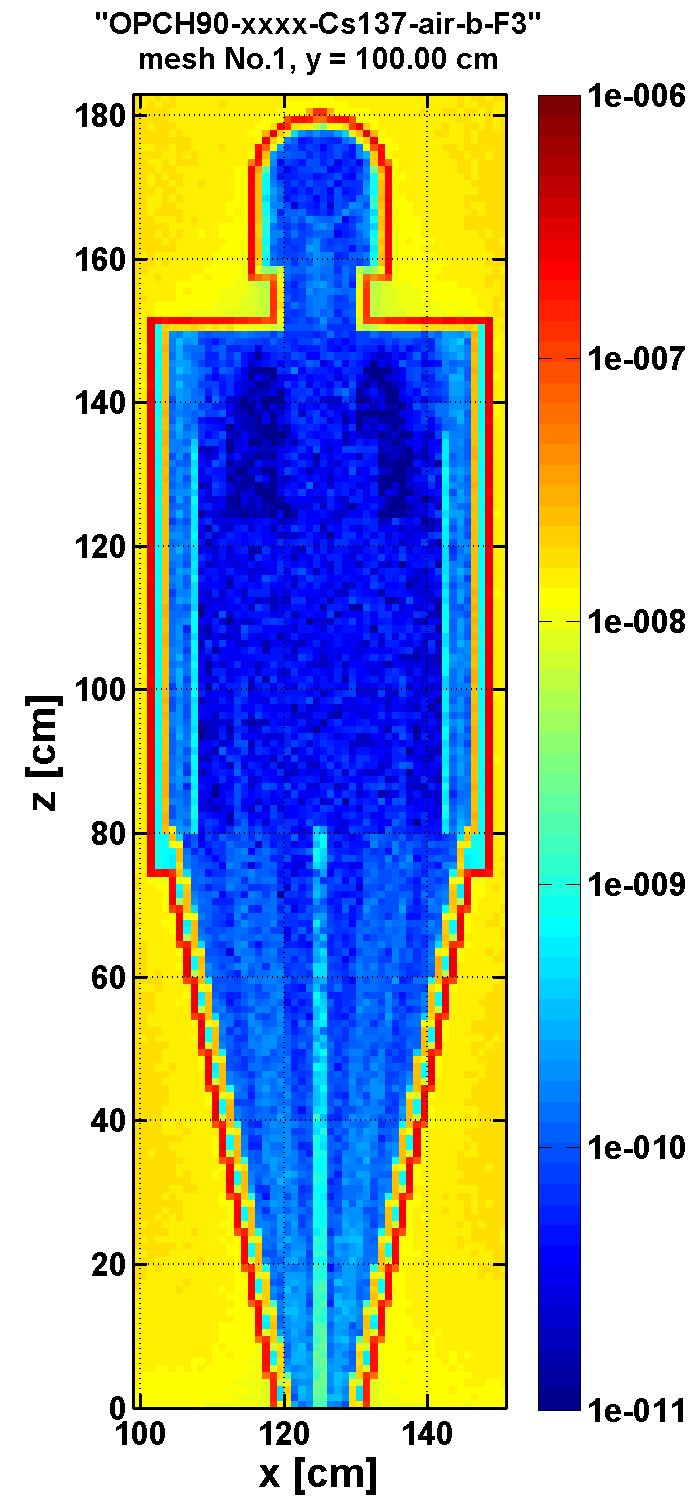

Supplement: Supplementary Materials — The electronic annex presents (1) numerical decrease of the various organs' contribution to the effective dose when protected with individual tested PPE exposed to the other simulated radionuclides dispersed in 10 m3 of the atmosphere in the RAC geometry (Table 2) and (2) visualisations of simulated ORNL phantom energy depositions while only wearing PPE preventing radioactive contamination, and the same PPE together with individual PPE protecting against X- and gamma-ray under it, in a various-dispersed radionuclide's aerosol atmosphere. [file 1641895.f1.zip › 1641895.f1/Electronic annex/Visualization of 2D distributions/Beta contribution/XZ/XZ-b-air-Cs137-OPCH90.png]

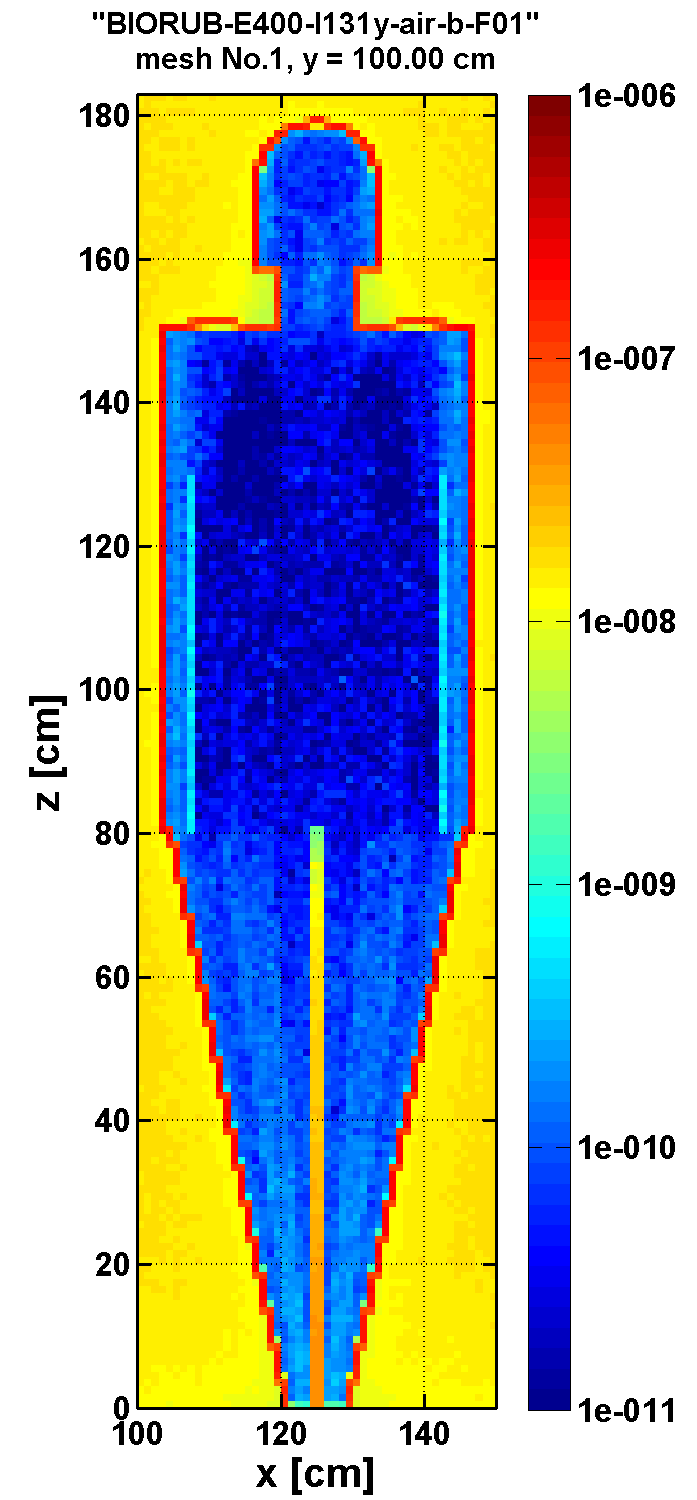

Supplement: Supplementary Materials — The electronic annex presents (1) numerical decrease of the various organs' contribution to the effective dose when protected with individual tested PPE exposed to the other simulated radionuclides dispersed in 10 m3 of the atmosphere in the RAC geometry (Table 2) and (2) visualisations of simulated ORNL phantom energy depositions while only wearing PPE preventing radioactive contamination, and the same PPE together with individual PPE protecting against X- and gamma-ray under it, in a various-dispersed radionuclide's aerosol atmosphere. [file 1641895.f1.zip › 1641895.f1/Electronic annex/Visualization of 2D distributions/Beta contribution/XZ/XZ-b-air-I131-BIORUB-E400.png]

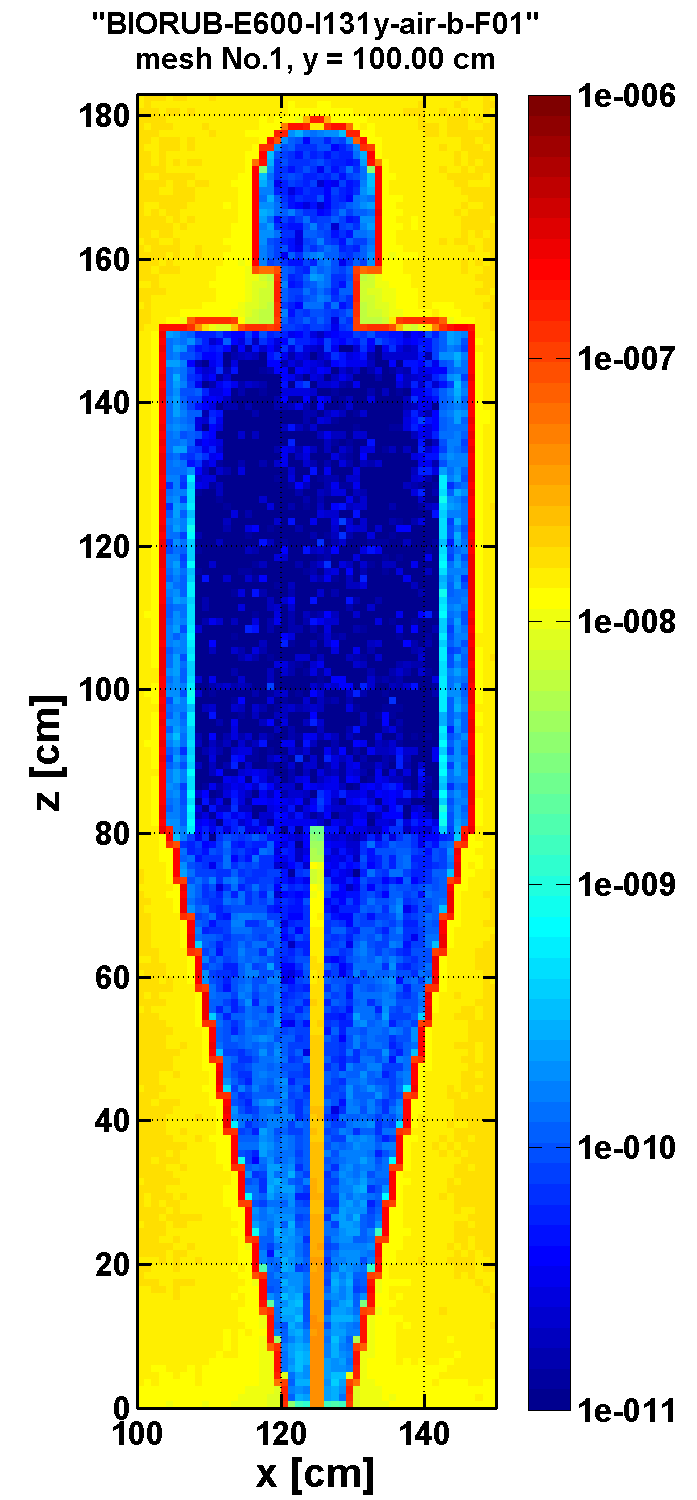

Supplement: Supplementary Materials — The electronic annex presents (1) numerical decrease of the various organs' contribution to the effective dose when protected with individual tested PPE exposed to the other simulated radionuclides dispersed in 10 m3 of the atmosphere in the RAC geometry (Table 2) and (2) visualisations of simulated ORNL phantom energy depositions while only wearing PPE preventing radioactive contamination, and the same PPE together with individual PPE protecting against X- and gamma-ray under it, in a various-dispersed radionuclide's aerosol atmosphere. [file 1641895.f1.zip › 1641895.f1/Electronic annex/Visualization of 2D distributions/Beta contribution/XZ/XZ-b-air-I131-BIORUB-E600.png]

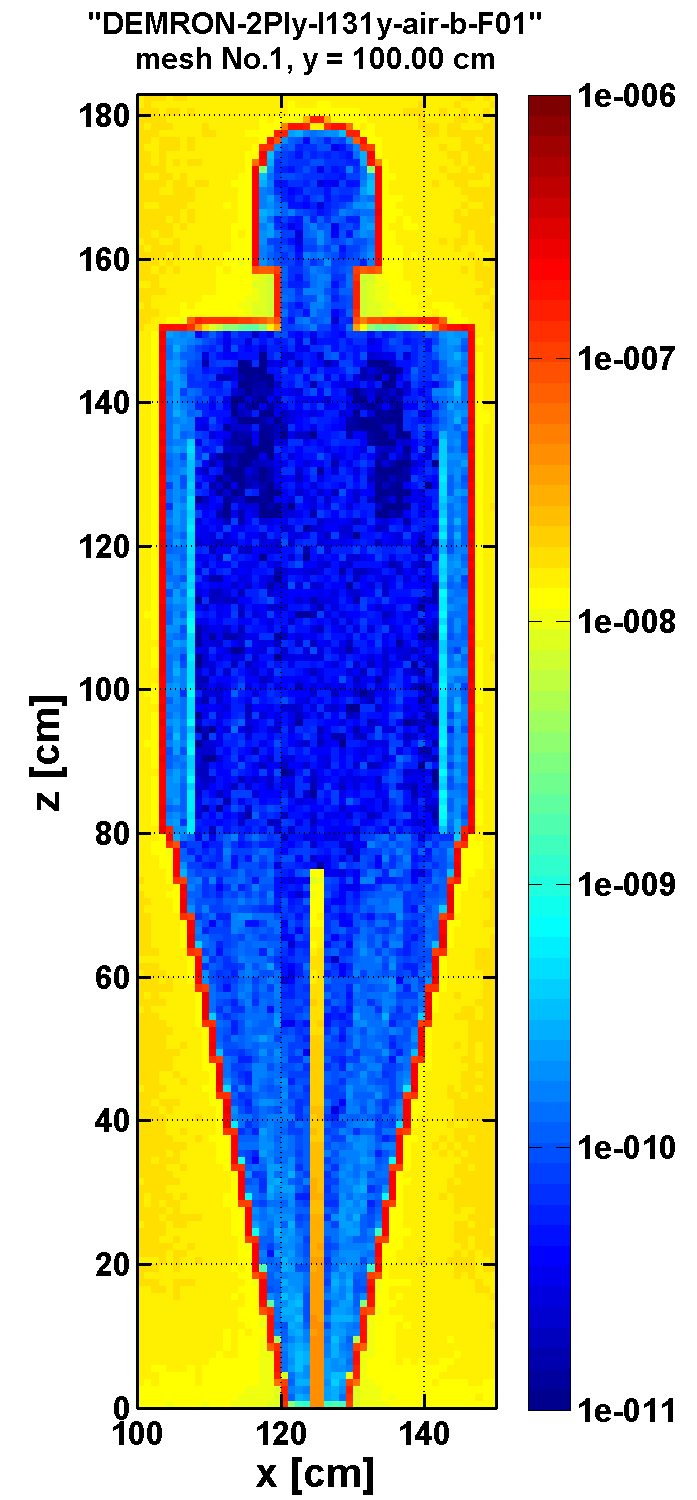

Supplement: Supplementary Materials — The electronic annex presents (1) numerical decrease of the various organs' contribution to the effective dose when protected with individual tested PPE exposed to the other simulated radionuclides dispersed in 10 m3 of the atmosphere in the RAC geometry (Table 2) and (2) visualisations of simulated ORNL phantom energy depositions while only wearing PPE preventing radioactive contamination, and the same PPE together with individual PPE protecting against X- and gamma-ray under it, in a various-dispersed radionuclide's aerosol atmosphere. [file 1641895.f1.zip › 1641895.f1/Electronic annex/Visualization of 2D distributions/Beta contribution/XZ/XZ-b-air-I131-DEMRON-2Ply.png]

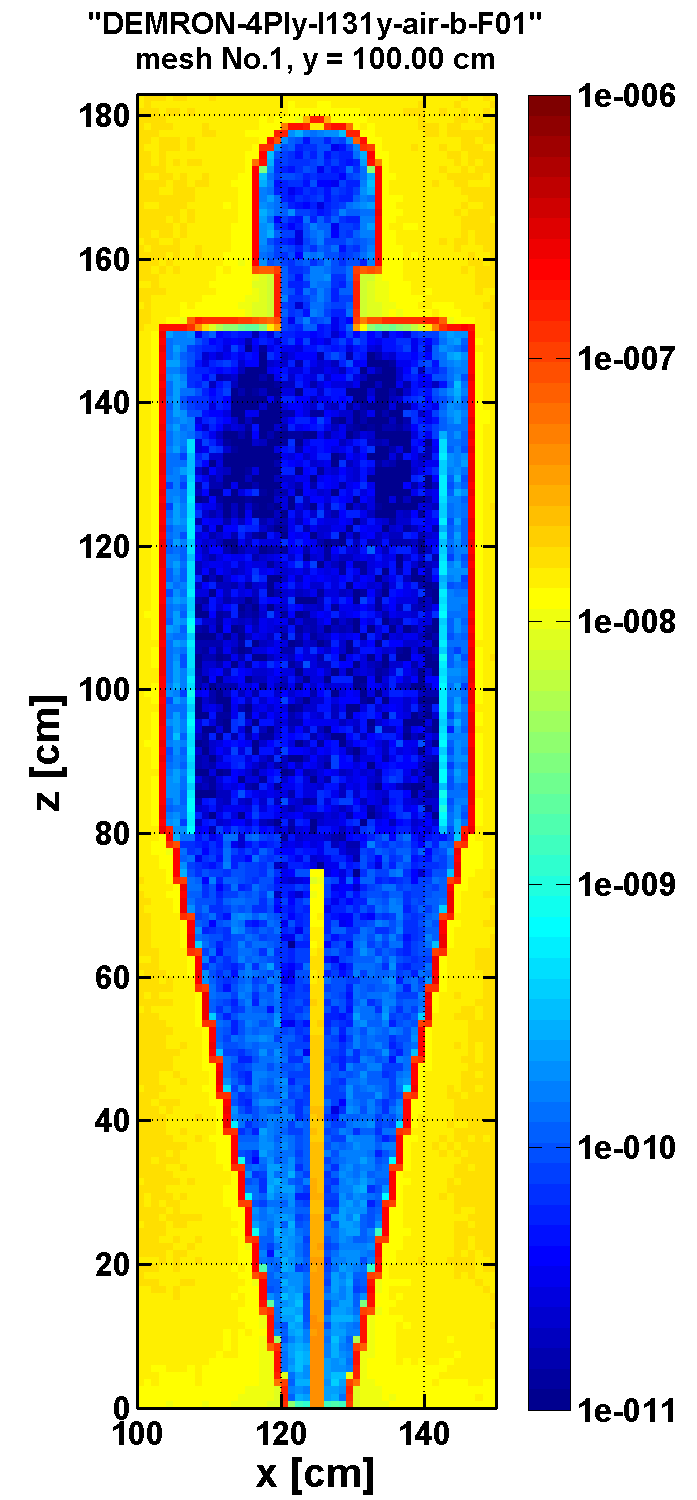

Supplement: Supplementary Materials — The electronic annex presents (1) numerical decrease of the various organs' contribution to the effective dose when protected with individual tested PPE exposed to the other simulated radionuclides dispersed in 10 m3 of the atmosphere in the RAC geometry (Table 2) and (2) visualisations of simulated ORNL phantom energy depositions while only wearing PPE preventing radioactive contamination, and the same PPE together with individual PPE protecting against X- and gamma-ray under it, in a various-dispersed radionuclide's aerosol atmosphere. [file 1641895.f1.zip › 1641895.f1/Electronic annex/Visualization of 2D distributions/Beta contribution/XZ/XZ-b-air-I131-DEMRON-4Ply.png]

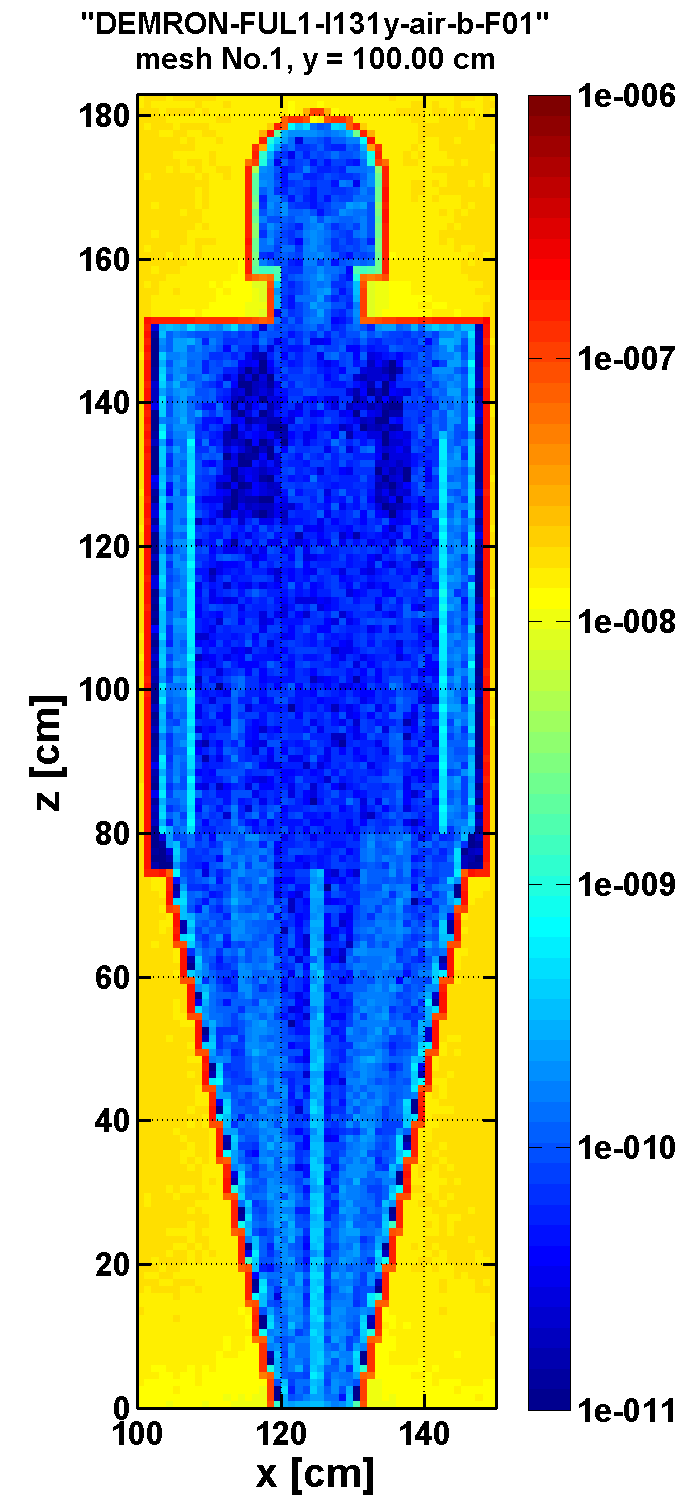

Supplement: Supplementary Materials — The electronic annex presents (1) numerical decrease of the various organs' contribution to the effective dose when protected with individual tested PPE exposed to the other simulated radionuclides dispersed in 10 m3 of the atmosphere in the RAC geometry (Table 2) and (2) visualisations of simulated ORNL phantom energy depositions while only wearing PPE preventing radioactive contamination, and the same PPE together with individual PPE protecting against X- and gamma-ray under it, in a various-dispersed radionuclide's aerosol atmosphere. [file 1641895.f1.zip › 1641895.f1/Electronic annex/Visualization of 2D distributions/Beta contribution/XZ/XZ-b-air-I131-DEMRON-FUL1.png]

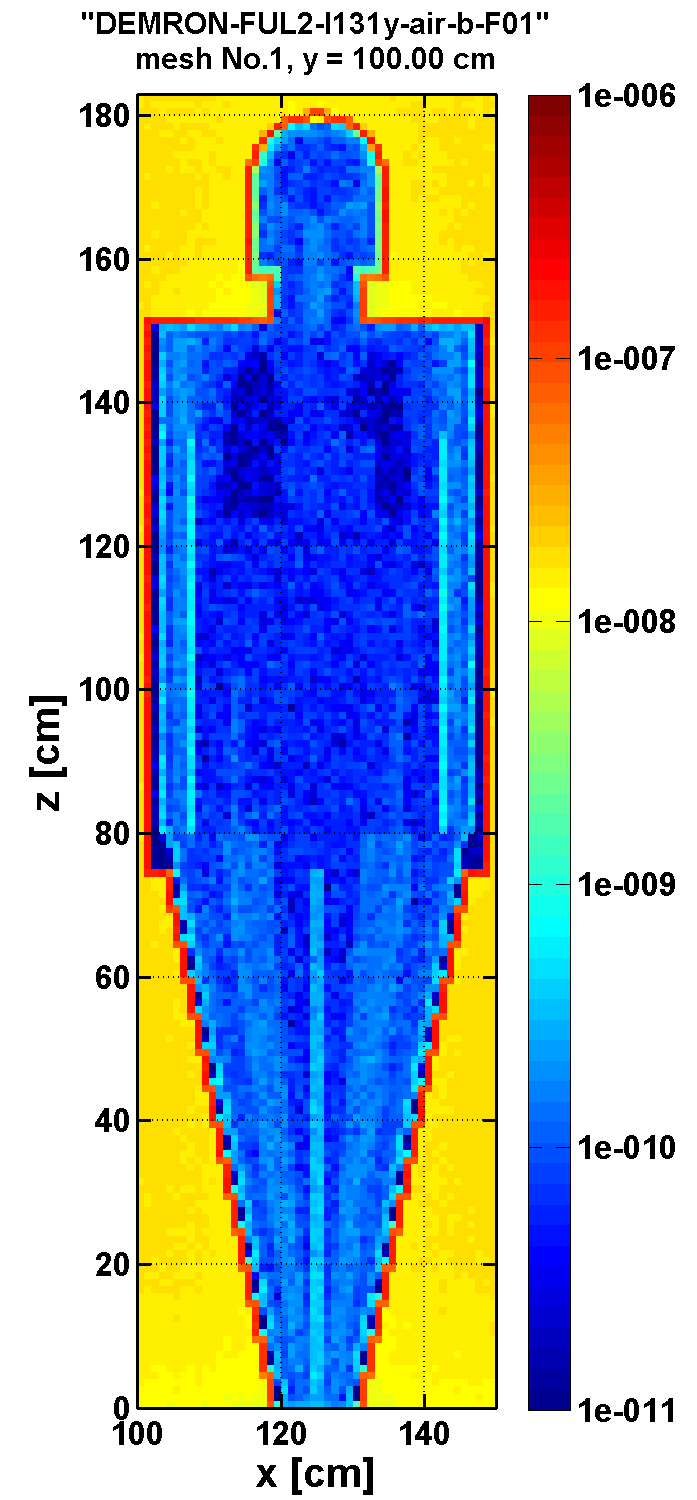

Supplement: Supplementary Materials — The electronic annex presents (1) numerical decrease of the various organs' contribution to the effective dose when protected with individual tested PPE exposed to the other simulated radionuclides dispersed in 10 m3 of the atmosphere in the RAC geometry (Table 2) and (2) visualisations of simulated ORNL phantom energy depositions while only wearing PPE preventing radioactive contamination, and the same PPE together with individual PPE protecting against X- and gamma-ray under it, in a various-dispersed radionuclide's aerosol atmosphere. [file 1641895.f1.zip › 1641895.f1/Electronic annex/Visualization of 2D distributions/Beta contribution/XZ/XZ-b-air-I131-DEMRON-FUL2.png]

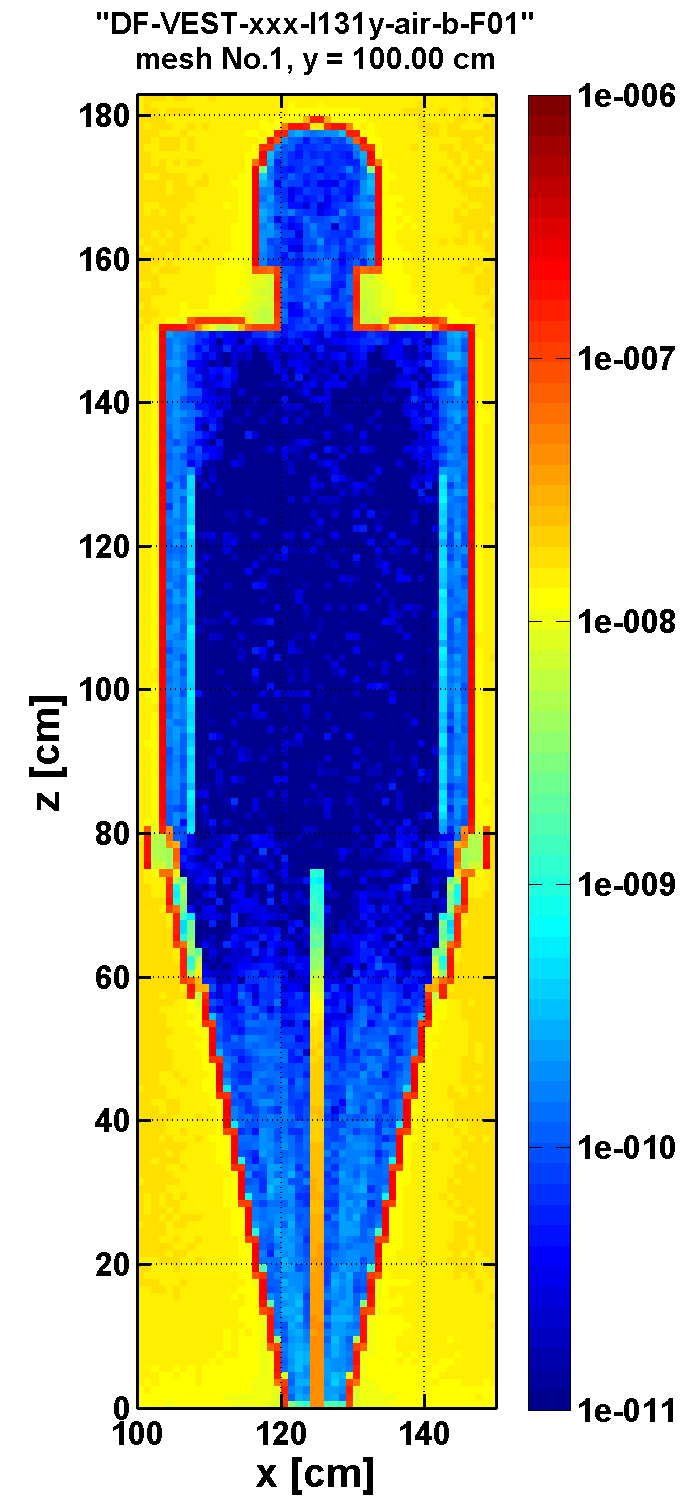

Supplement: Supplementary Materials — The electronic annex presents (1) numerical decrease of the various organs' contribution to the effective dose when protected with individual tested PPE exposed to the other simulated radionuclides dispersed in 10 m3 of the atmosphere in the RAC geometry (Table 2) and (2) visualisations of simulated ORNL phantom energy depositions while only wearing PPE preventing radioactive contamination, and the same PPE together with individual PPE protecting against X- and gamma-ray under it, in a various-dispersed radionuclide's aerosol atmosphere. [file 1641895.f1.zip › 1641895.f1/Electronic annex/Visualization of 2D distributions/Beta contribution/XZ/XZ-b-air-I131-DF-VEST.png]

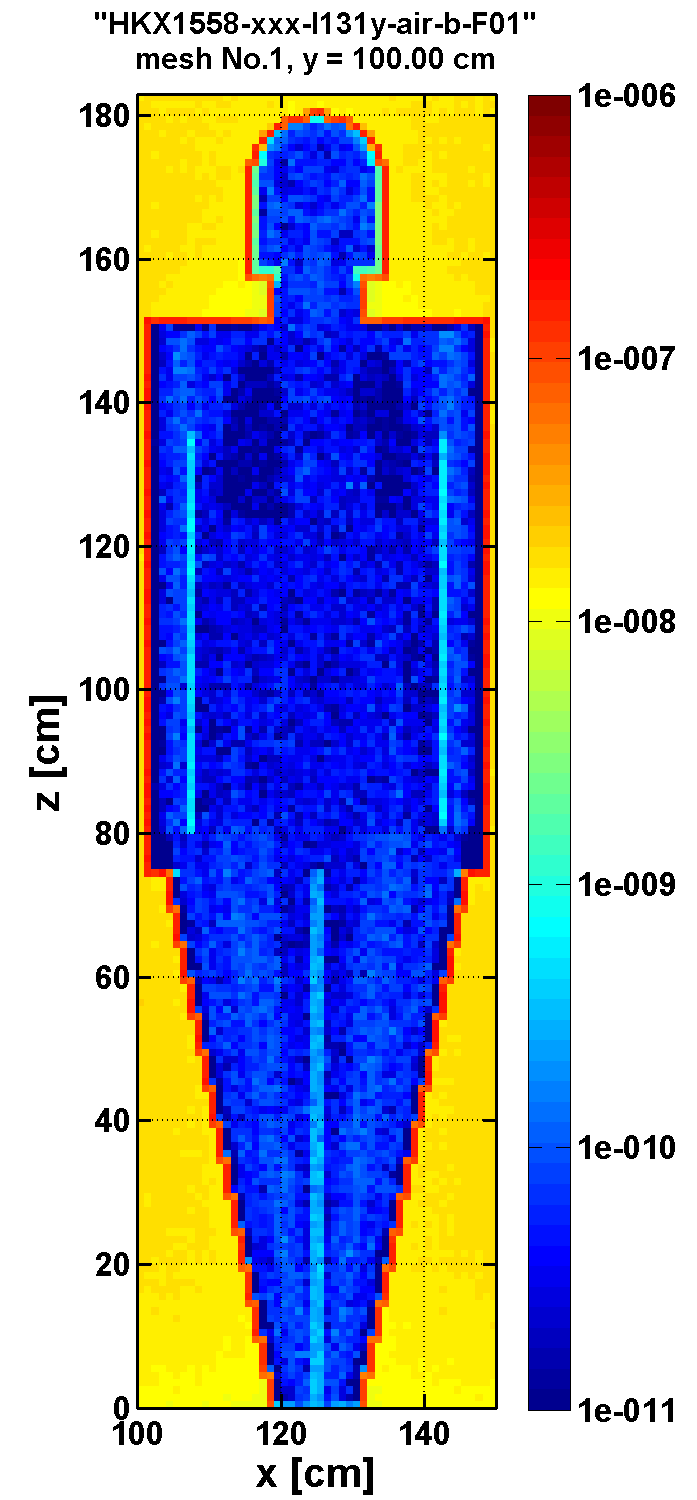

Supplement: Supplementary Materials — The electronic annex presents (1) numerical decrease of the various organs' contribution to the effective dose when protected with individual tested PPE exposed to the other simulated radionuclides dispersed in 10 m3 of the atmosphere in the RAC geometry (Table 2) and (2) visualisations of simulated ORNL phantom energy depositions while only wearing PPE preventing radioactive contamination, and the same PPE together with individual PPE protecting against X- and gamma-ray under it, in a various-dispersed radionuclide's aerosol atmosphere. [file 1641895.f1.zip › 1641895.f1/Electronic annex/Visualization of 2D distributions/Beta contribution/XZ/XZ-b-air-I131-HKX1558.png]

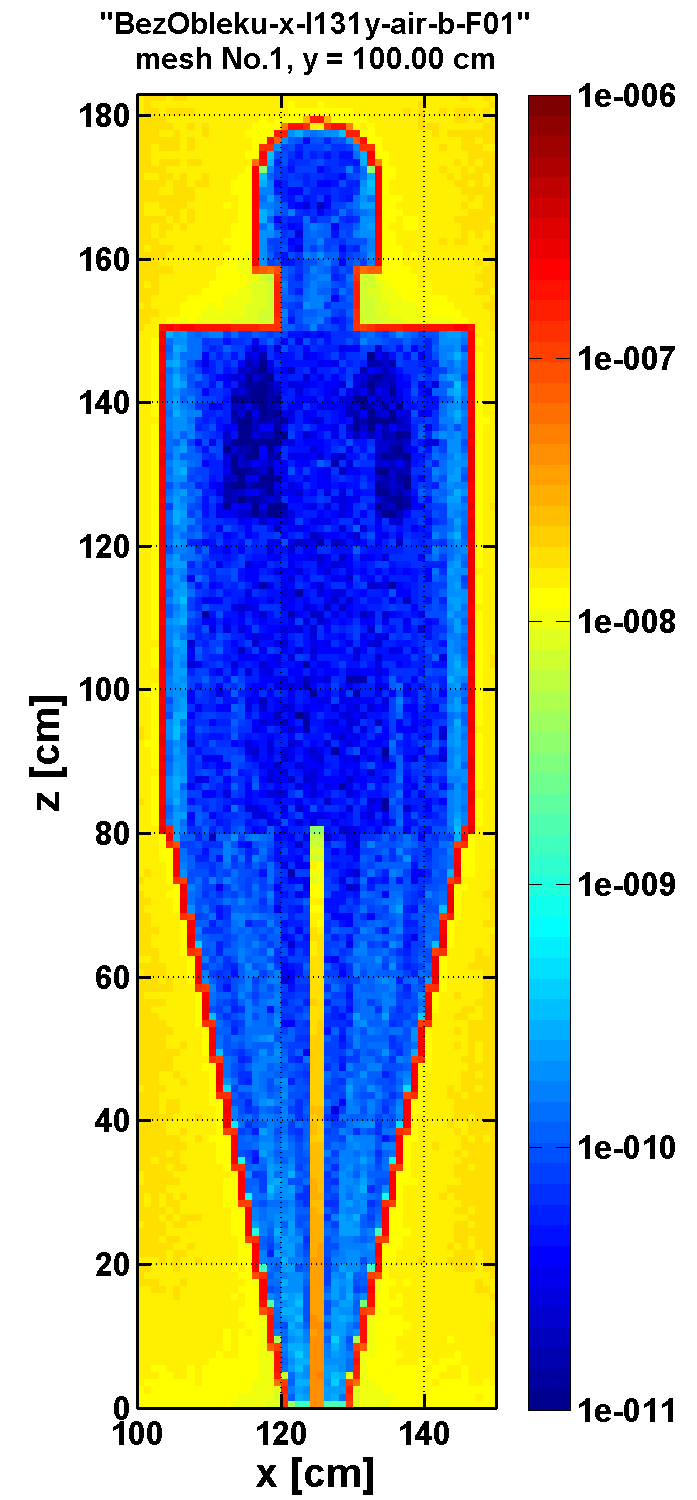

Supplement: Supplementary Materials — The electronic annex presents (1) numerical decrease of the various organs' contribution to the effective dose when protected with individual tested PPE exposed to the other simulated radionuclides dispersed in 10 m3 of the atmosphere in the RAC geometry (Table 2) and (2) visualisations of simulated ORNL phantom energy depositions while only wearing PPE preventing radioactive contamination, and the same PPE together with individual PPE protecting against X- and gamma-ray under it, in a various-dispersed radionuclide's aerosol atmosphere. [file 1641895.f1.zip › 1641895.f1/Electronic annex/Visualization of 2D distributions/Beta contribution/XZ/XZ-b-air-I131-NoPPE.png]

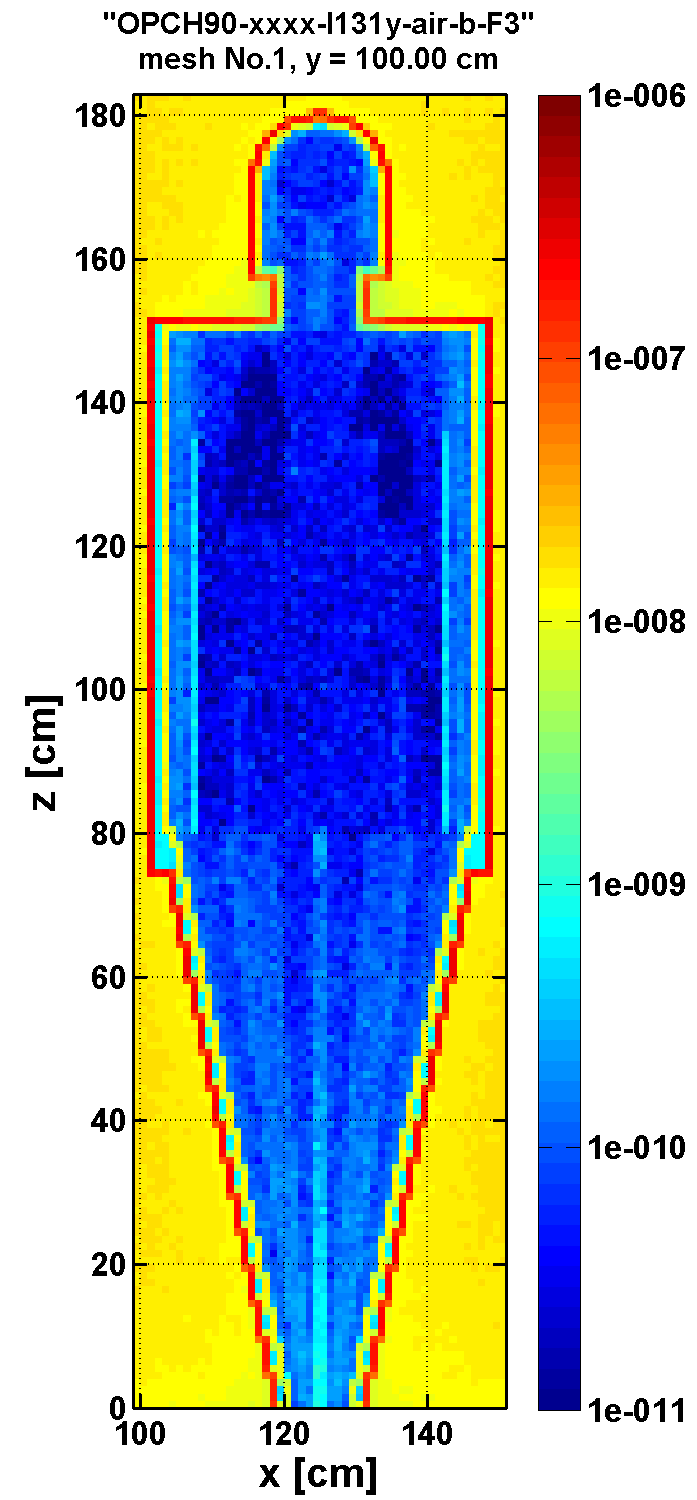

Supplement: Supplementary Materials — The electronic annex presents (1) numerical decrease of the various organs' contribution to the effective dose when protected with individual tested PPE exposed to the other simulated radionuclides dispersed in 10 m3 of the atmosphere in the RAC geometry (Table 2) and (2) visualisations of simulated ORNL phantom energy depositions while only wearing PPE preventing radioactive contamination, and the same PPE together with individual PPE protecting against X- and gamma-ray under it, in a various-dispersed radionuclide's aerosol atmosphere. [file 1641895.f1.zip › 1641895.f1/Electronic annex/Visualization of 2D distributions/Beta contribution/XZ/XZ-b-air-I131-OPCH90.png]

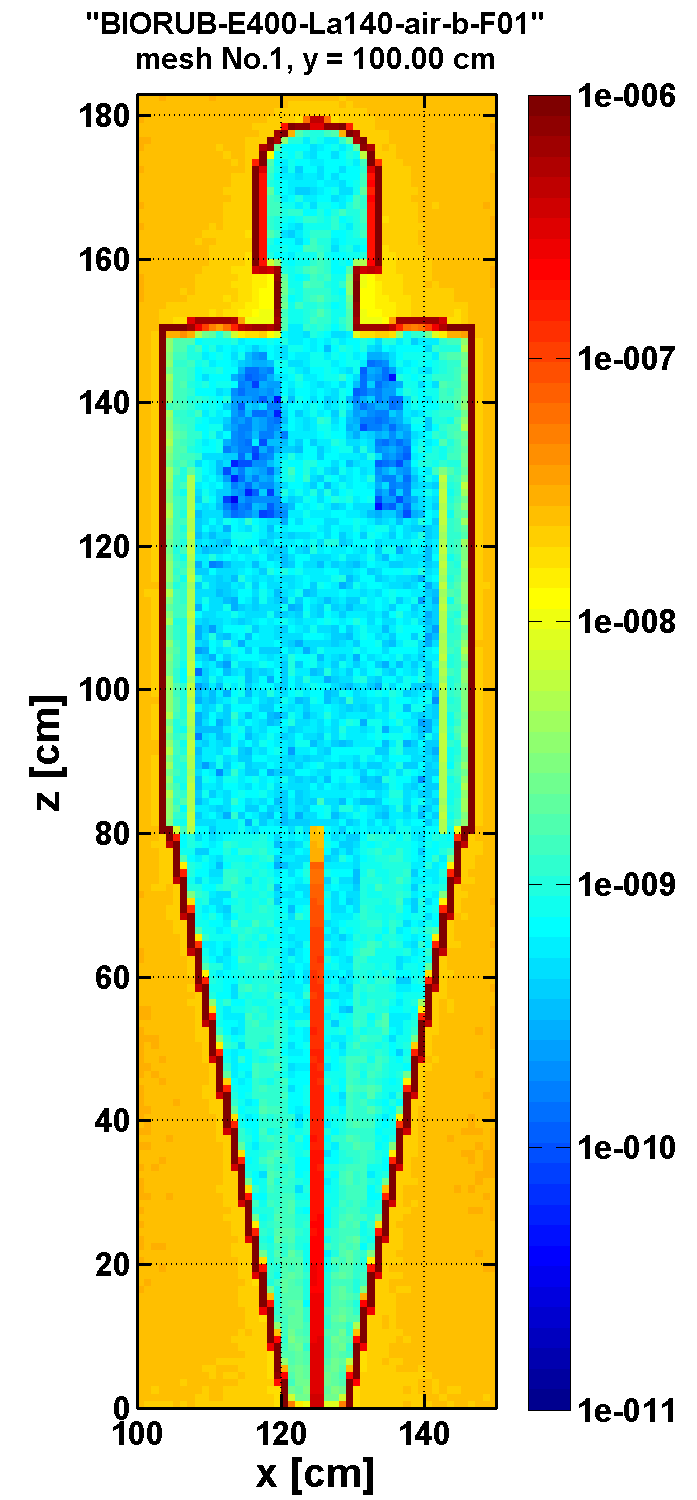

Supplement: Supplementary Materials — The electronic annex presents (1) numerical decrease of the various organs' contribution to the effective dose when protected with individual tested PPE exposed to the other simulated radionuclides dispersed in 10 m3 of the atmosphere in the RAC geometry (Table 2) and (2) visualisations of simulated ORNL phantom energy depositions while only wearing PPE preventing radioactive contamination, and the same PPE together with individual PPE protecting against X- and gamma-ray under it, in a various-dispersed radionuclide's aerosol atmosphere. [file 1641895.f1.zip › 1641895.f1/Electronic annex/Visualization of 2D distributions/Beta contribution/XZ/XZ-b-air-La140-BIORUB-E400.png]

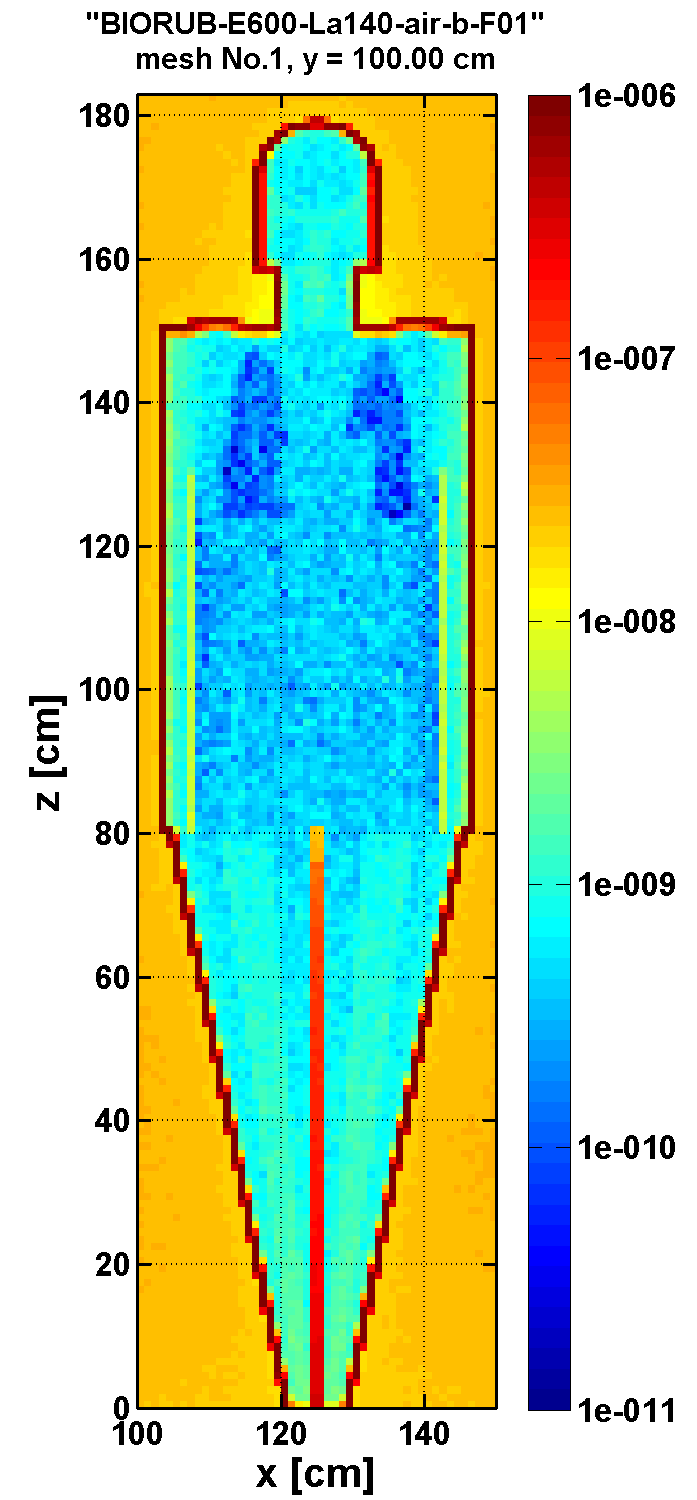

Supplement: Supplementary Materials — The electronic annex presents (1) numerical decrease of the various organs' contribution to the effective dose when protected with individual tested PPE exposed to the other simulated radionuclides dispersed in 10 m3 of the atmosphere in the RAC geometry (Table 2) and (2) visualisations of simulated ORNL phantom energy depositions while only wearing PPE preventing radioactive contamination, and the same PPE together with individual PPE protecting against X- and gamma-ray under it, in a various-dispersed radionuclide's aerosol atmosphere. [file 1641895.f1.zip › 1641895.f1/Electronic annex/Visualization of 2D distributions/Beta contribution/XZ/XZ-b-air-La140-BIORUB-E600.png]

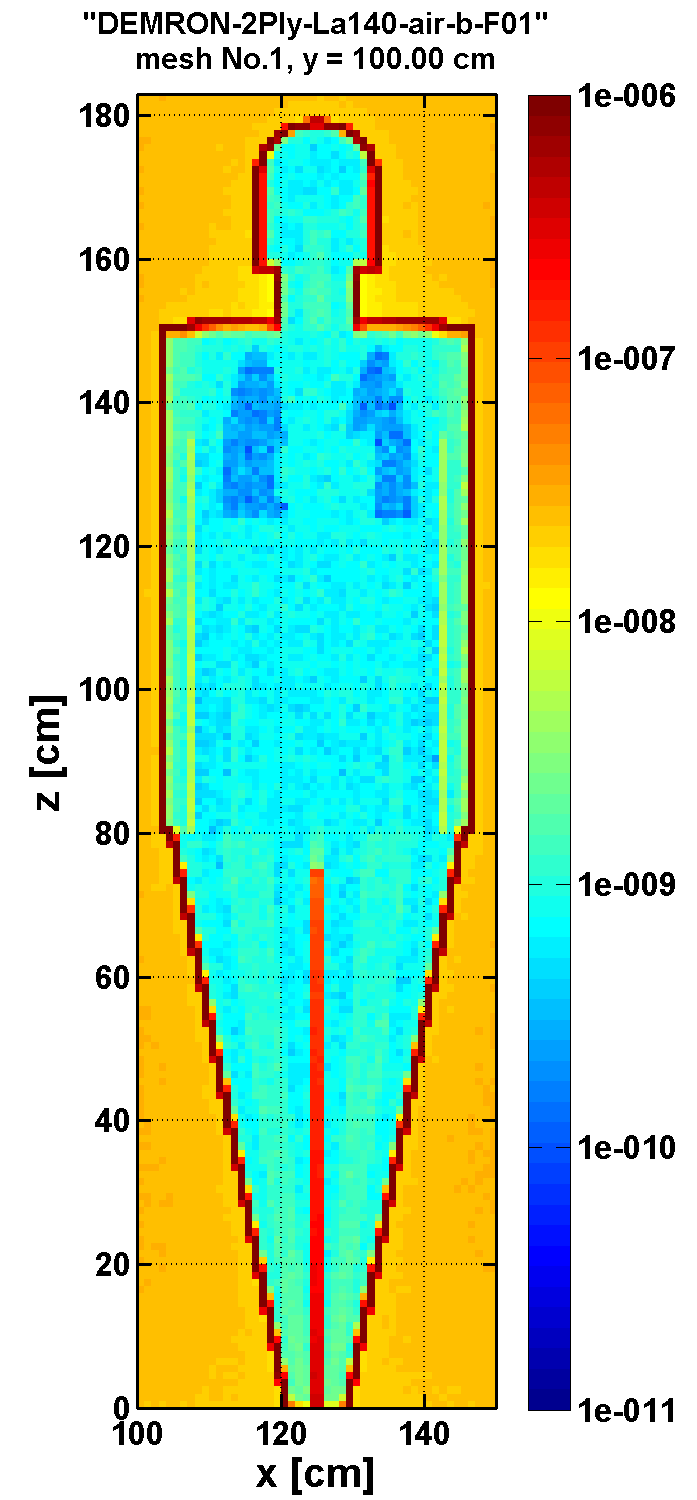

Supplement: Supplementary Materials — The electronic annex presents (1) numerical decrease of the various organs' contribution to the effective dose when protected with individual tested PPE exposed to the other simulated radionuclides dispersed in 10 m3 of the atmosphere in the RAC geometry (Table 2) and (2) visualisations of simulated ORNL phantom energy depositions while only wearing PPE preventing radioactive contamination, and the same PPE together with individual PPE protecting against X- and gamma-ray under it, in a various-dispersed radionuclide's aerosol atmosphere. [file 1641895.f1.zip › 1641895.f1/Electronic annex/Visualization of 2D distributions/Beta contribution/XZ/XZ-b-air-La140-DEMRON-2Ply.png]

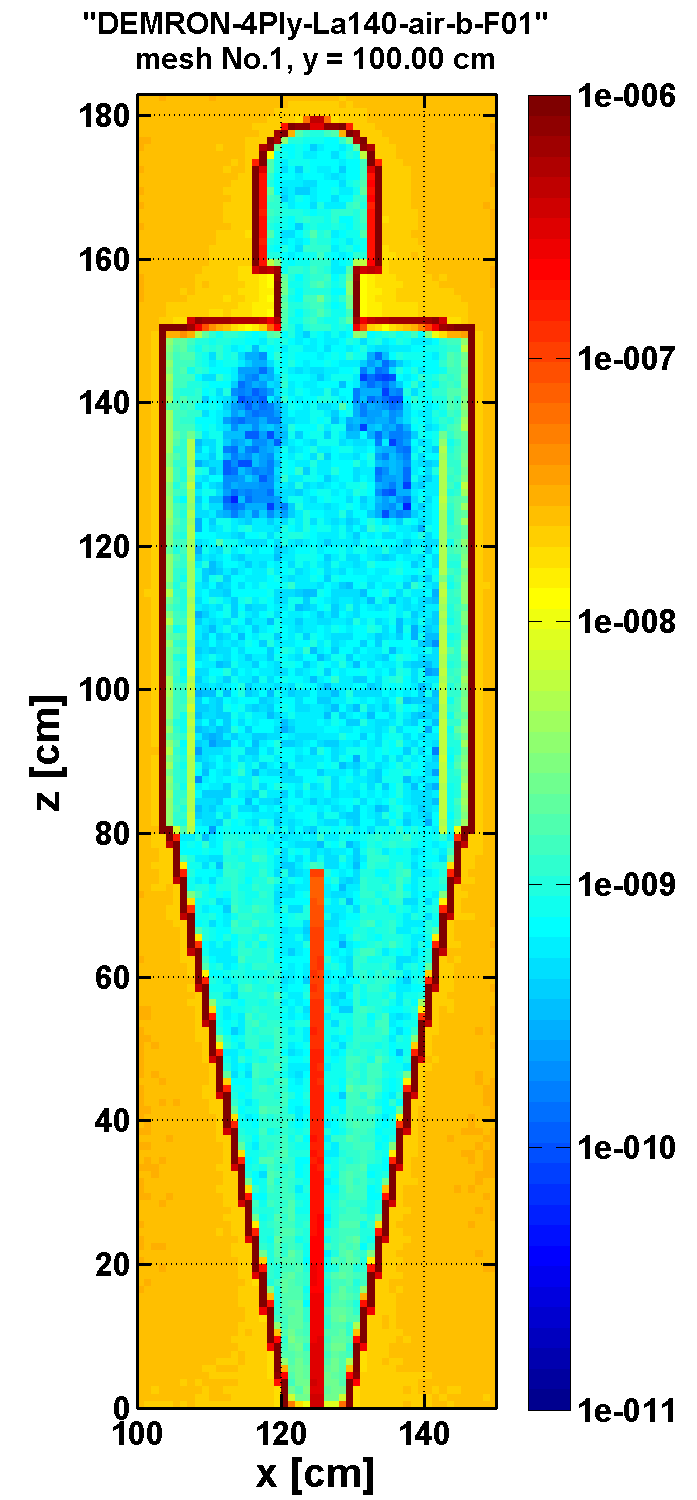

Supplement: Supplementary Materials — The electronic annex presents (1) numerical decrease of the various organs' contribution to the effective dose when protected with individual tested PPE exposed to the other simulated radionuclides dispersed in 10 m3 of the atmosphere in the RAC geometry (Table 2) and (2) visualisations of simulated ORNL phantom energy depositions while only wearing PPE preventing radioactive contamination, and the same PPE together with individual PPE protecting against X- and gamma-ray under it, in a various-dispersed radionuclide's aerosol atmosphere. [file 1641895.f1.zip › 1641895.f1/Electronic annex/Visualization of 2D distributions/Beta contribution/XZ/XZ-b-air-La140-DEMRON-4Ply.png]

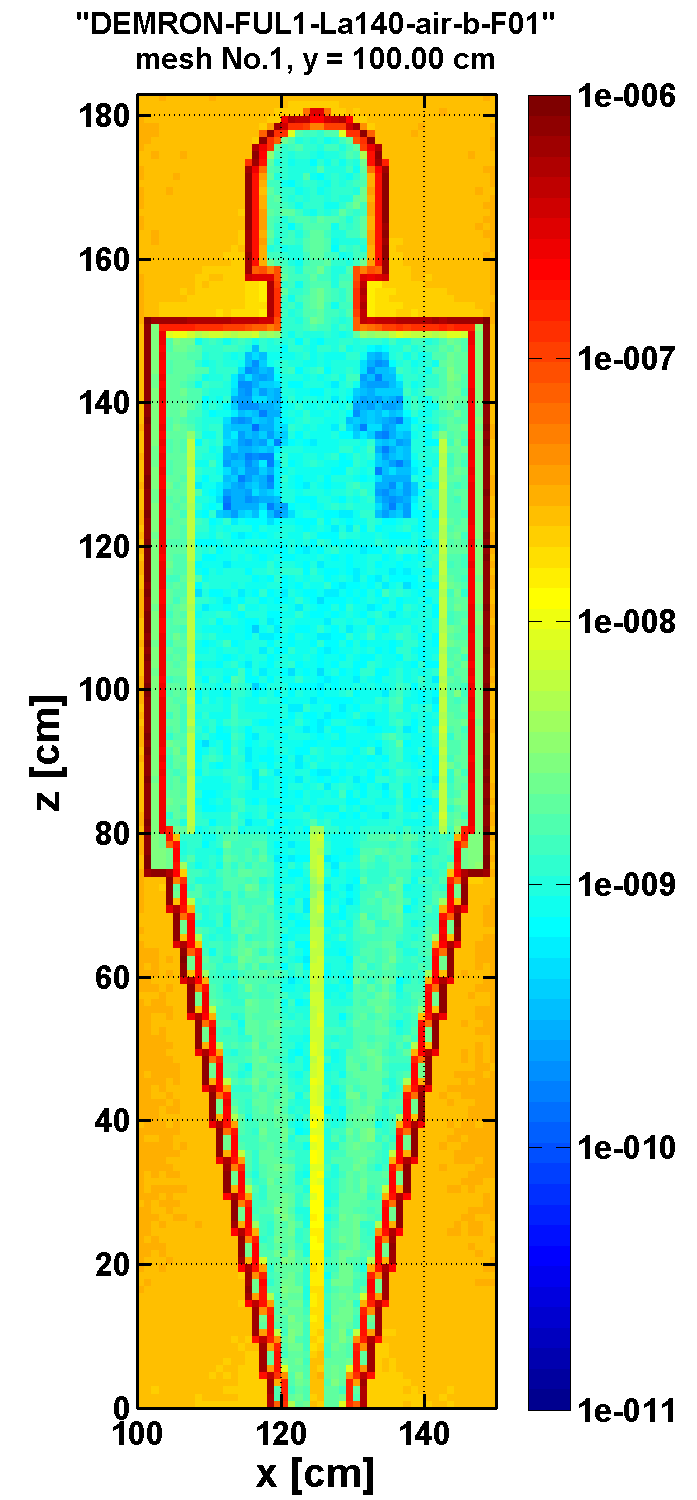

Supplement: Supplementary Materials — The electronic annex presents (1) numerical decrease of the various organs' contribution to the effective dose when protected with individual tested PPE exposed to the other simulated radionuclides dispersed in 10 m3 of the atmosphere in the RAC geometry (Table 2) and (2) visualisations of simulated ORNL phantom energy depositions while only wearing PPE preventing radioactive contamination, and the same PPE together with individual PPE protecting against X- and gamma-ray under it, in a various-dispersed radionuclide's aerosol atmosphere. [file 1641895.f1.zip › 1641895.f1/Electronic annex/Visualization of 2D distributions/Beta contribution/XZ/XZ-b-air-La140-DEMRON-FUL1.png]

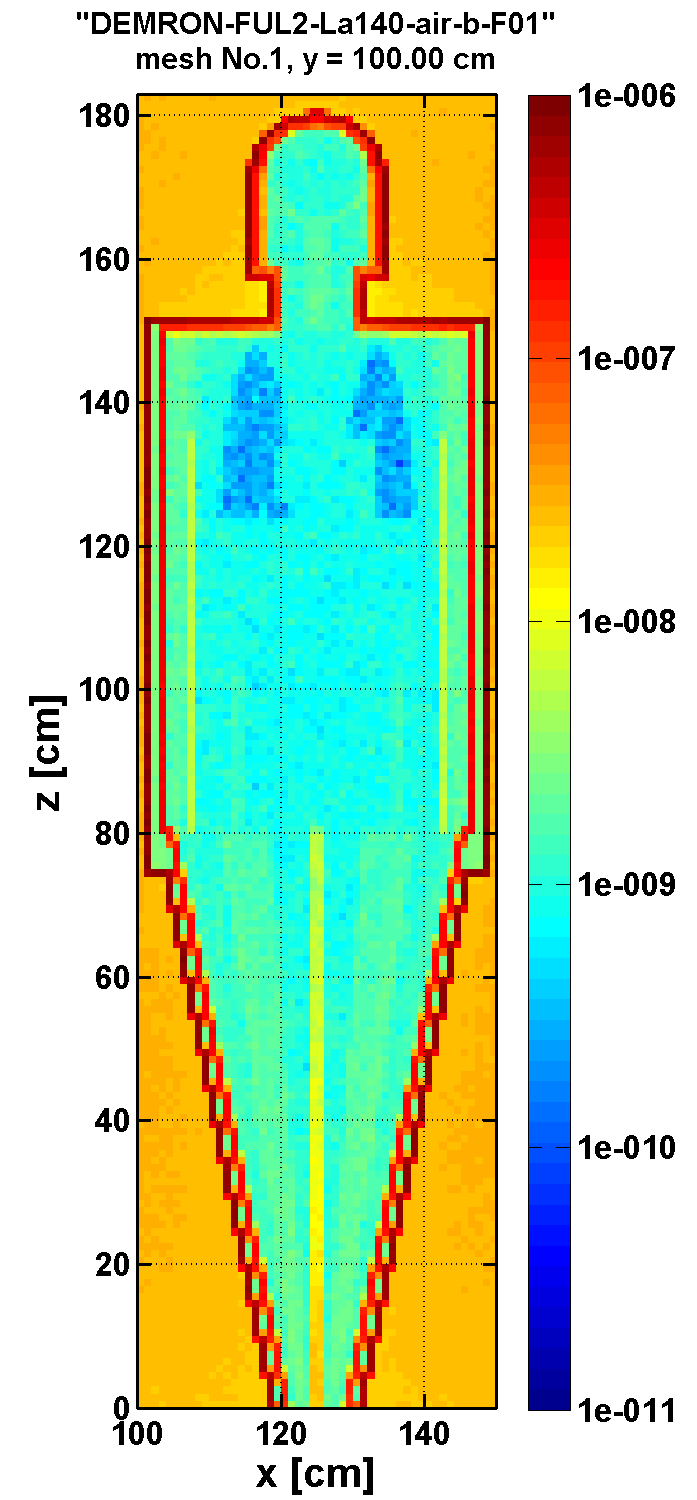

Supplement: Supplementary Materials — The electronic annex presents (1) numerical decrease of the various organs' contribution to the effective dose when protected with individual tested PPE exposed to the other simulated radionuclides dispersed in 10 m3 of the atmosphere in the RAC geometry (Table 2) and (2) visualisations of simulated ORNL phantom energy depositions while only wearing PPE preventing radioactive contamination, and the same PPE together with individual PPE protecting against X- and gamma-ray under it, in a various-dispersed radionuclide's aerosol atmosphere. [file 1641895.f1.zip › 1641895.f1/Electronic annex/Visualization of 2D distributions/Beta contribution/XZ/XZ-b-air-La140-DEMRON-FUL2.png]

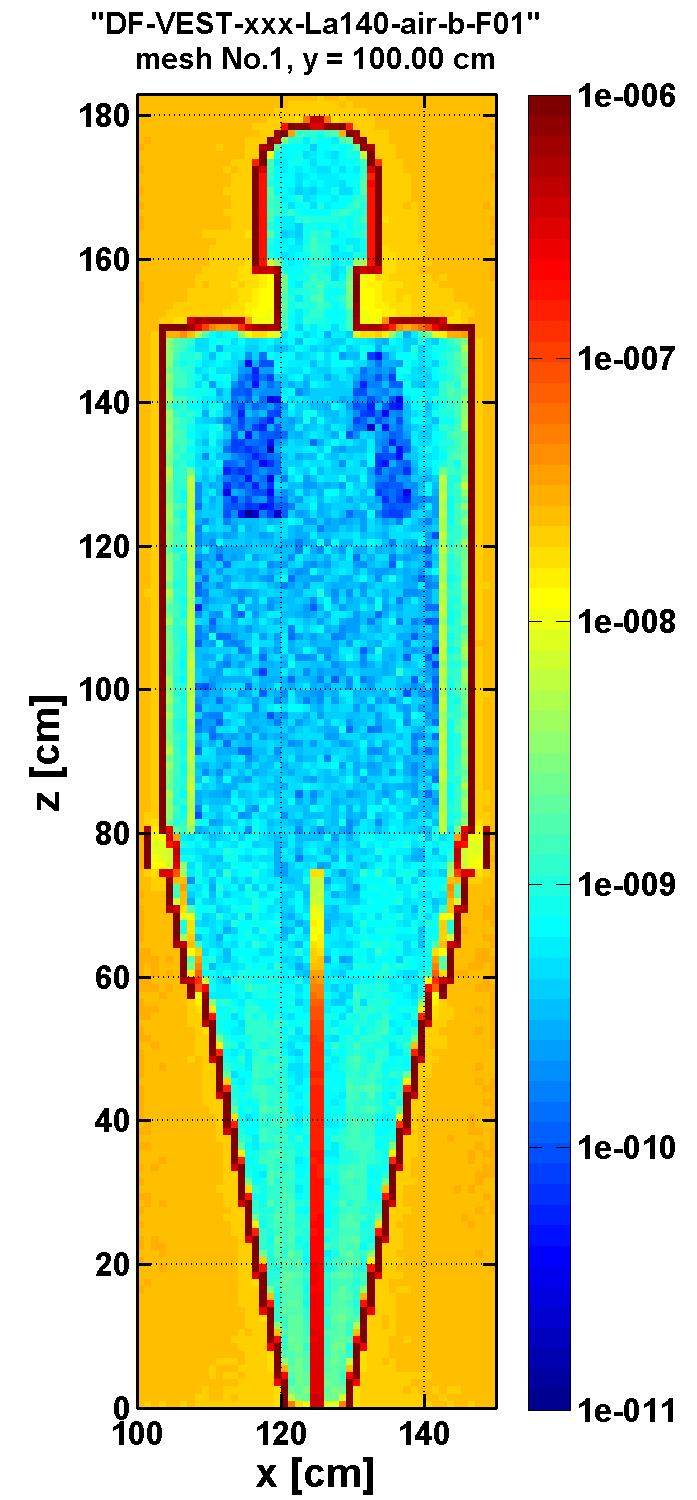

Supplement: Supplementary Materials — The electronic annex presents (1) numerical decrease of the various organs' contribution to the effective dose when protected with individual tested PPE exposed to the other simulated radionuclides dispersed in 10 m3 of the atmosphere in the RAC geometry (Table 2) and (2) visualisations of simulated ORNL phantom energy depositions while only wearing PPE preventing radioactive contamination, and the same PPE together with individual PPE protecting against X- and gamma-ray under it, in a various-dispersed radionuclide's aerosol atmosphere. [file 1641895.f1.zip › 1641895.f1/Electronic annex/Visualization of 2D distributions/Beta contribution/XZ/XZ-b-air-La140-DF-VEST.png]

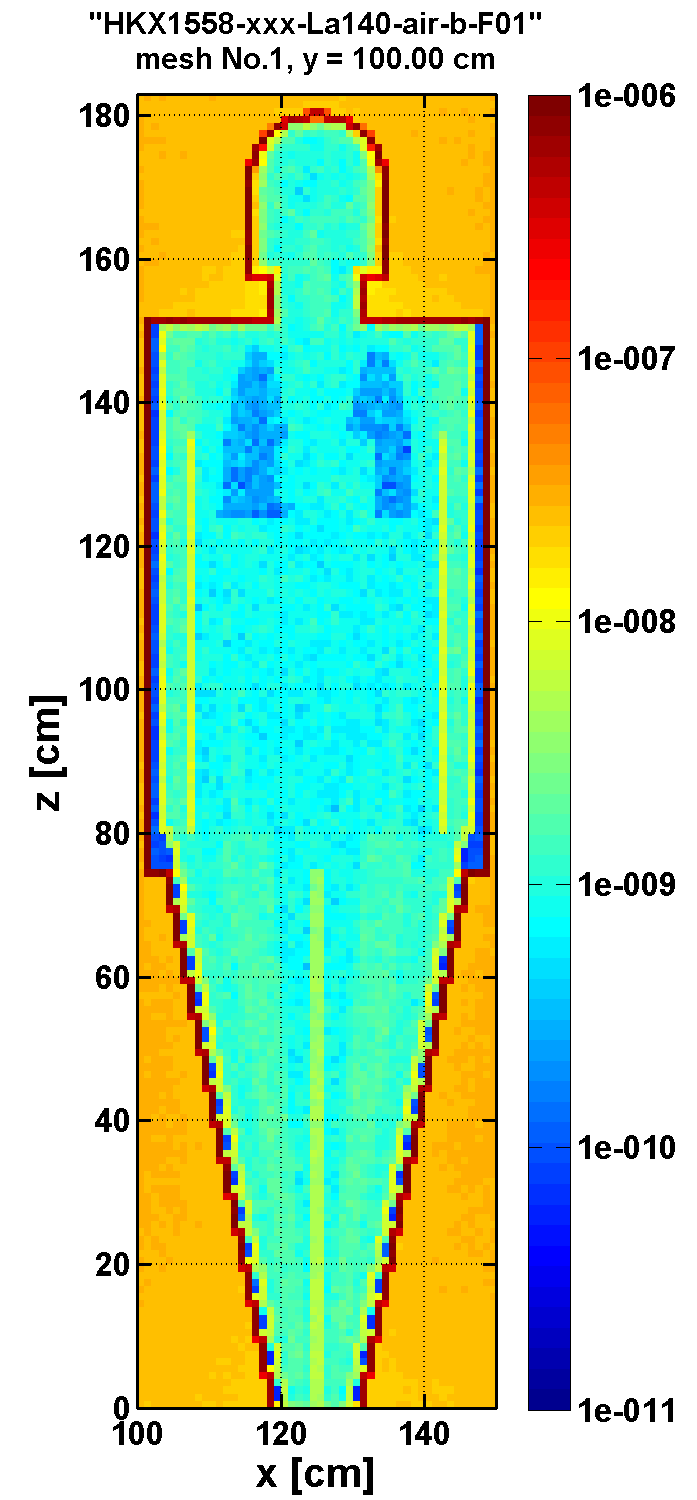

Supplement: Supplementary Materials — The electronic annex presents (1) numerical decrease of the various organs' contribution to the effective dose when protected with individual tested PPE exposed to the other simulated radionuclides dispersed in 10 m3 of the atmosphere in the RAC geometry (Table 2) and (2) visualisations of simulated ORNL phantom energy depositions while only wearing PPE preventing radioactive contamination, and the same PPE together with individual PPE protecting against X- and gamma-ray under it, in a various-dispersed radionuclide's aerosol atmosphere. [file 1641895.f1.zip › 1641895.f1/Electronic annex/Visualization of 2D distributions/Beta contribution/XZ/XZ-b-air-La140-HKX1558.png]

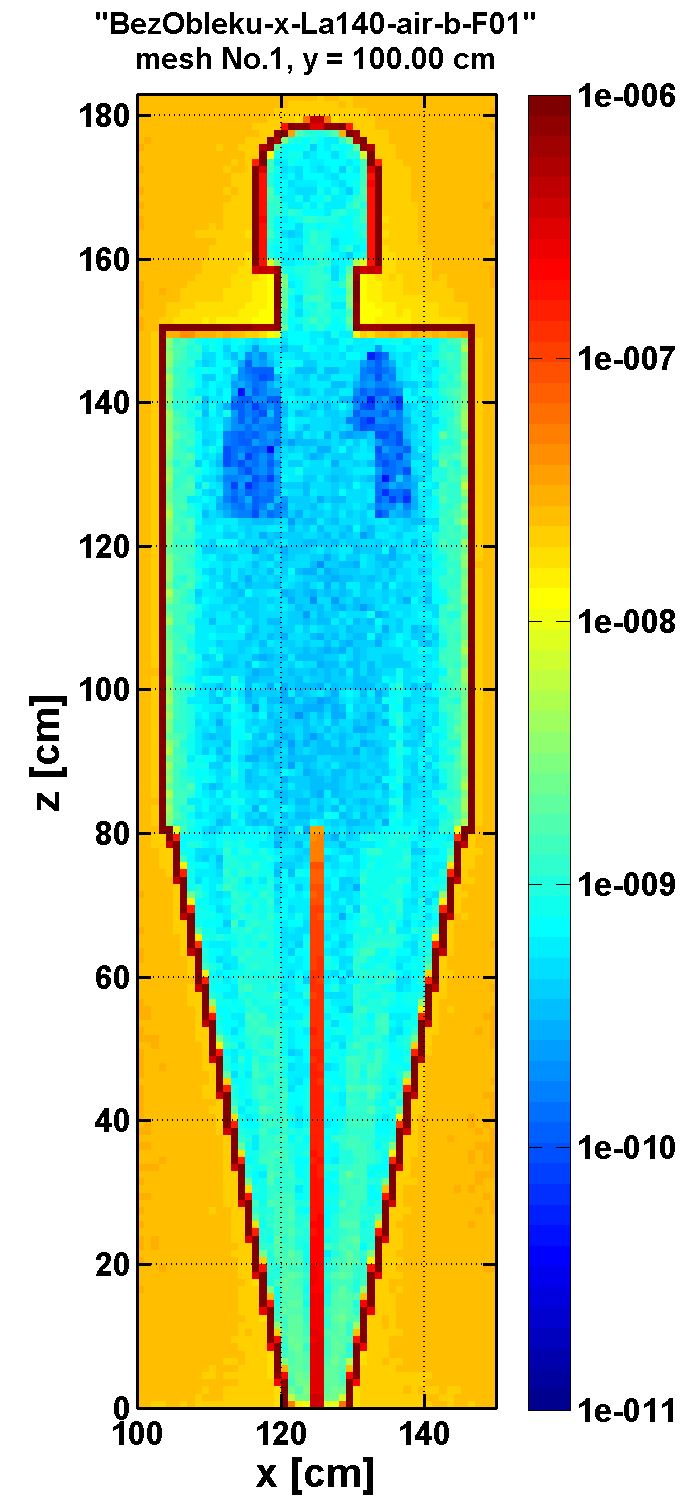

Supplement: Supplementary Materials — The electronic annex presents (1) numerical decrease of the various organs' contribution to the effective dose when protected with individual tested PPE exposed to the other simulated radionuclides dispersed in 10 m3 of the atmosphere in the RAC geometry (Table 2) and (2) visualisations of simulated ORNL phantom energy depositions while only wearing PPE preventing radioactive contamination, and the same PPE together with individual PPE protecting against X- and gamma-ray under it, in a various-dispersed radionuclide's aerosol atmosphere. [file 1641895.f1.zip › 1641895.f1/Electronic annex/Visualization of 2D distributions/Beta contribution/XZ/XZ-b-air-La140-NoPPE.png]

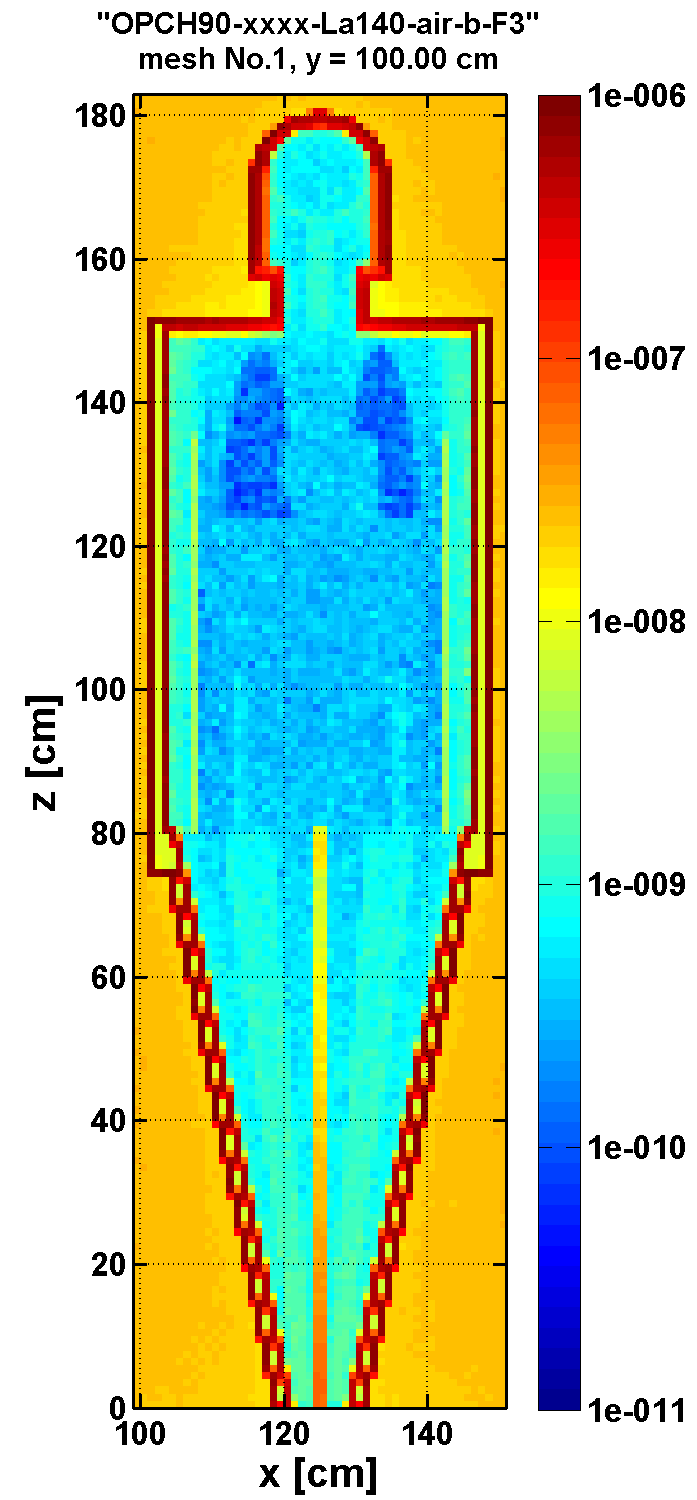

Supplement: Supplementary Materials — The electronic annex presents (1) numerical decrease of the various organs' contribution to the effective dose when protected with individual tested PPE exposed to the other simulated radionuclides dispersed in 10 m3 of the atmosphere in the RAC geometry (Table 2) and (2) visualisations of simulated ORNL phantom energy depositions while only wearing PPE preventing radioactive contamination, and the same PPE together with individual PPE protecting against X- and gamma-ray under it, in a various-dispersed radionuclide's aerosol atmosphere. [file 1641895.f1.zip › 1641895.f1/Electronic annex/Visualization of 2D distributions/Beta contribution/XZ/XZ-b-air-La140-OPCH90.png]

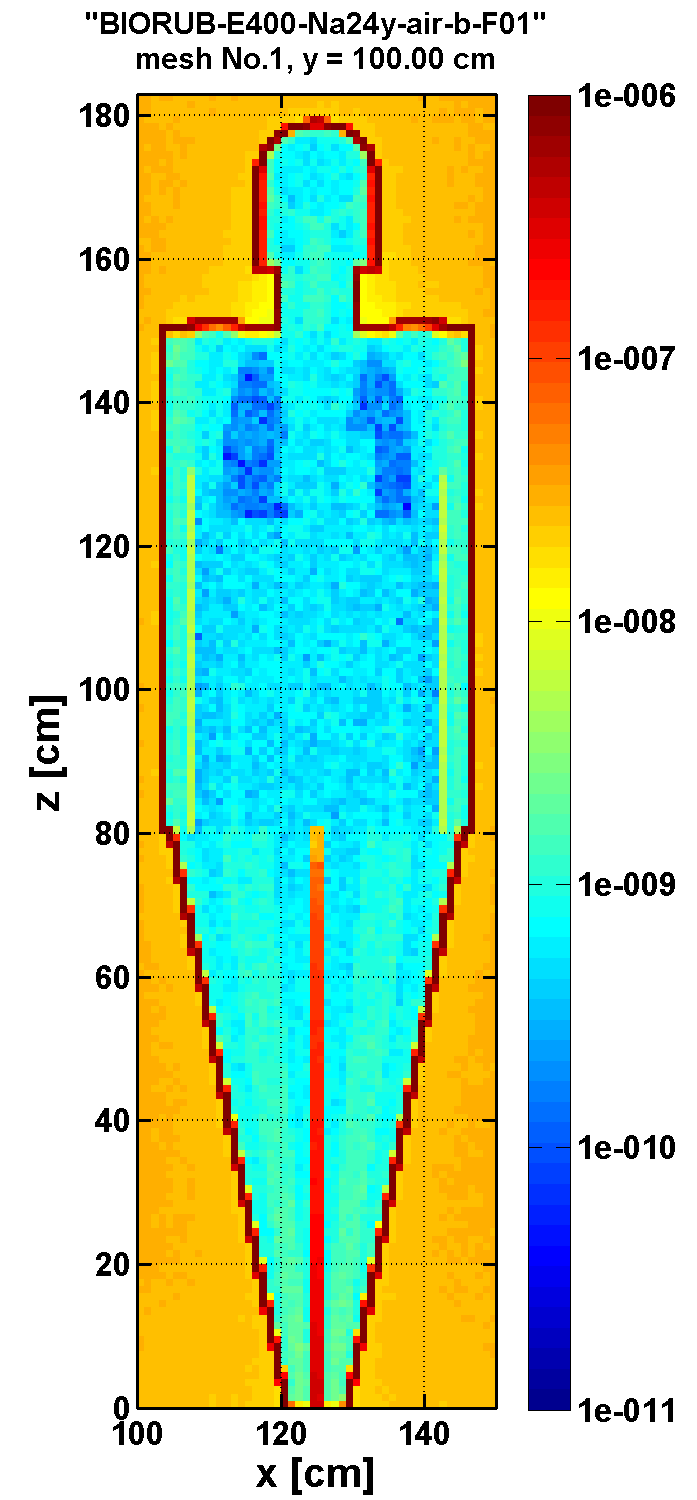

Supplement: Supplementary Materials — The electronic annex presents (1) numerical decrease of the various organs' contribution to the effective dose when protected with individual tested PPE exposed to the other simulated radionuclides dispersed in 10 m3 of the atmosphere in the RAC geometry (Table 2) and (2) visualisations of simulated ORNL phantom energy depositions while only wearing PPE preventing radioactive contamination, and the same PPE together with individual PPE protecting against X- and gamma-ray under it, in a various-dispersed radionuclide's aerosol atmosphere. [file 1641895.f1.zip › 1641895.f1/Electronic annex/Visualization of 2D distributions/Beta contribution/XZ/XZ-b-air-Na24-BIORUB-E400.png]

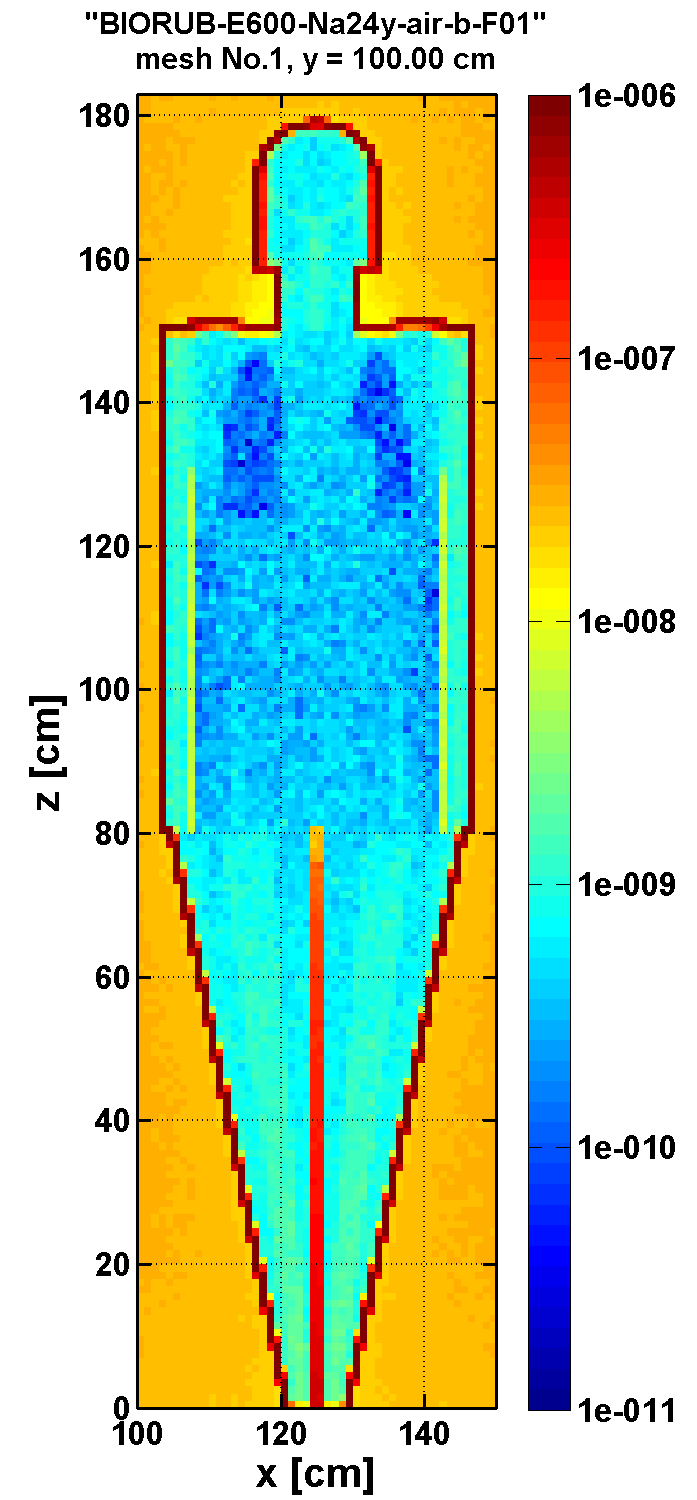

Supplement: Supplementary Materials — The electronic annex presents (1) numerical decrease of the various organs' contribution to the effective dose when protected with individual tested PPE exposed to the other simulated radionuclides dispersed in 10 m3 of the atmosphere in the RAC geometry (Table 2) and (2) visualisations of simulated ORNL phantom energy depositions while only wearing PPE preventing radioactive contamination, and the same PPE together with individual PPE protecting against X- and gamma-ray under it, in a various-dispersed radionuclide's aerosol atmosphere. [file 1641895.f1.zip › 1641895.f1/Electronic annex/Visualization of 2D distributions/Beta contribution/XZ/XZ-b-air-Na24-BIORUB-E600.png]

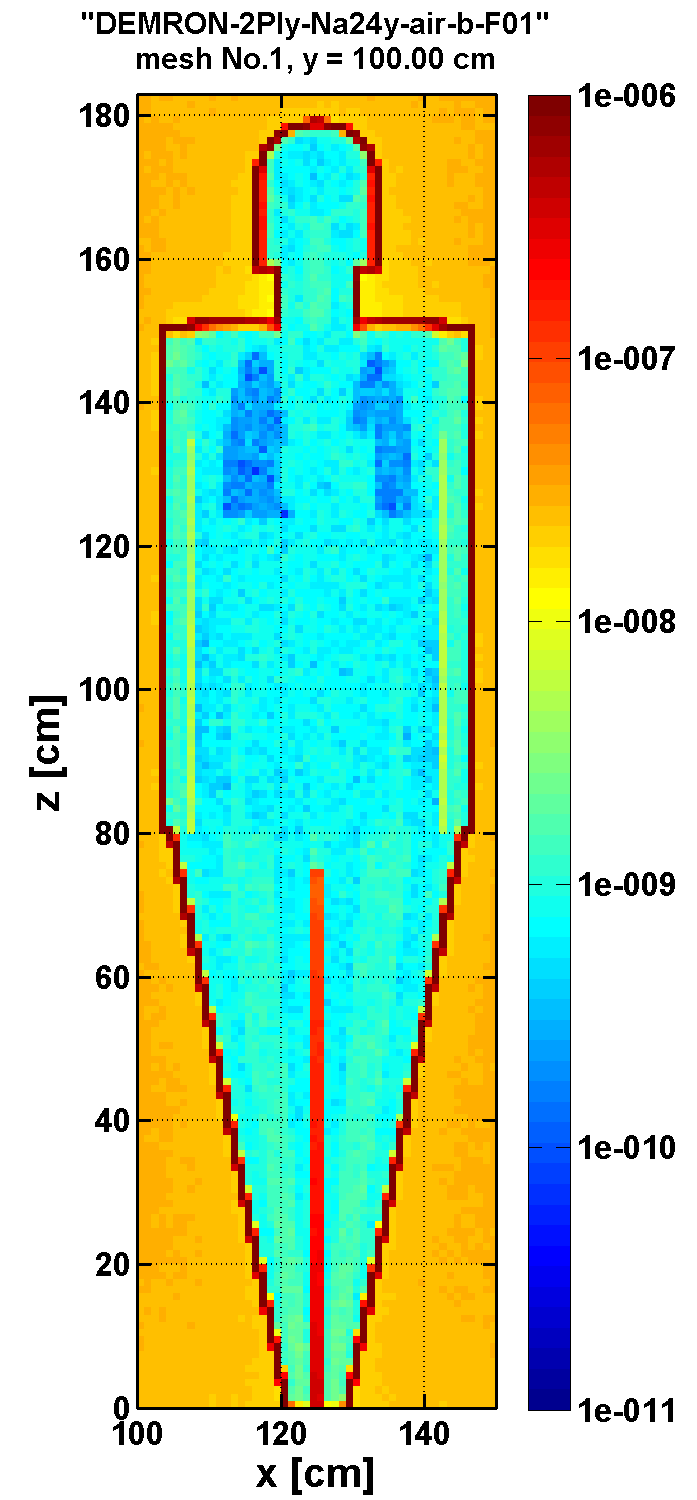

Supplement: Supplementary Materials — The electronic annex presents (1) numerical decrease of the various organs' contribution to the effective dose when protected with individual tested PPE exposed to the other simulated radionuclides dispersed in 10 m3 of the atmosphere in the RAC geometry (Table 2) and (2) visualisations of simulated ORNL phantom energy depositions while only wearing PPE preventing radioactive contamination, and the same PPE together with individual PPE protecting against X- and gamma-ray under it, in a various-dispersed radionuclide's aerosol atmosphere. [file 1641895.f1.zip › 1641895.f1/Electronic annex/Visualization of 2D distributions/Beta contribution/XZ/XZ-b-air-Na24-DEMRON-2Ply.png]

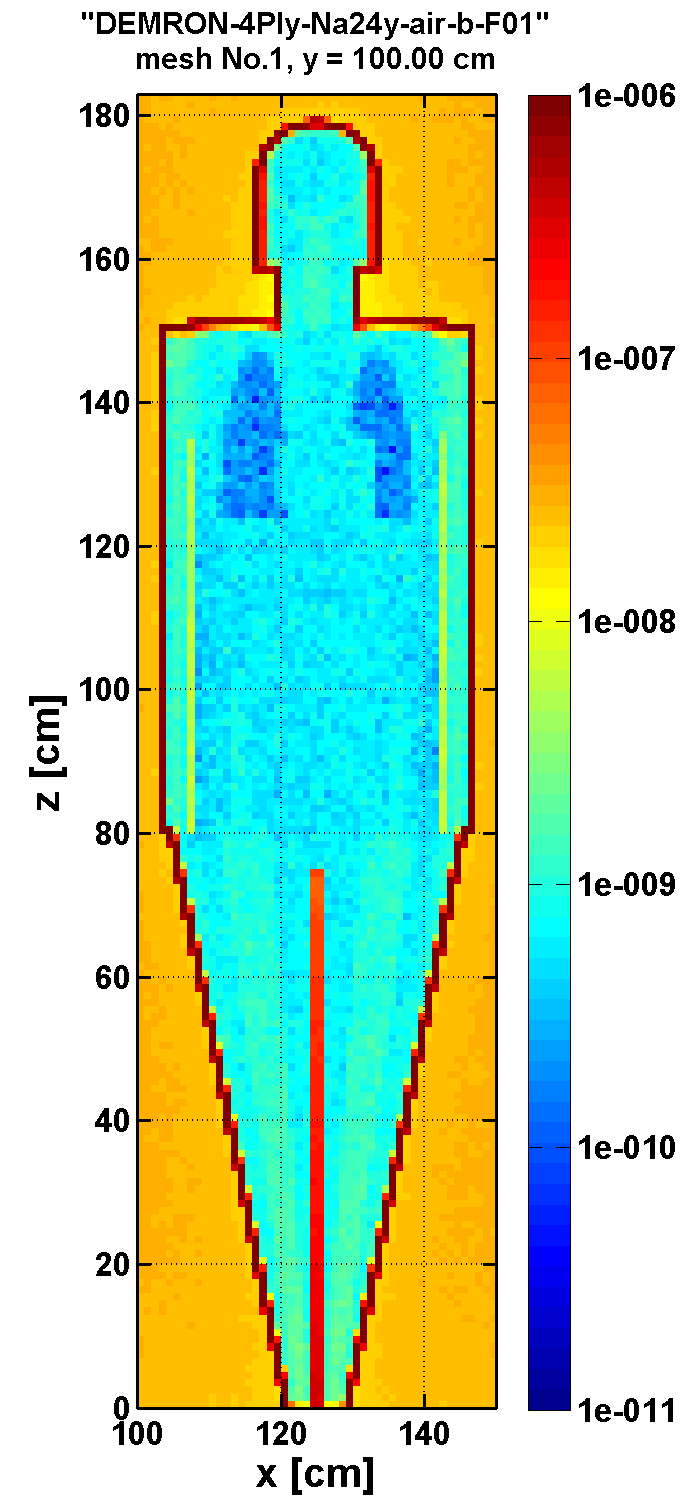

Supplement: Supplementary Materials — The electronic annex presents (1) numerical decrease of the various organs' contribution to the effective dose when protected with individual tested PPE exposed to the other simulated radionuclides dispersed in 10 m3 of the atmosphere in the RAC geometry (Table 2) and (2) visualisations of simulated ORNL phantom energy depositions while only wearing PPE preventing radioactive contamination, and the same PPE together with individual PPE protecting against X- and gamma-ray under it, in a various-dispersed radionuclide's aerosol atmosphere. [file 1641895.f1.zip › 1641895.f1/Electronic annex/Visualization of 2D distributions/Beta contribution/XZ/XZ-b-air-Na24-DEMRON-4Ply.png]

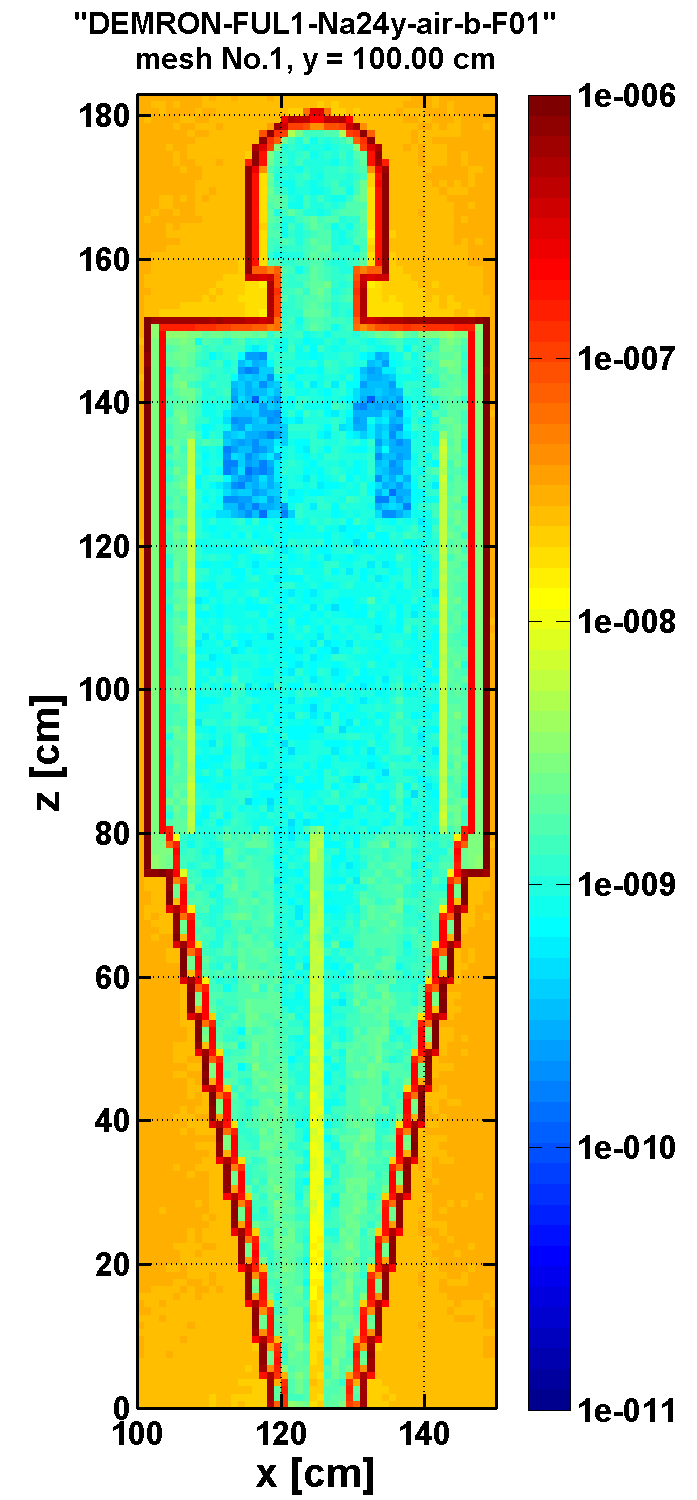

Supplement: Supplementary Materials — The electronic annex presents (1) numerical decrease of the various organs' contribution to the effective dose when protected with individual tested PPE exposed to the other simulated radionuclides dispersed in 10 m3 of the atmosphere in the RAC geometry (Table 2) and (2) visualisations of simulated ORNL phantom energy depositions while only wearing PPE preventing radioactive contamination, and the same PPE together with individual PPE protecting against X- and gamma-ray under it, in a various-dispersed radionuclide's aerosol atmosphere. [file 1641895.f1.zip › 1641895.f1/Electronic annex/Visualization of 2D distributions/Beta contribution/XZ/XZ-b-air-Na24-DEMRON-FUL1.png]

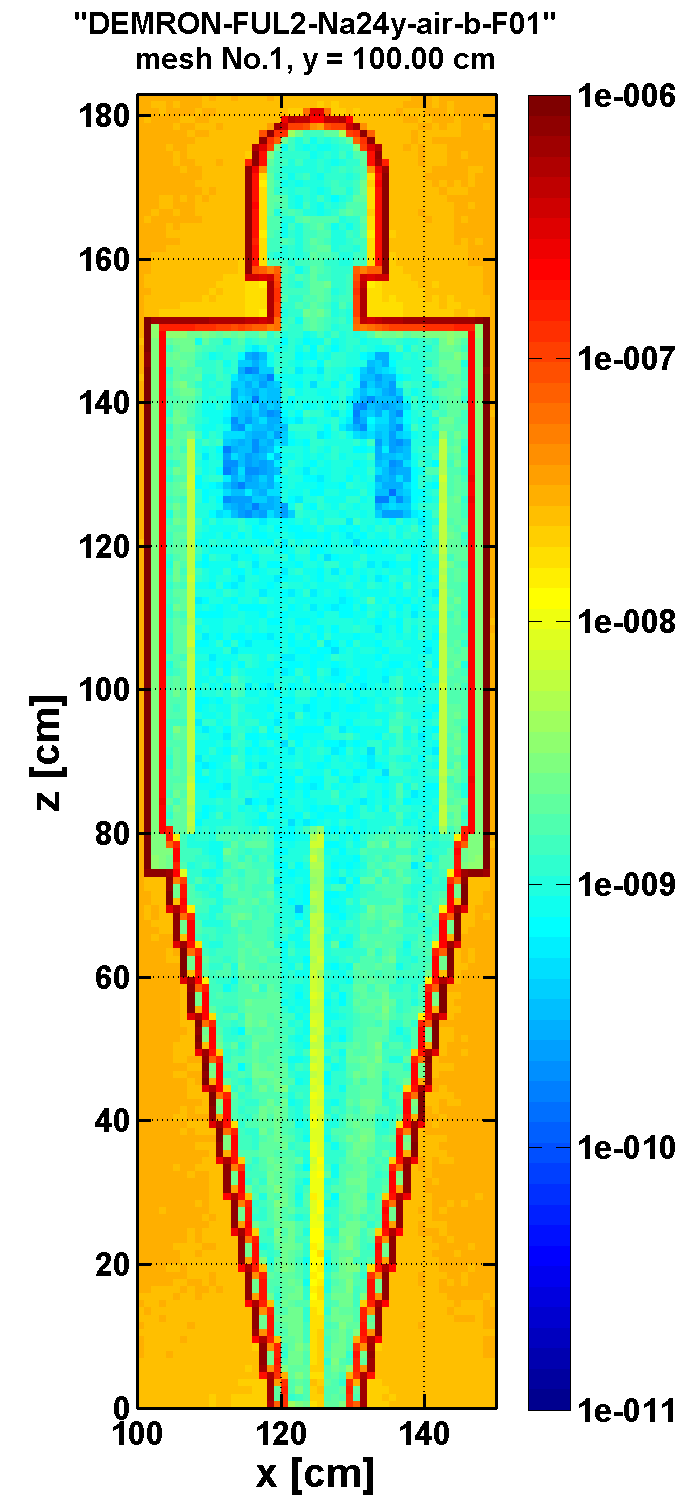

Supplement: Supplementary Materials — The electronic annex presents (1) numerical decrease of the various organs' contribution to the effective dose when protected with individual tested PPE exposed to the other simulated radionuclides dispersed in 10 m3 of the atmosphere in the RAC geometry (Table 2) and (2) visualisations of simulated ORNL phantom energy depositions while only wearing PPE preventing radioactive contamination, and the same PPE together with individual PPE protecting against X- and gamma-ray under it, in a various-dispersed radionuclide's aerosol atmosphere. [file 1641895.f1.zip › 1641895.f1/Electronic annex/Visualization of 2D distributions/Beta contribution/XZ/XZ-b-air-Na24-DEMRON-FUL2.png]

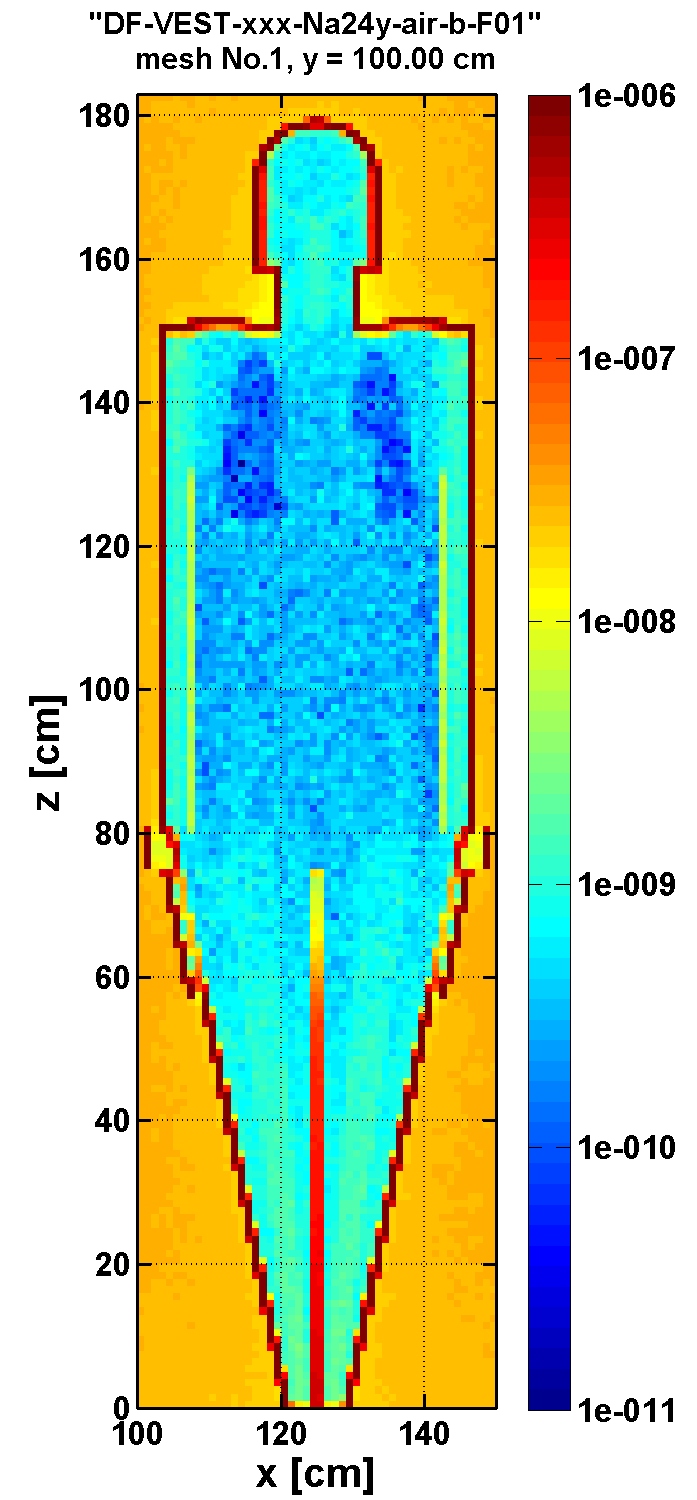

Supplement: Supplementary Materials — The electronic annex presents (1) numerical decrease of the various organs' contribution to the effective dose when protected with individual tested PPE exposed to the other simulated radionuclides dispersed in 10 m3 of the atmosphere in the RAC geometry (Table 2) and (2) visualisations of simulated ORNL phantom energy depositions while only wearing PPE preventing radioactive contamination, and the same PPE together with individual PPE protecting against X- and gamma-ray under it, in a various-dispersed radionuclide's aerosol atmosphere. [file 1641895.f1.zip › 1641895.f1/Electronic annex/Visualization of 2D distributions/Beta contribution/XZ/XZ-b-air-Na24-DF-VEST.png]

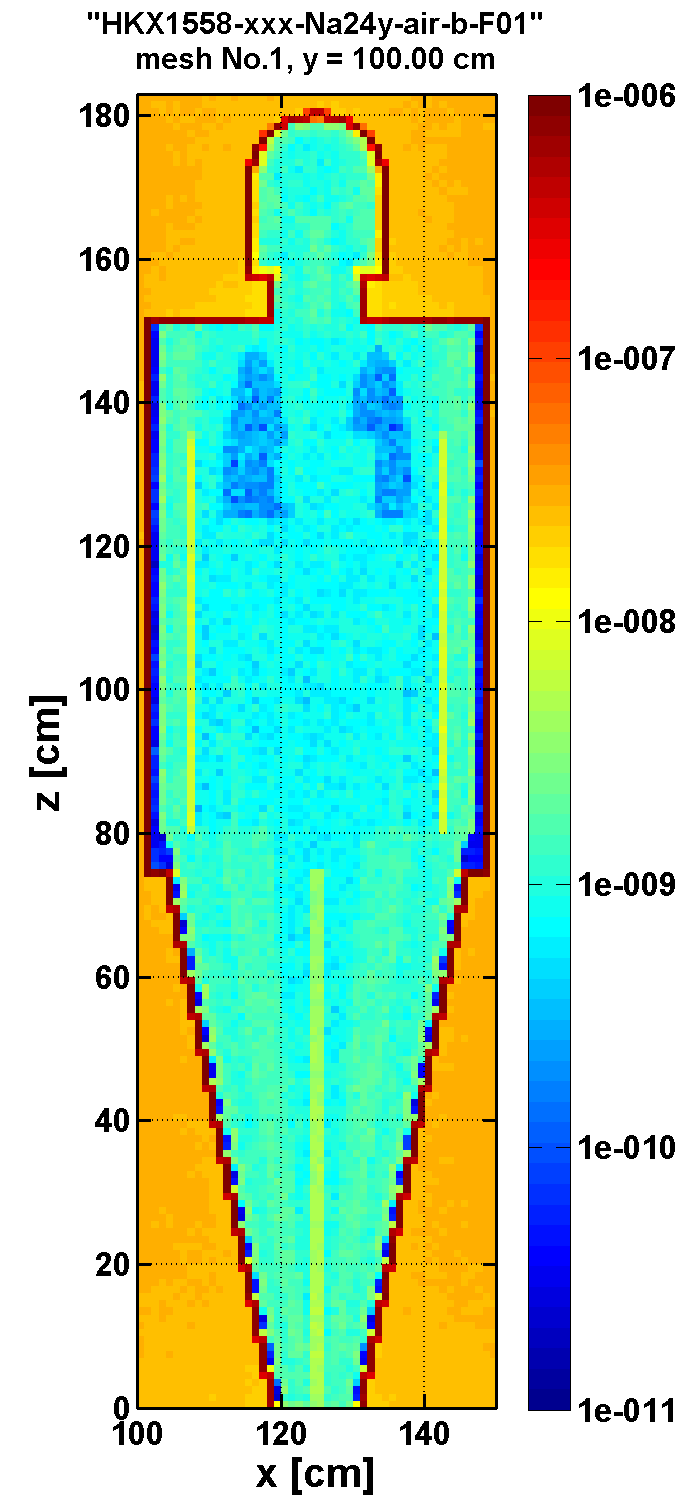

Supplement: Supplementary Materials — The electronic annex presents (1) numerical decrease of the various organs' contribution to the effective dose when protected with individual tested PPE exposed to the other simulated radionuclides dispersed in 10 m3 of the atmosphere in the RAC geometry (Table 2) and (2) visualisations of simulated ORNL phantom energy depositions while only wearing PPE preventing radioactive contamination, and the same PPE together with individual PPE protecting against X- and gamma-ray under it, in a various-dispersed radionuclide's aerosol atmosphere. [file 1641895.f1.zip › 1641895.f1/Electronic annex/Visualization of 2D distributions/Beta contribution/XZ/XZ-b-air-Na24-HKX1558.png]

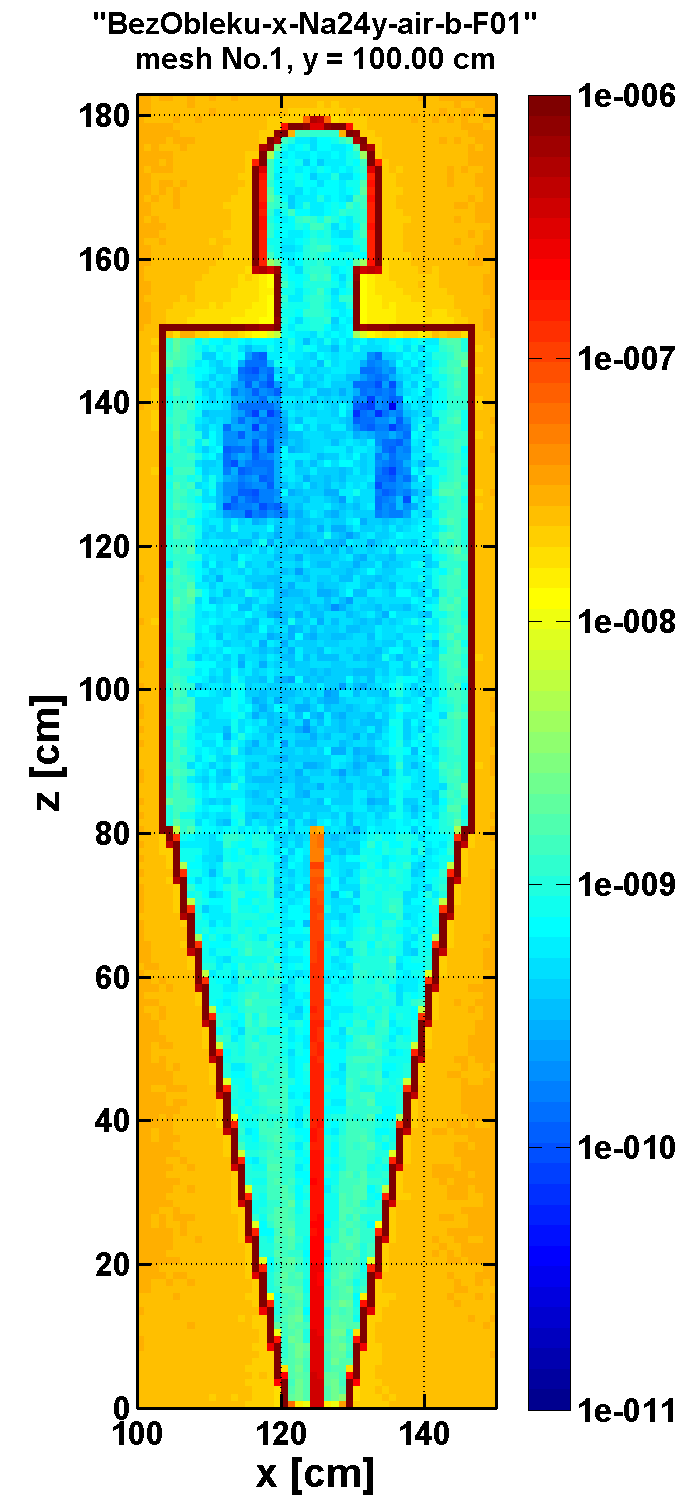

Supplement: Supplementary Materials — The electronic annex presents (1) numerical decrease of the various organs' contribution to the effective dose when protected with individual tested PPE exposed to the other simulated radionuclides dispersed in 10 m3 of the atmosphere in the RAC geometry (Table 2) and (2) visualisations of simulated ORNL phantom energy depositions while only wearing PPE preventing radioactive contamination, and the same PPE together with individual PPE protecting against X- and gamma-ray under it, in a various-dispersed radionuclide's aerosol atmosphere. [file 1641895.f1.zip › 1641895.f1/Electronic annex/Visualization of 2D distributions/Beta contribution/XZ/XZ-b-air-Na24-NoPPE.png]

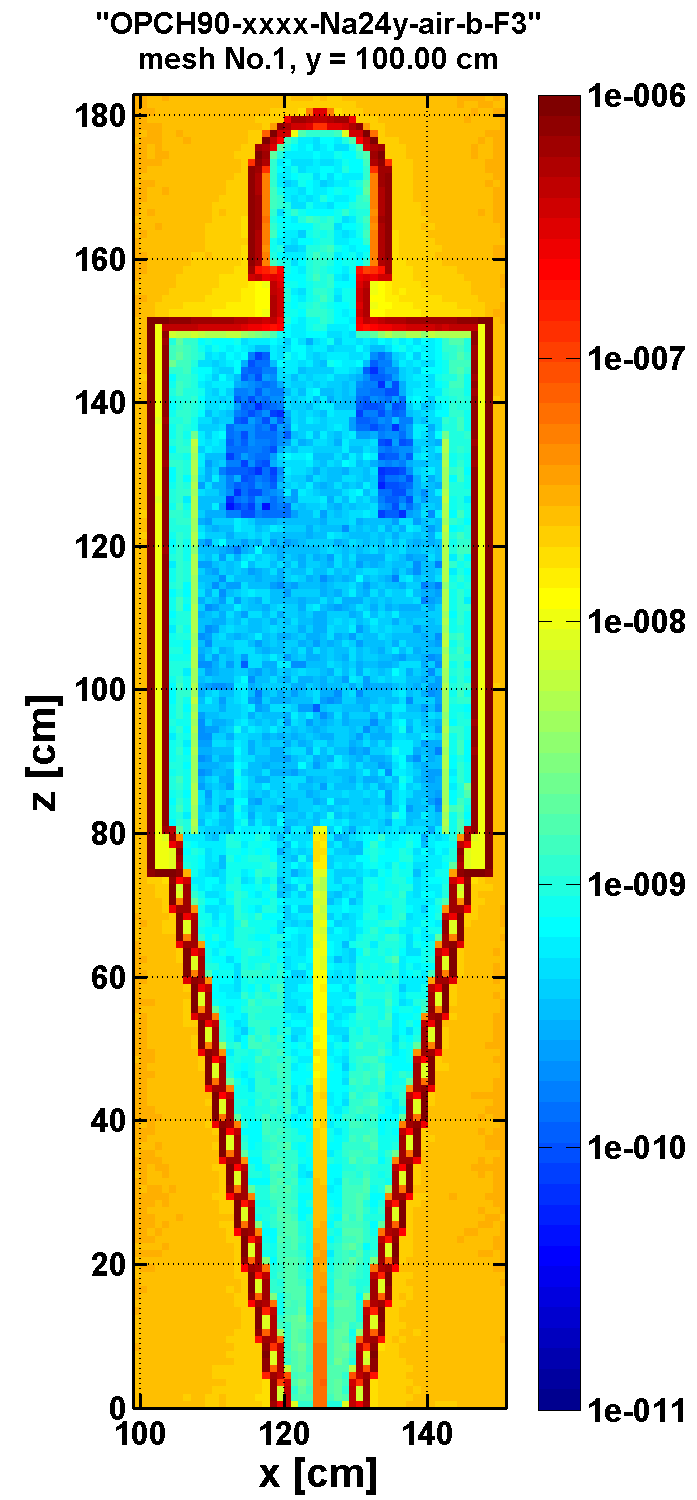

Supplement: Supplementary Materials — The electronic annex presents (1) numerical decrease of the various organs' contribution to the effective dose when protected with individual tested PPE exposed to the other simulated radionuclides dispersed in 10 m3 of the atmosphere in the RAC geometry (Table 2) and (2) visualisations of simulated ORNL phantom energy depositions while only wearing PPE preventing radioactive contamination, and the same PPE together with individual PPE protecting against X- and gamma-ray under it, in a various-dispersed radionuclide's aerosol atmosphere. [file 1641895.f1.zip › 1641895.f1/Electronic annex/Visualization of 2D distributions/Beta contribution/XZ/XZ-b-air-Na24-OPCH90.png]

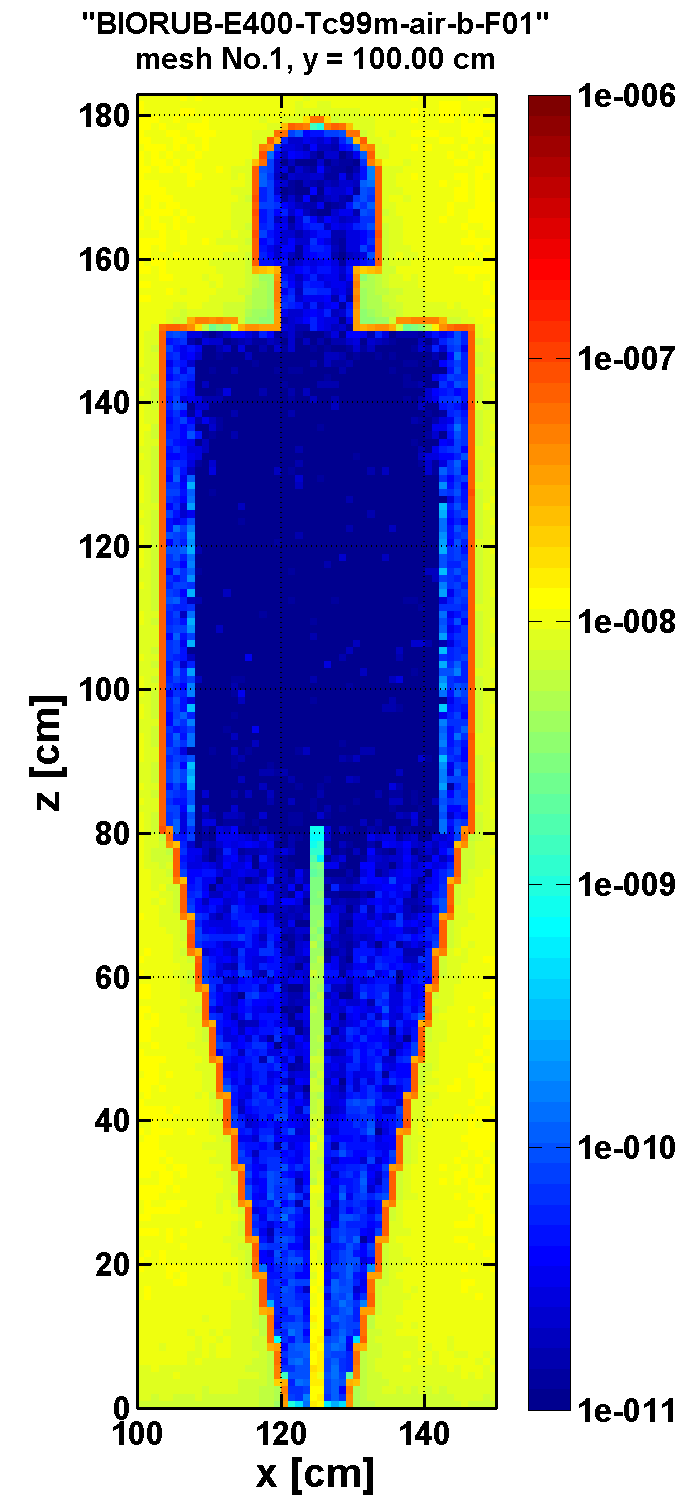

Supplement: Supplementary Materials — The electronic annex presents (1) numerical decrease of the various organs' contribution to the effective dose when protected with individual tested PPE exposed to the other simulated radionuclides dispersed in 10 m3 of the atmosphere in the RAC geometry (Table 2) and (2) visualisations of simulated ORNL phantom energy depositions while only wearing PPE preventing radioactive contamination, and the same PPE together with individual PPE protecting against X- and gamma-ray under it, in a various-dispersed radionuclide's aerosol atmosphere. [file 1641895.f1.zip › 1641895.f1/Electronic annex/Visualization of 2D distributions/Beta contribution/XZ/XZ-b-air-Tc99m-BIORUB-E400.png]

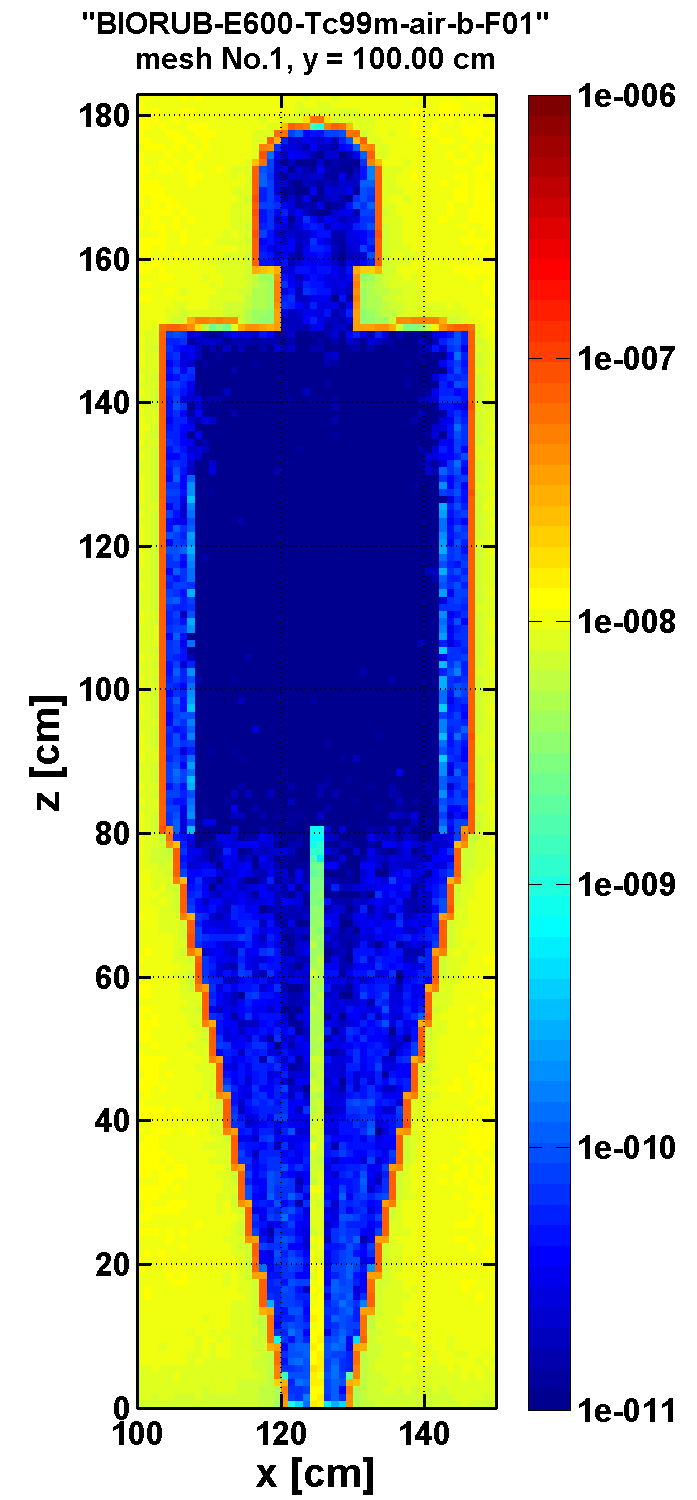

Supplement: Supplementary Materials — The electronic annex presents (1) numerical decrease of the various organs' contribution to the effective dose when protected with individual tested PPE exposed to the other simulated radionuclides dispersed in 10 m3 of the atmosphere in the RAC geometry (Table 2) and (2) visualisations of simulated ORNL phantom energy depositions while only wearing PPE preventing radioactive contamination, and the same PPE together with individual PPE protecting against X- and gamma-ray under it, in a various-dispersed radionuclide's aerosol atmosphere. [file 1641895.f1.zip › 1641895.f1/Electronic annex/Visualization of 2D distributions/Beta contribution/XZ/XZ-b-air-Tc99m-BIORUB-E600.png]

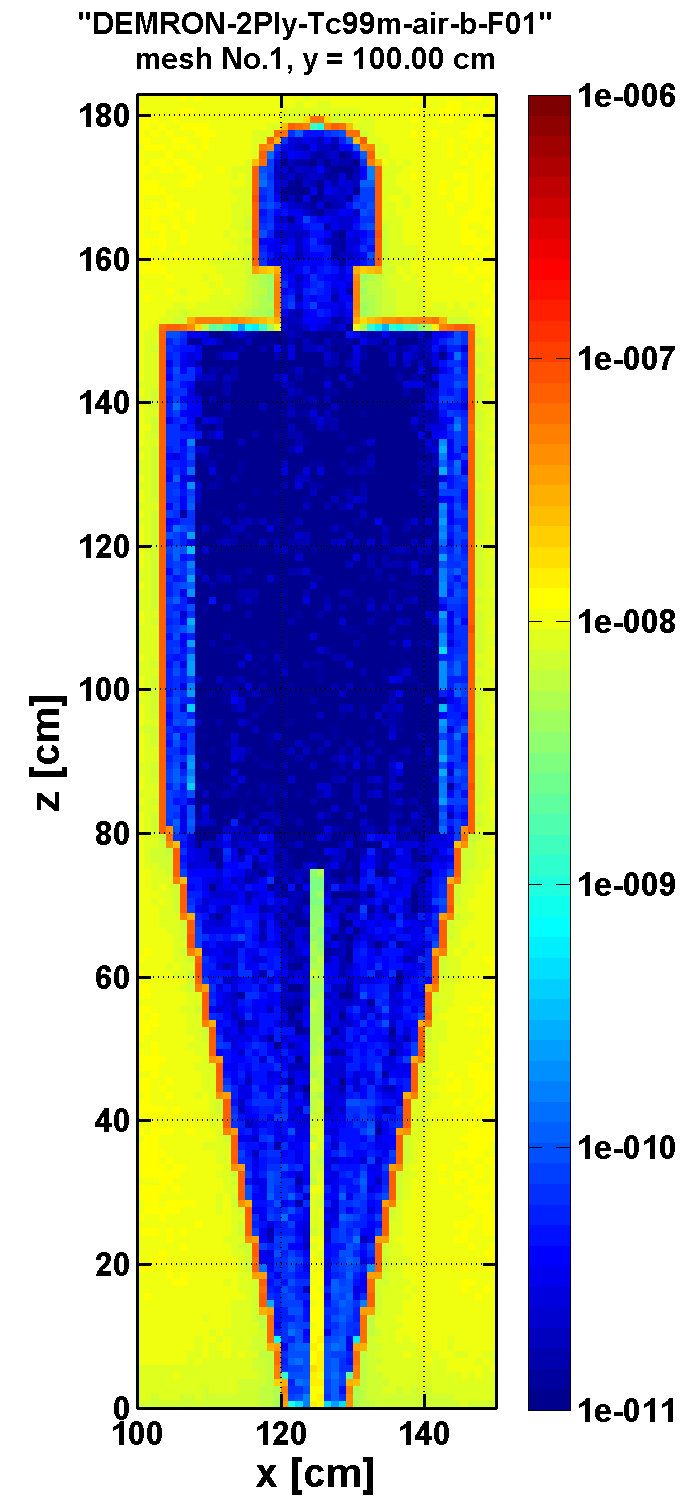

Supplement: Supplementary Materials — The electronic annex presents (1) numerical decrease of the various organs' contribution to the effective dose when protected with individual tested PPE exposed to the other simulated radionuclides dispersed in 10 m3 of the atmosphere in the RAC geometry (Table 2) and (2) visualisations of simulated ORNL phantom energy depositions while only wearing PPE preventing radioactive contamination, and the same PPE together with individual PPE protecting against X- and gamma-ray under it, in a various-dispersed radionuclide's aerosol atmosphere. [file 1641895.f1.zip › 1641895.f1/Electronic annex/Visualization of 2D distributions/Beta contribution/XZ/XZ-b-air-Tc99m-DEMRON-2Ply.png]

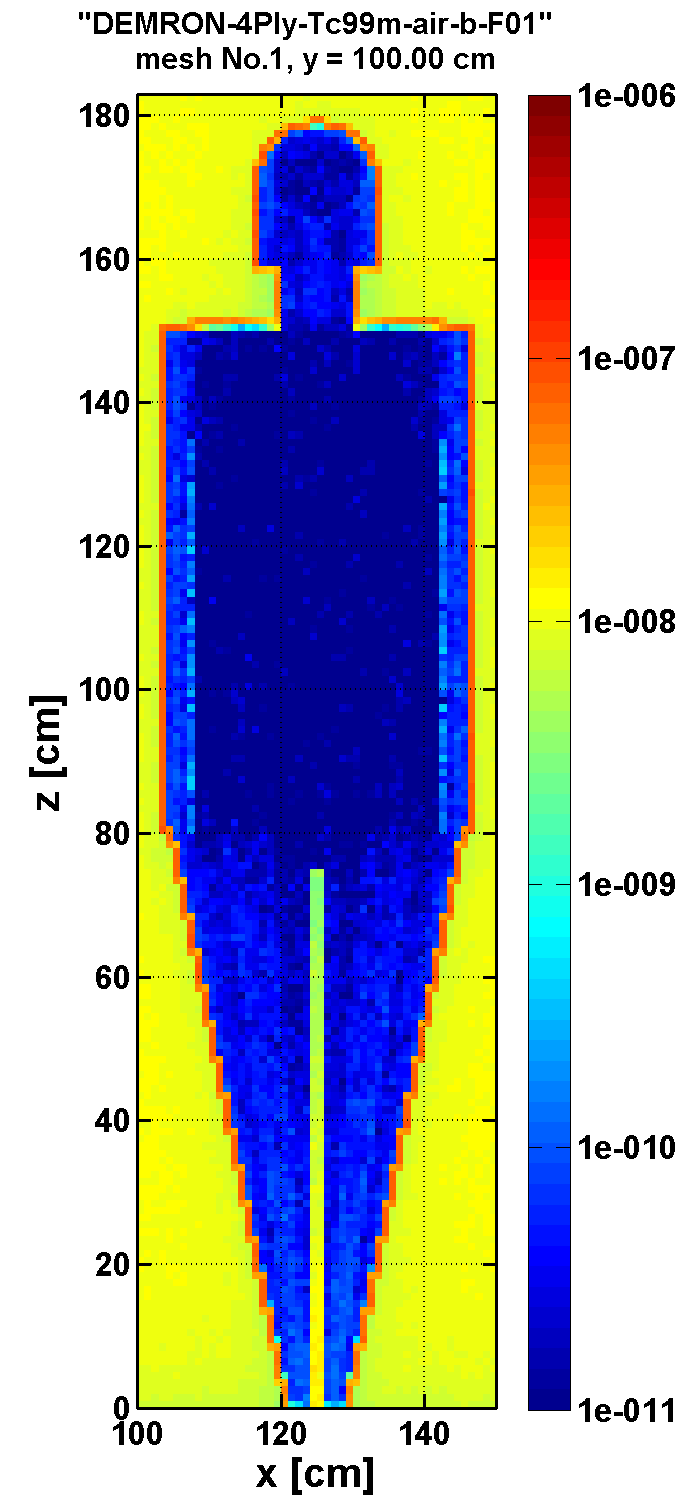

Supplement: Supplementary Materials — The electronic annex presents (1) numerical decrease of the various organs' contribution to the effective dose when protected with individual tested PPE exposed to the other simulated radionuclides dispersed in 10 m3 of the atmosphere in the RAC geometry (Table 2) and (2) visualisations of simulated ORNL phantom energy depositions while only wearing PPE preventing radioactive contamination, and the same PPE together with individual PPE protecting against X- and gamma-ray under it, in a various-dispersed radionuclide's aerosol atmosphere. [file 1641895.f1.zip › 1641895.f1/Electronic annex/Visualization of 2D distributions/Beta contribution/XZ/XZ-b-air-Tc99m-DEMRON-4Ply.png]

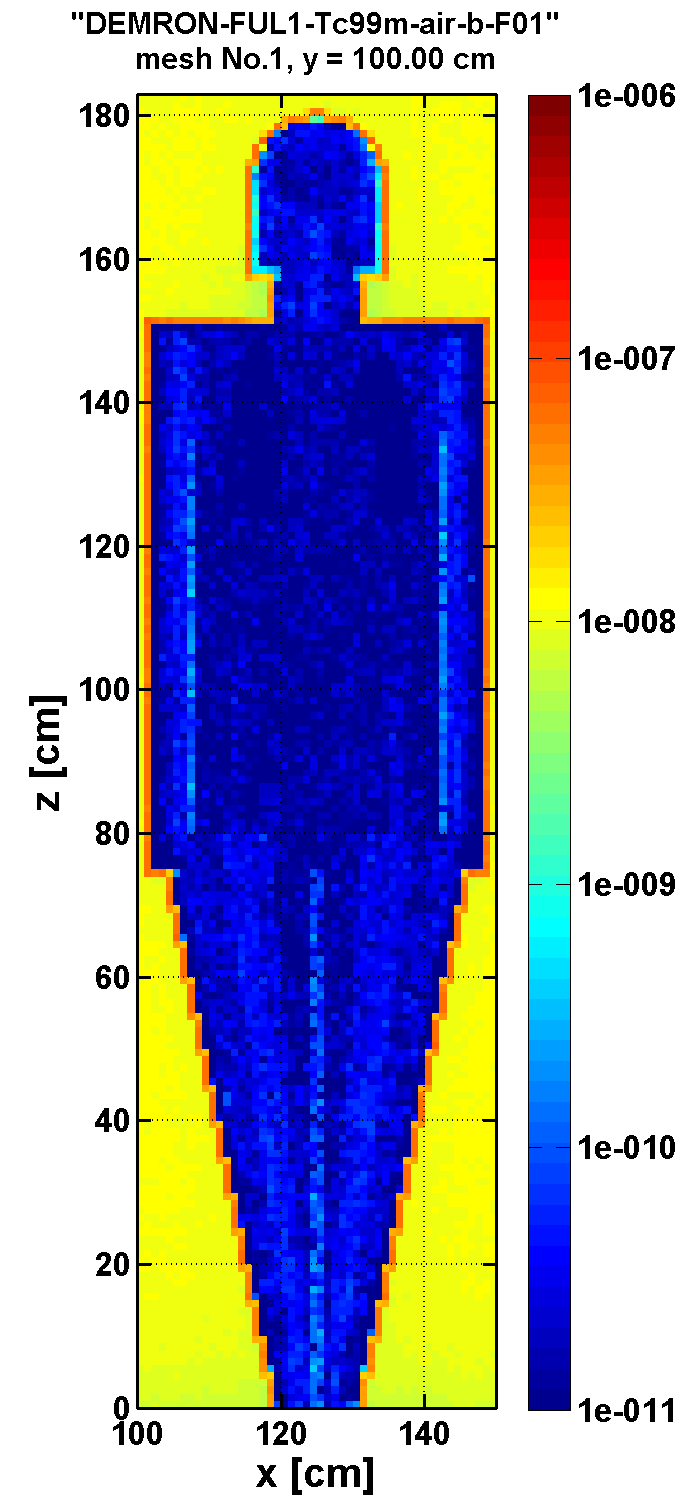

Supplement: Supplementary Materials — The electronic annex presents (1) numerical decrease of the various organs' contribution to the effective dose when protected with individual tested PPE exposed to the other simulated radionuclides dispersed in 10 m3 of the atmosphere in the RAC geometry (Table 2) and (2) visualisations of simulated ORNL phantom energy depositions while only wearing PPE preventing radioactive contamination, and the same PPE together with individual PPE protecting against X- and gamma-ray under it, in a various-dispersed radionuclide's aerosol atmosphere. [file 1641895.f1.zip › 1641895.f1/Electronic annex/Visualization of 2D distributions/Beta contribution/XZ/XZ-b-air-Tc99m-DEMRON-FUL1.png]

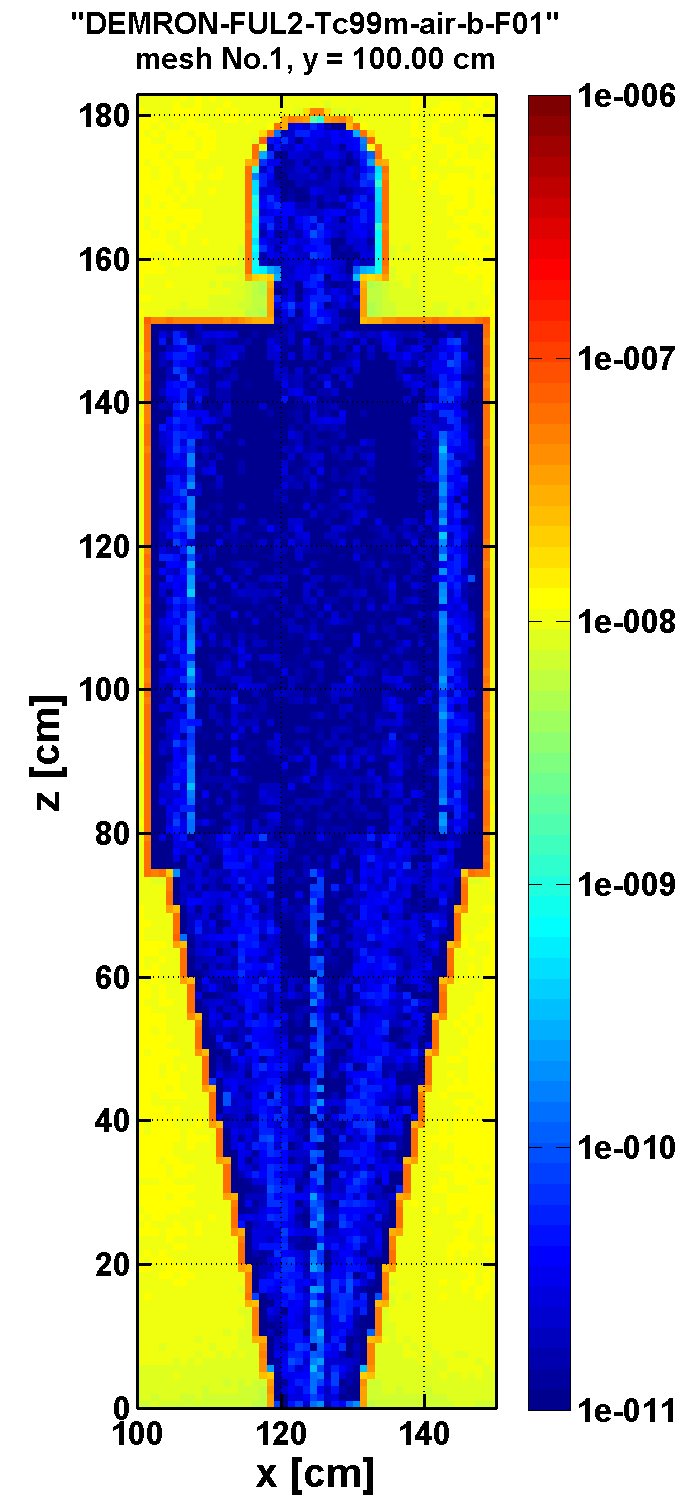

Supplement: Supplementary Materials — The electronic annex presents (1) numerical decrease of the various organs' contribution to the effective dose when protected with individual tested PPE exposed to the other simulated radionuclides dispersed in 10 m3 of the atmosphere in the RAC geometry (Table 2) and (2) visualisations of simulated ORNL phantom energy depositions while only wearing PPE preventing radioactive contamination, and the same PPE together with individual PPE protecting against X- and gamma-ray under it, in a various-dispersed radionuclide's aerosol atmosphere. [file 1641895.f1.zip › 1641895.f1/Electronic annex/Visualization of 2D distributions/Beta contribution/XZ/XZ-b-air-Tc99m-DEMRON-FUL2.png]

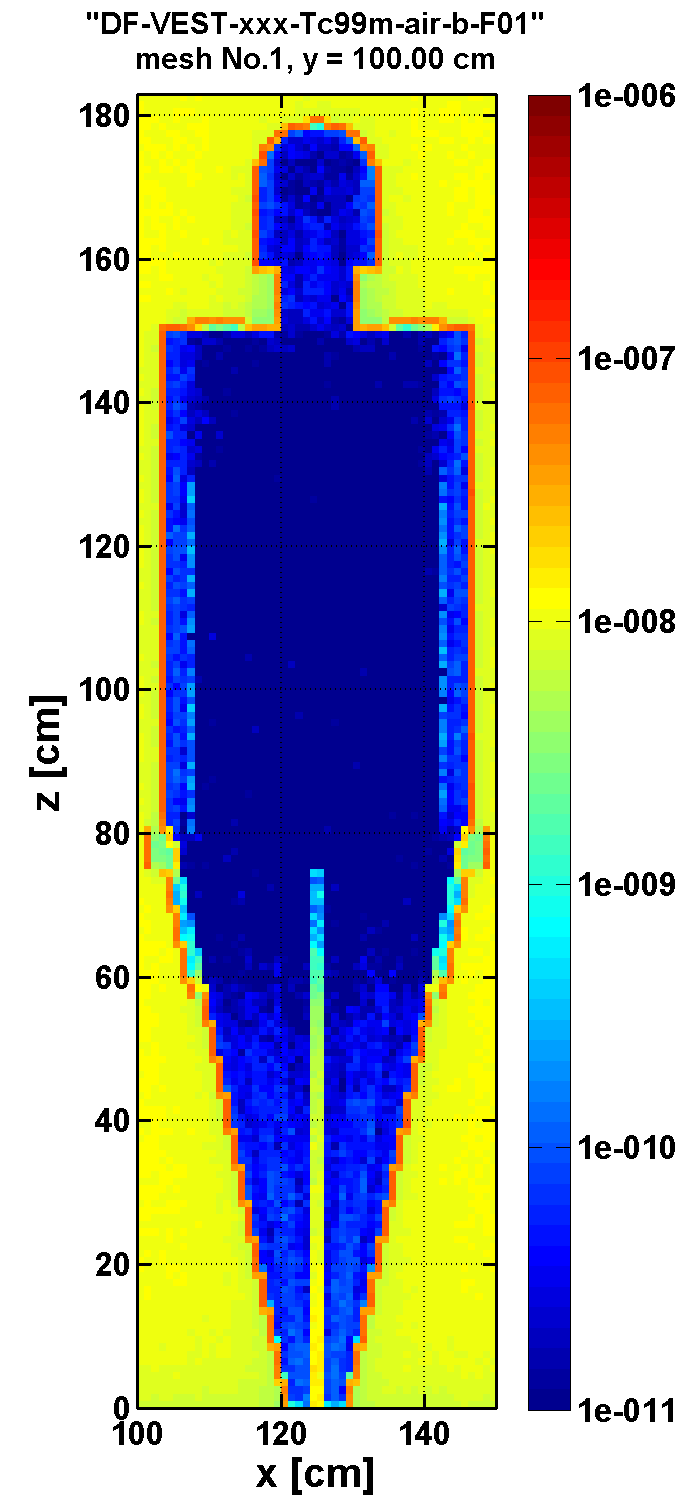

Supplement: Supplementary Materials — The electronic annex presents (1) numerical decrease of the various organs' contribution to the effective dose when protected with individual tested PPE exposed to the other simulated radionuclides dispersed in 10 m3 of the atmosphere in the RAC geometry (Table 2) and (2) visualisations of simulated ORNL phantom energy depositions while only wearing PPE preventing radioactive contamination, and the same PPE together with individual PPE protecting against X- and gamma-ray under it, in a various-dispersed radionuclide's aerosol atmosphere. [file 1641895.f1.zip › 1641895.f1/Electronic annex/Visualization of 2D distributions/Beta contribution/XZ/XZ-b-air-Tc99m-DF-VEST.png]

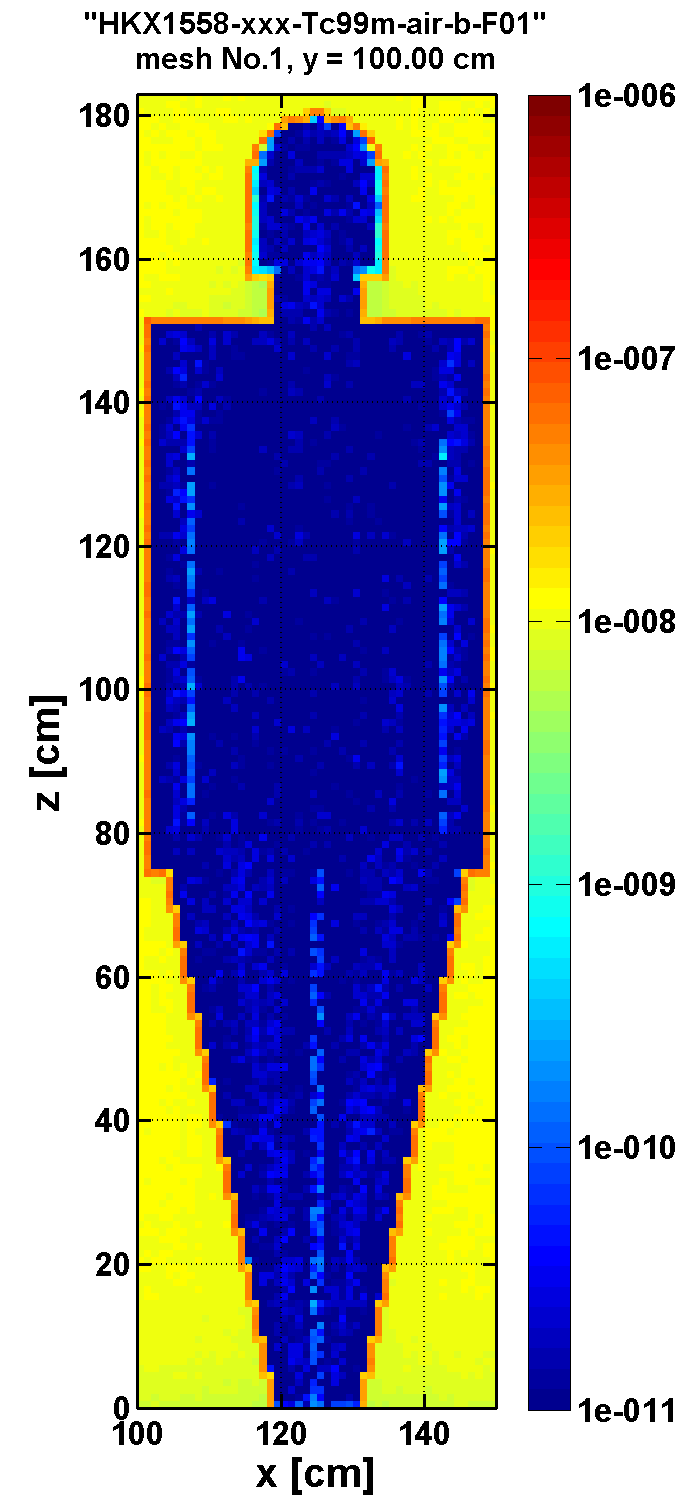

Supplement: Supplementary Materials — The electronic annex presents (1) numerical decrease of the various organs' contribution to the effective dose when protected with individual tested PPE exposed to the other simulated radionuclides dispersed in 10 m3 of the atmosphere in the RAC geometry (Table 2) and (2) visualisations of simulated ORNL phantom energy depositions while only wearing PPE preventing radioactive contamination, and the same PPE together with individual PPE protecting against X- and gamma-ray under it, in a various-dispersed radionuclide's aerosol atmosphere. [file 1641895.f1.zip › 1641895.f1/Electronic annex/Visualization of 2D distributions/Beta contribution/XZ/XZ-b-air-Tc99m-HKX1558.png]

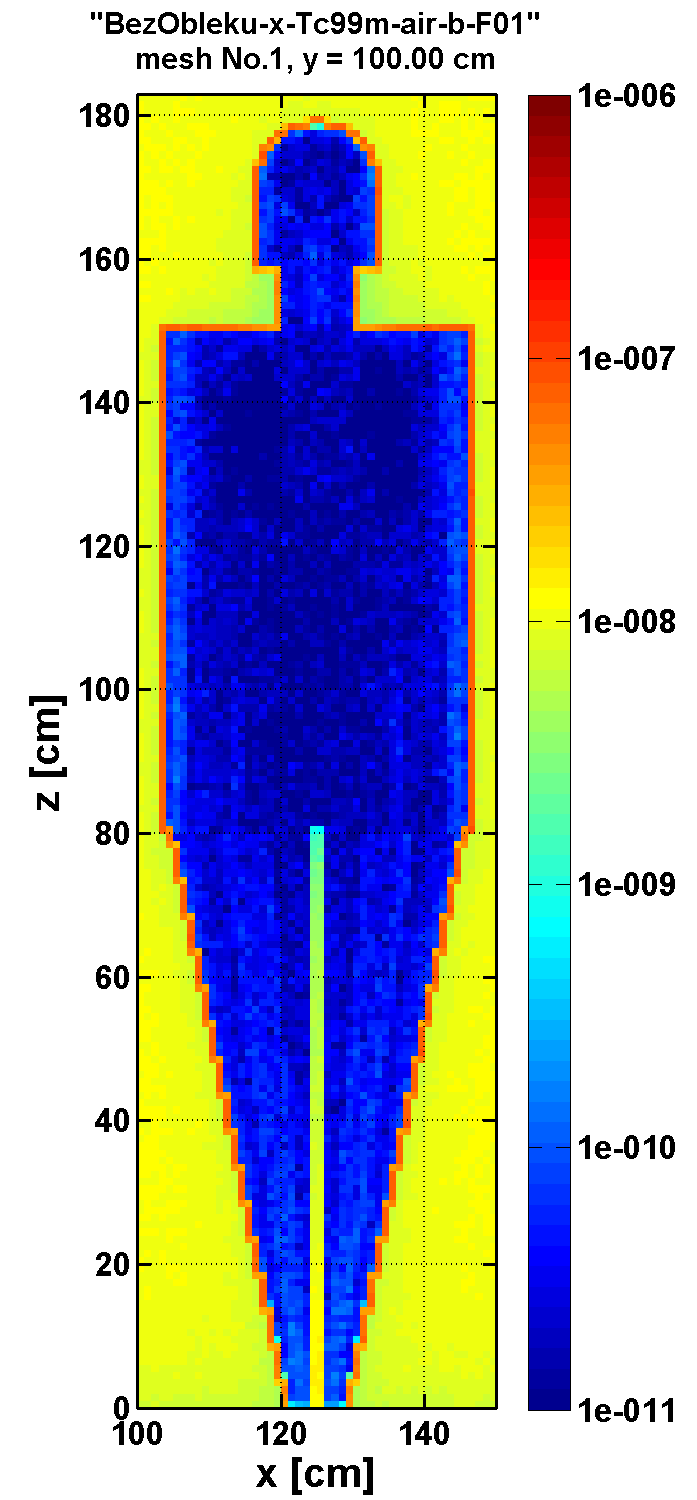

Supplement: Supplementary Materials — The electronic annex presents (1) numerical decrease of the various organs' contribution to the effective dose when protected with individual tested PPE exposed to the other simulated radionuclides dispersed in 10 m3 of the atmosphere in the RAC geometry (Table 2) and (2) visualisations of simulated ORNL phantom energy depositions while only wearing PPE preventing radioactive contamination, and the same PPE together with individual PPE protecting against X- and gamma-ray under it, in a various-dispersed radionuclide's aerosol atmosphere. [file 1641895.f1.zip › 1641895.f1/Electronic annex/Visualization of 2D distributions/Beta contribution/XZ/XZ-b-air-Tc99m-NoPPE.png]

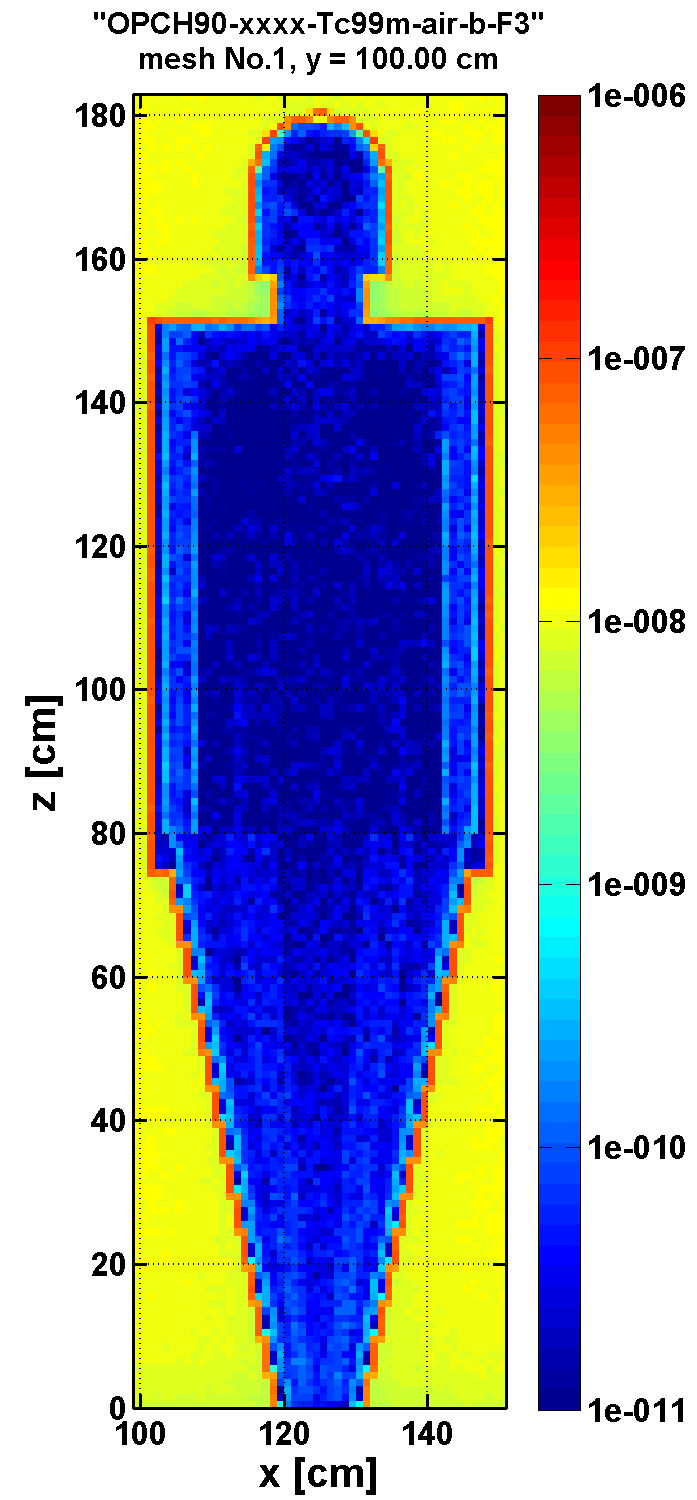

Supplement: Supplementary Materials — The electronic annex presents (1) numerical decrease of the various organs' contribution to the effective dose when protected with individual tested PPE exposed to the other simulated radionuclides dispersed in 10 m3 of the atmosphere in the RAC geometry (Table 2) and (2) visualisations of simulated ORNL phantom energy depositions while only wearing PPE preventing radioactive contamination, and the same PPE together with individual PPE protecting against X- and gamma-ray under it, in a various-dispersed radionuclide's aerosol atmosphere. [file 1641895.f1.zip › 1641895.f1/Electronic annex/Visualization of 2D distributions/Beta contribution/XZ/XZ-b-air-Tc99m-OPCH90.png]

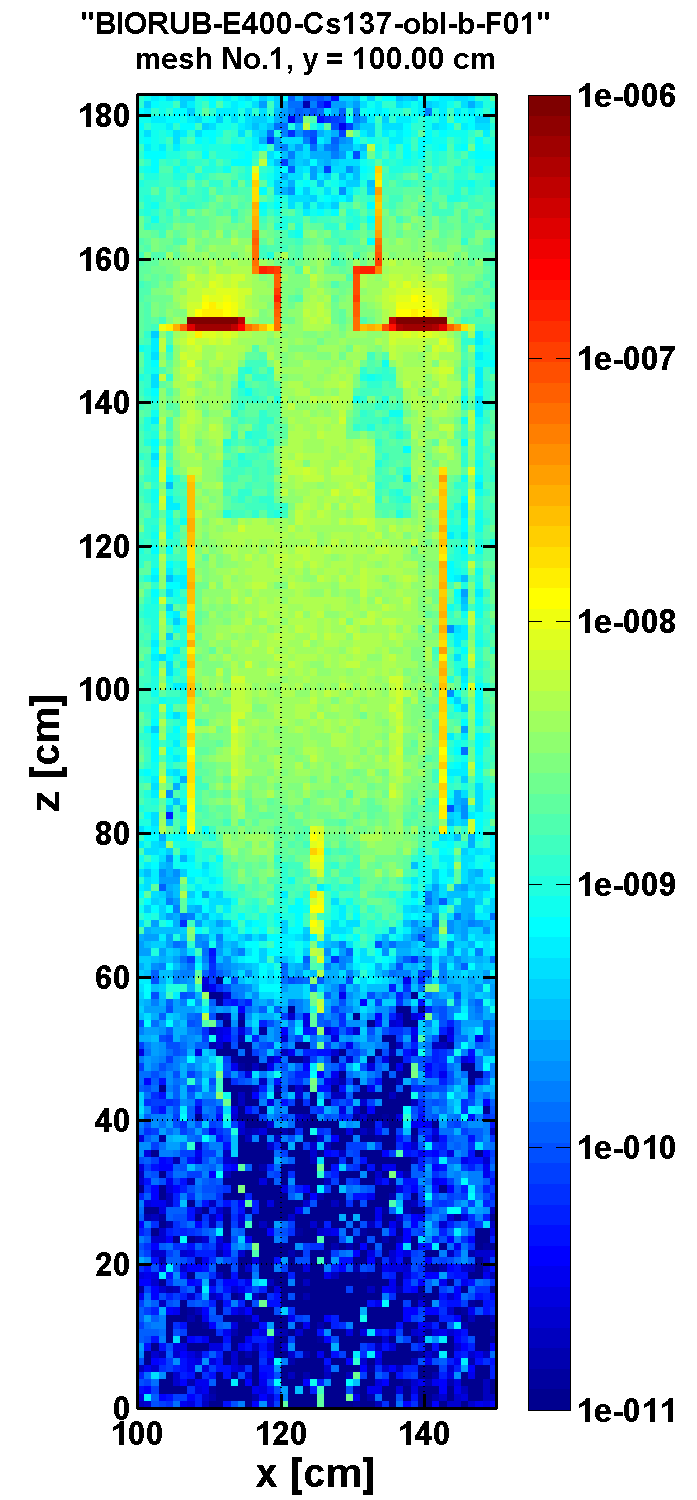

Supplement: Supplementary Materials — The electronic annex presents (1) numerical decrease of the various organs' contribution to the effective dose when protected with individual tested PPE exposed to the other simulated radionuclides dispersed in 10 m3 of the atmosphere in the RAC geometry (Table 2) and (2) visualisations of simulated ORNL phantom energy depositions while only wearing PPE preventing radioactive contamination, and the same PPE together with individual PPE protecting against X- and gamma-ray under it, in a various-dispersed radionuclide's aerosol atmosphere. [file 1641895.f1.zip › 1641895.f1/Electronic annex/Visualization of 2D distributions/Beta contribution/XZ/XZ-b-ppe-Cs137-BIORUB-E400.png]

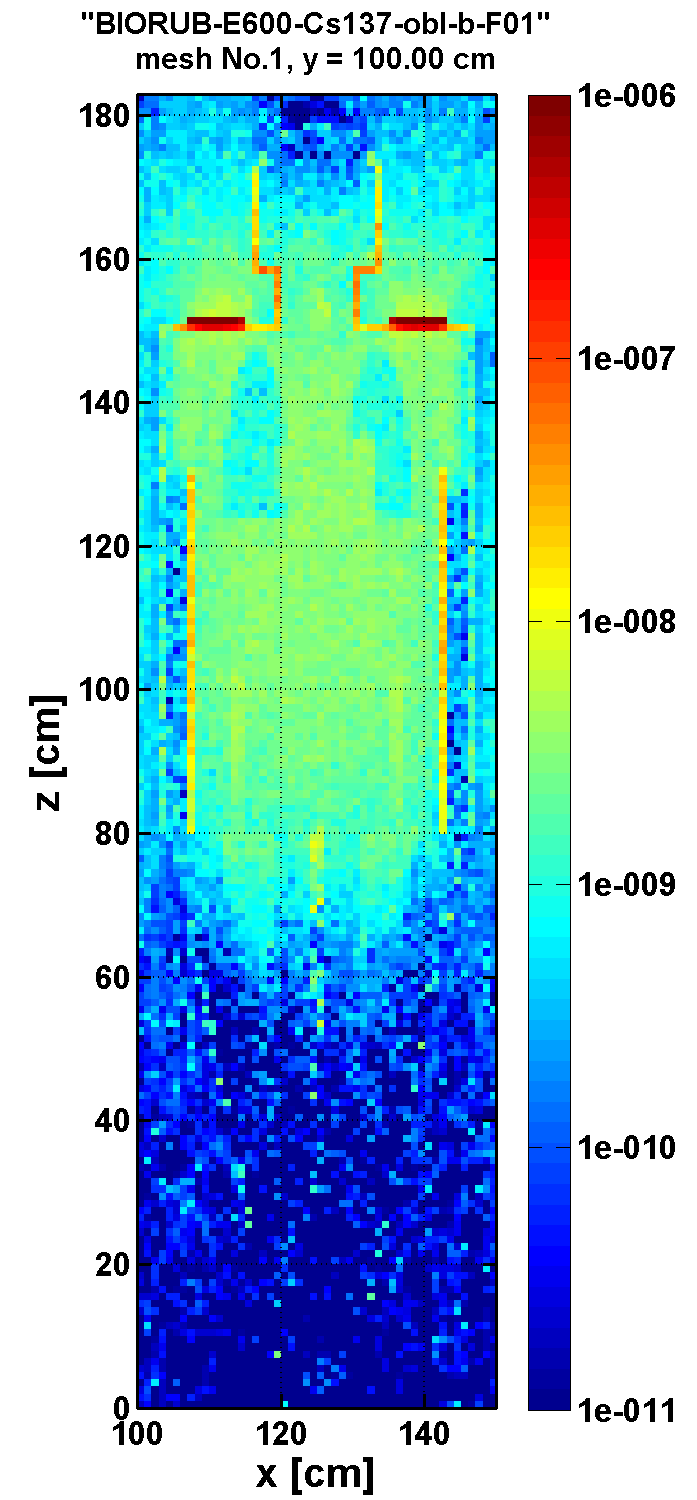

Supplement: Supplementary Materials — The electronic annex presents (1) numerical decrease of the various organs' contribution to the effective dose when protected with individual tested PPE exposed to the other simulated radionuclides dispersed in 10 m3 of the atmosphere in the RAC geometry (Table 2) and (2) visualisations of simulated ORNL phantom energy depositions while only wearing PPE preventing radioactive contamination, and the same PPE together with individual PPE protecting against X- and gamma-ray under it, in a various-dispersed radionuclide's aerosol atmosphere. [file 1641895.f1.zip › 1641895.f1/Electronic annex/Visualization of 2D distributions/Beta contribution/XZ/XZ-b-ppe-Cs137-BIORUB-E600.png]

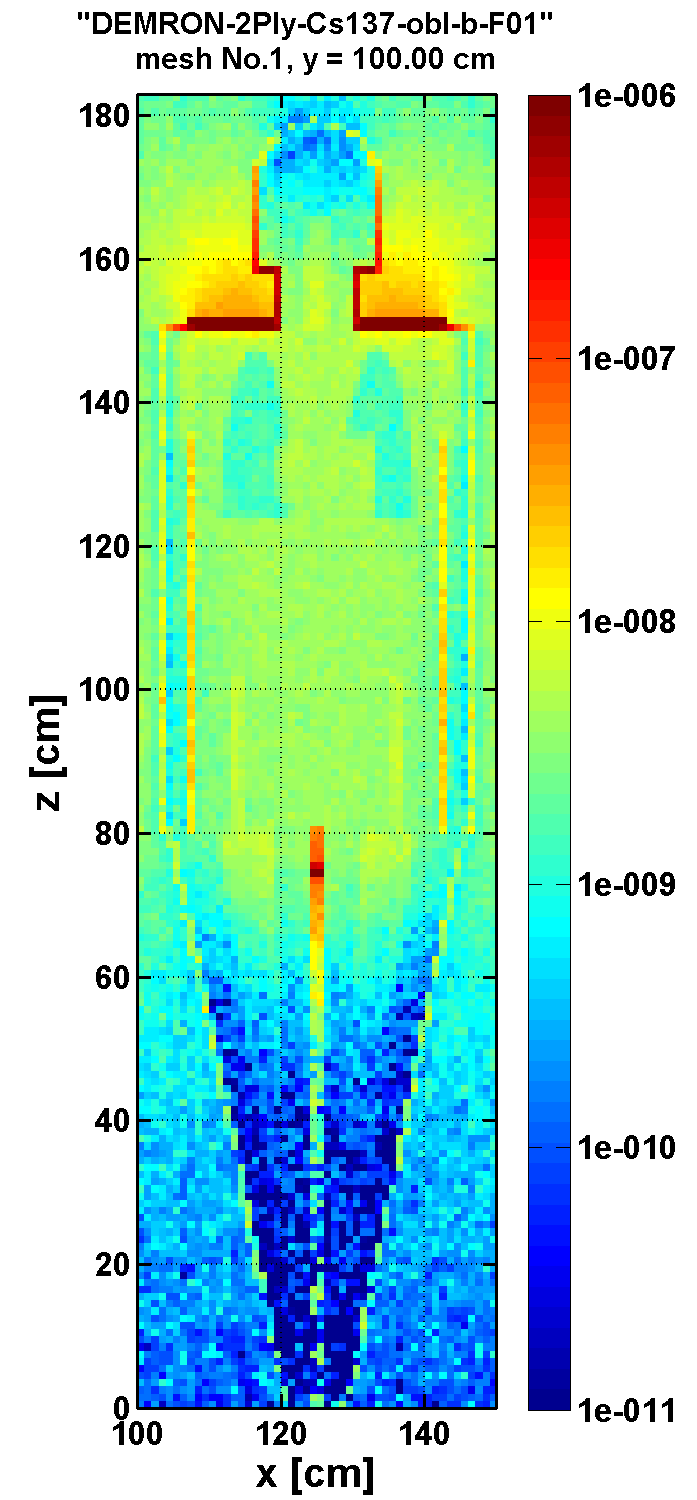

Supplement: Supplementary Materials — The electronic annex presents (1) numerical decrease of the various organs' contribution to the effective dose when protected with individual tested PPE exposed to the other simulated radionuclides dispersed in 10 m3 of the atmosphere in the RAC geometry (Table 2) and (2) visualisations of simulated ORNL phantom energy depositions while only wearing PPE preventing radioactive contamination, and the same PPE together with individual PPE protecting against X- and gamma-ray under it, in a various-dispersed radionuclide's aerosol atmosphere. [file 1641895.f1.zip › 1641895.f1/Electronic annex/Visualization of 2D distributions/Beta contribution/XZ/XZ-b-ppe-Cs137-DEMRON-2Ply.png]

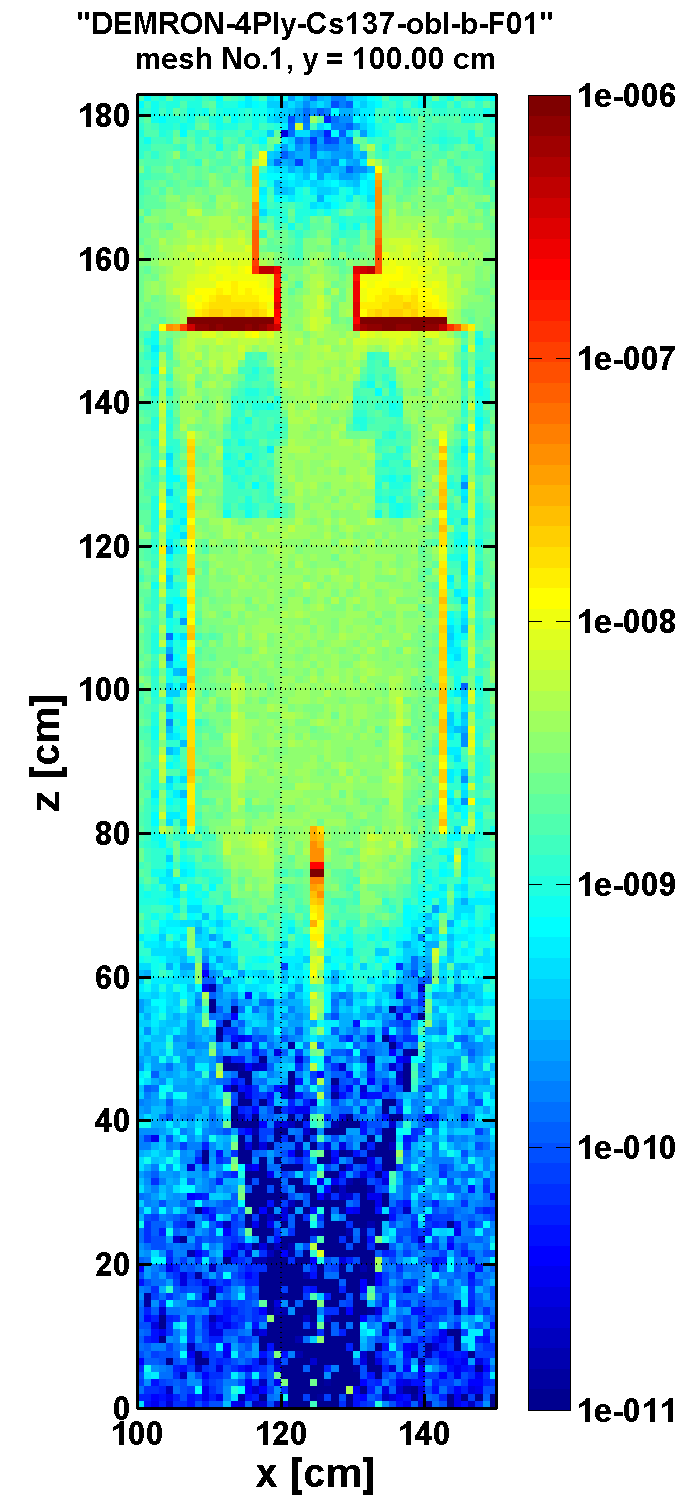

Supplement: Supplementary Materials — The electronic annex presents (1) numerical decrease of the various organs' contribution to the effective dose when protected with individual tested PPE exposed to the other simulated radionuclides dispersed in 10 m3 of the atmosphere in the RAC geometry (Table 2) and (2) visualisations of simulated ORNL phantom energy depositions while only wearing PPE preventing radioactive contamination, and the same PPE together with individual PPE protecting against X- and gamma-ray under it, in a various-dispersed radionuclide's aerosol atmosphere. [file 1641895.f1.zip › 1641895.f1/Electronic annex/Visualization of 2D distributions/Beta contribution/XZ/XZ-b-ppe-Cs137-DEMRON-4Ply.png]

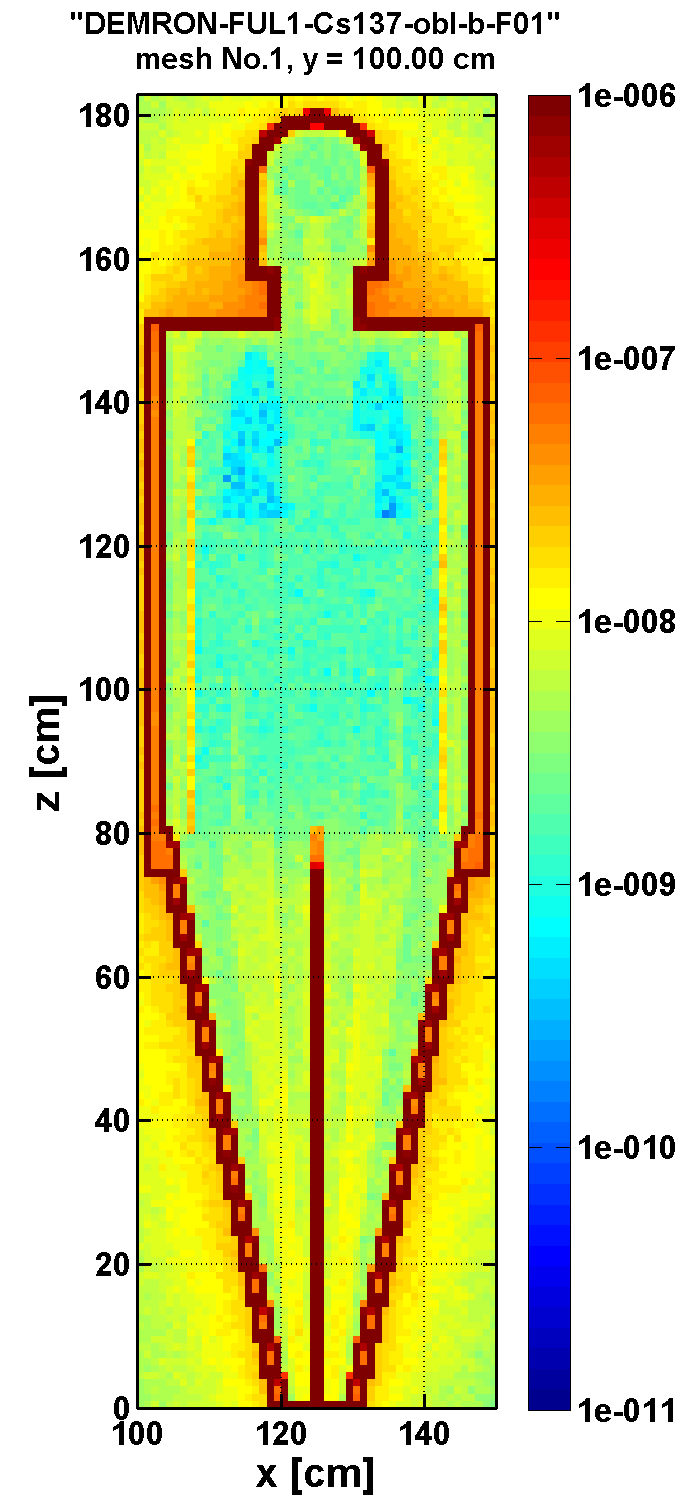

Supplement: Supplementary Materials — The electronic annex presents (1) numerical decrease of the various organs' contribution to the effective dose when protected with individual tested PPE exposed to the other simulated radionuclides dispersed in 10 m3 of the atmosphere in the RAC geometry (Table 2) and (2) visualisations of simulated ORNL phantom energy depositions while only wearing PPE preventing radioactive contamination, and the same PPE together with individual PPE protecting against X- and gamma-ray under it, in a various-dispersed radionuclide's aerosol atmosphere. [file 1641895.f1.zip › 1641895.f1/Electronic annex/Visualization of 2D distributions/Beta contribution/XZ/XZ-b-ppe-Cs137-DEMRON-FUL1.png]

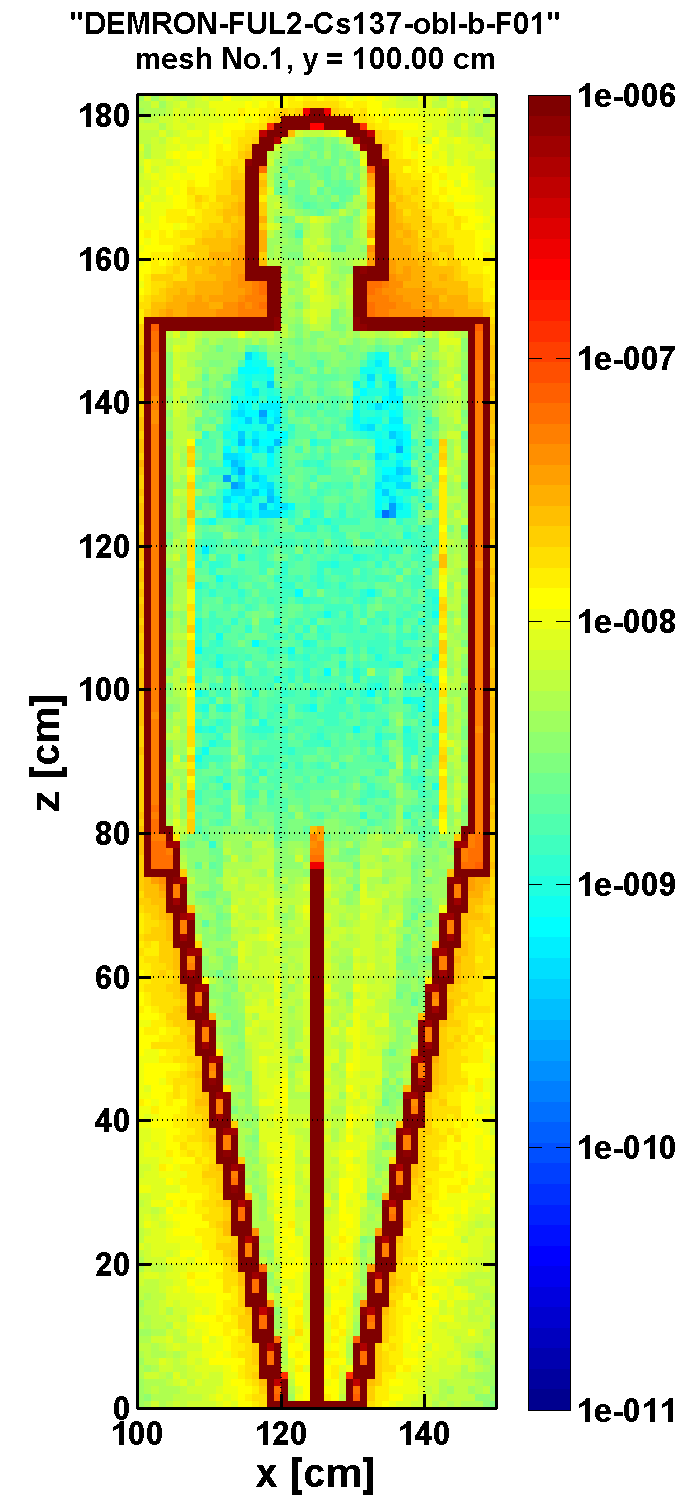

Supplement: Supplementary Materials — The electronic annex presents (1) numerical decrease of the various organs' contribution to the effective dose when protected with individual tested PPE exposed to the other simulated radionuclides dispersed in 10 m3 of the atmosphere in the RAC geometry (Table 2) and (2) visualisations of simulated ORNL phantom energy depositions while only wearing PPE preventing radioactive contamination, and the same PPE together with individual PPE protecting against X- and gamma-ray under it, in a various-dispersed radionuclide's aerosol atmosphere. [file 1641895.f1.zip › 1641895.f1/Electronic annex/Visualization of 2D distributions/Beta contribution/XZ/XZ-b-ppe-Cs137-DEMRON-FUL2.png]

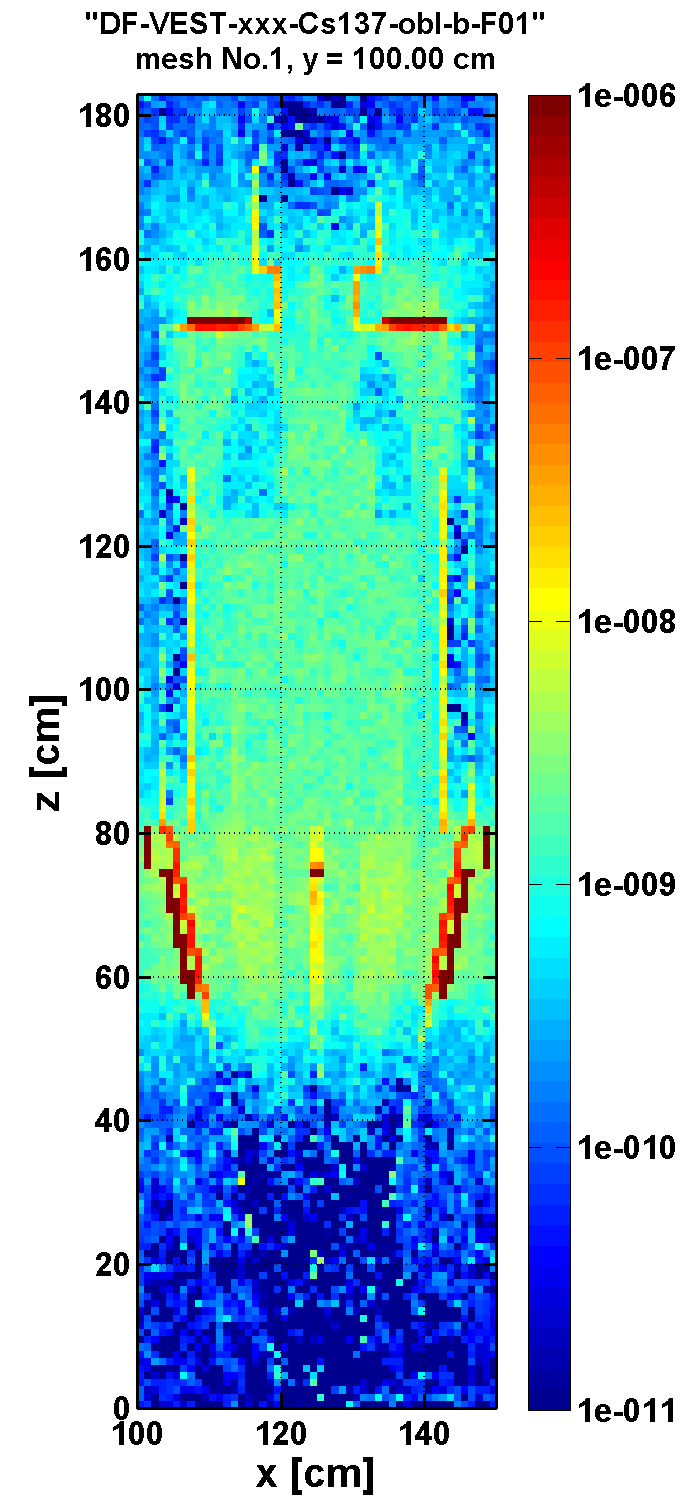

Supplement: Supplementary Materials — The electronic annex presents (1) numerical decrease of the various organs' contribution to the effective dose when protected with individual tested PPE exposed to the other simulated radionuclides dispersed in 10 m3 of the atmosphere in the RAC geometry (Table 2) and (2) visualisations of simulated ORNL phantom energy depositions while only wearing PPE preventing radioactive contamination, and the same PPE together with individual PPE protecting against X- and gamma-ray under it, in a various-dispersed radionuclide's aerosol atmosphere. [file 1641895.f1.zip › 1641895.f1/Electronic annex/Visualization of 2D distributions/Beta contribution/XZ/XZ-b-ppe-Cs137-DF-VEST.png]

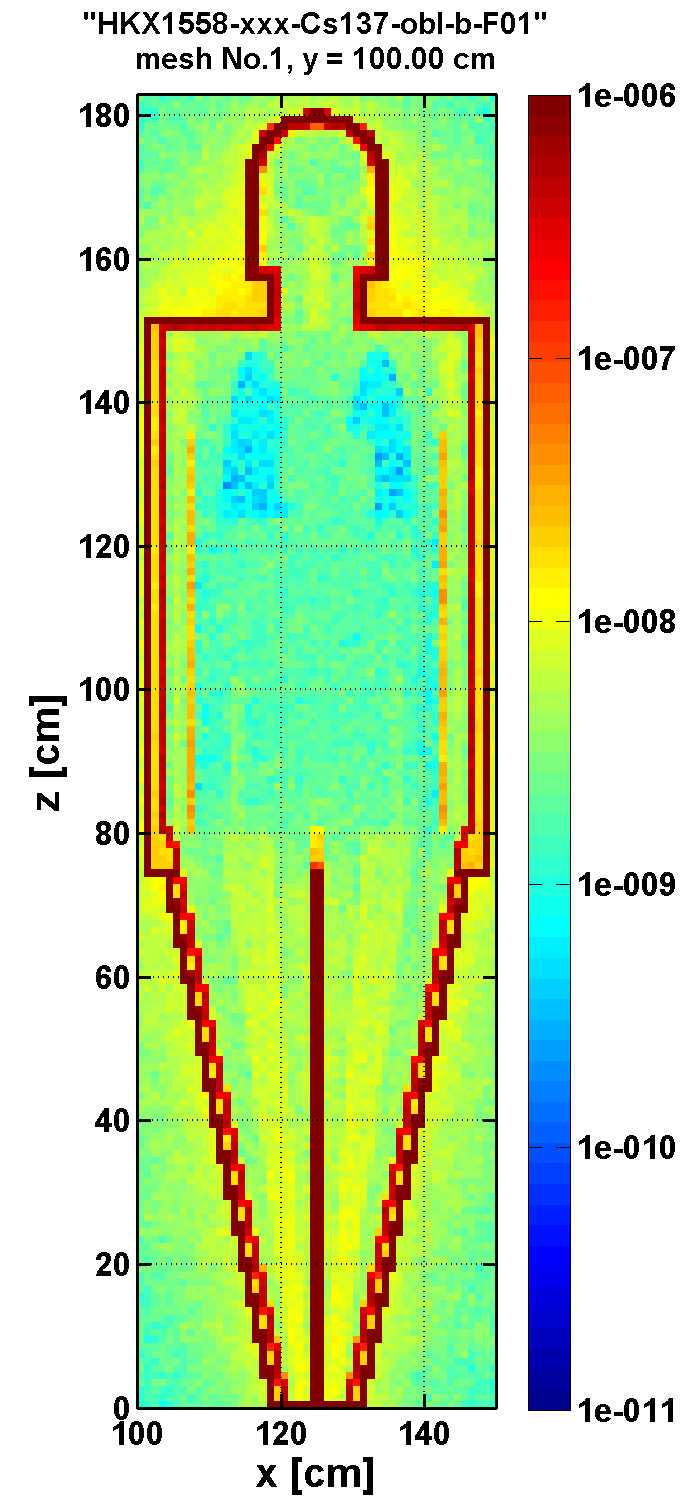

Supplement: Supplementary Materials — The electronic annex presents (1) numerical decrease of the various organs' contribution to the effective dose when protected with individual tested PPE exposed to the other simulated radionuclides dispersed in 10 m3 of the atmosphere in the RAC geometry (Table 2) and (2) visualisations of simulated ORNL phantom energy depositions while only wearing PPE preventing radioactive contamination, and the same PPE together with individual PPE protecting against X- and gamma-ray under it, in a various-dispersed radionuclide's aerosol atmosphere. [file 1641895.f1.zip › 1641895.f1/Electronic annex/Visualization of 2D distributions/Beta contribution/XZ/XZ-b-ppe-Cs137-HKX1558.png]

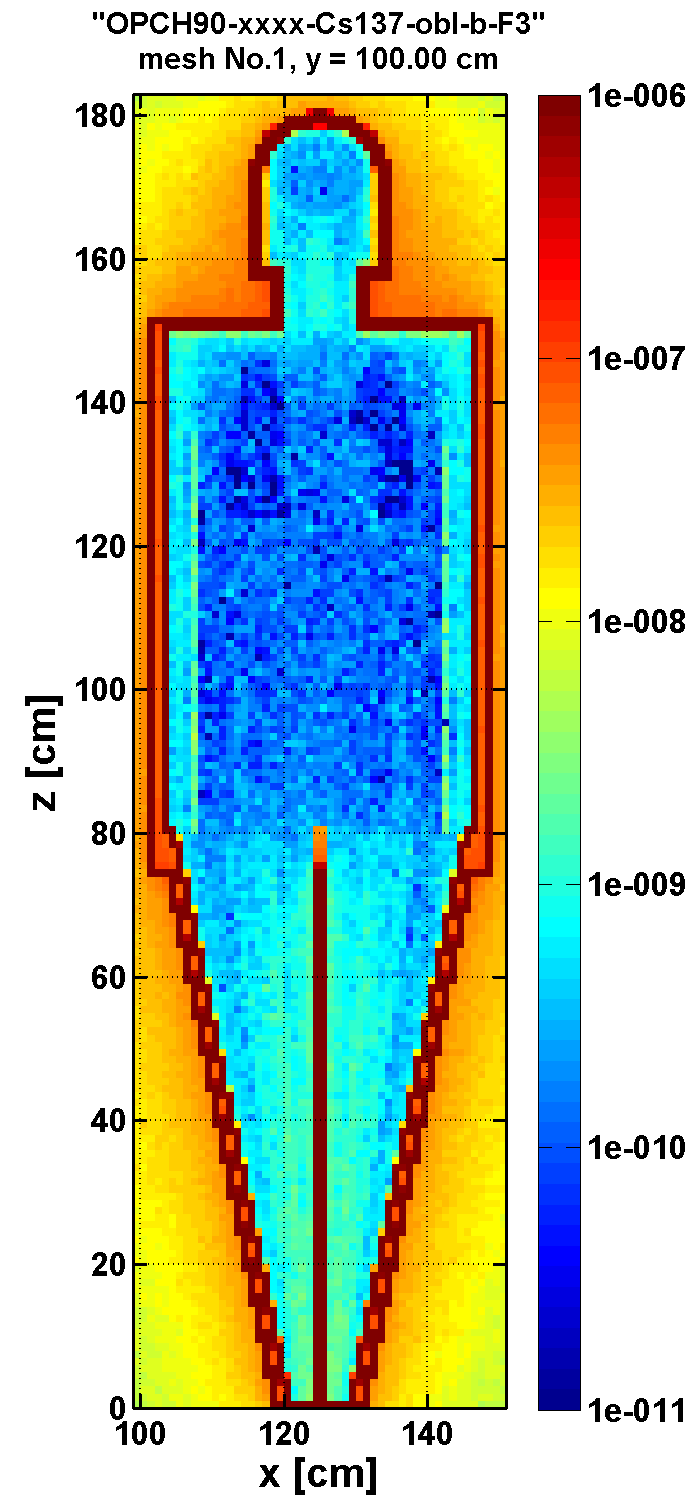

Supplement: Supplementary Materials — The electronic annex presents (1) numerical decrease of the various organs' contribution to the effective dose when protected with individual tested PPE exposed to the other simulated radionuclides dispersed in 10 m3 of the atmosphere in the RAC geometry (Table 2) and (2) visualisations of simulated ORNL phantom energy depositions while only wearing PPE preventing radioactive contamination, and the same PPE together with individual PPE protecting against X- and gamma-ray under it, in a various-dispersed radionuclide's aerosol atmosphere. [file 1641895.f1.zip › 1641895.f1/Electronic annex/Visualization of 2D distributions/Beta contribution/XZ/XZ-b-ppe-Cs137-OPCH90.png]

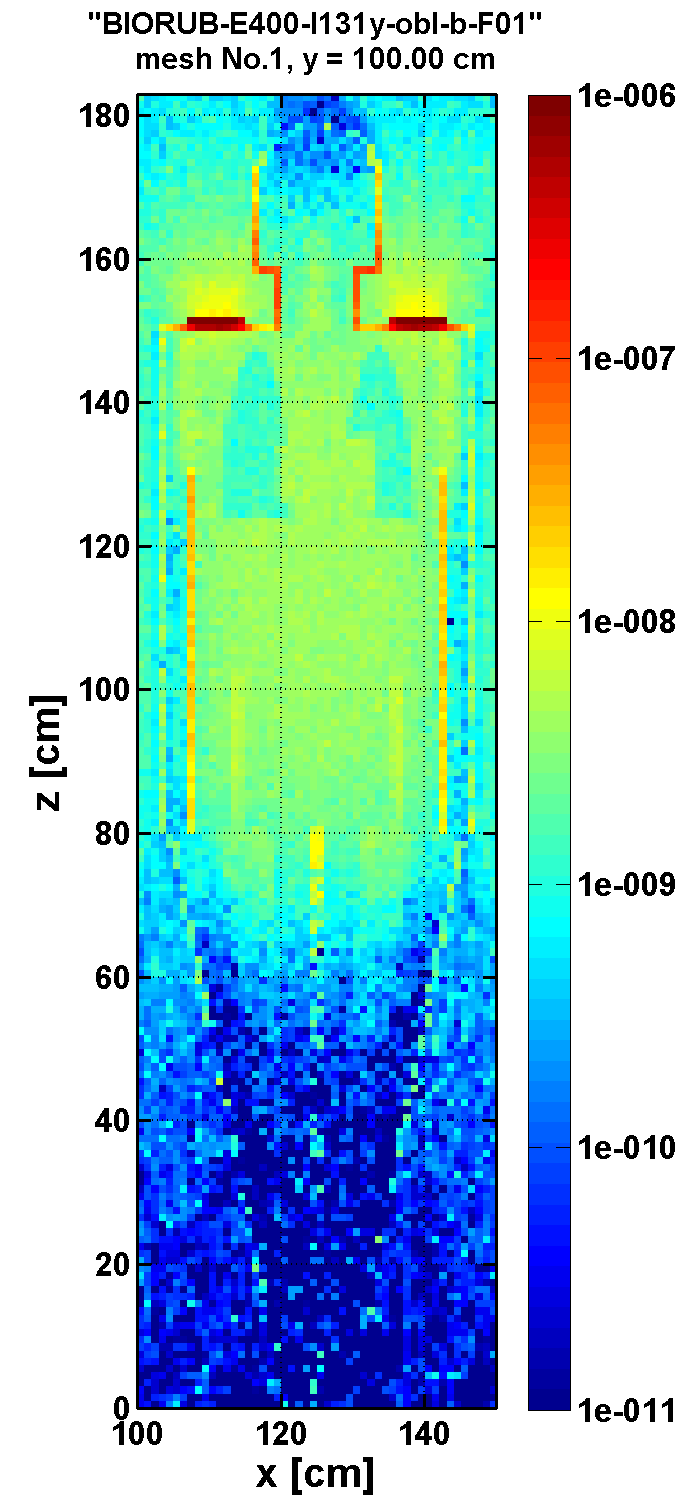

Supplement: Supplementary Materials — The electronic annex presents (1) numerical decrease of the various organs' contribution to the effective dose when protected with individual tested PPE exposed to the other simulated radionuclides dispersed in 10 m3 of the atmosphere in the RAC geometry (Table 2) and (2) visualisations of simulated ORNL phantom energy depositions while only wearing PPE preventing radioactive contamination, and the same PPE together with individual PPE protecting against X- and gamma-ray under it, in a various-dispersed radionuclide's aerosol atmosphere. [file 1641895.f1.zip › 1641895.f1/Electronic annex/Visualization of 2D distributions/Beta contribution/XZ/XZ-b-ppe-I131-BIORUB-E400.png]

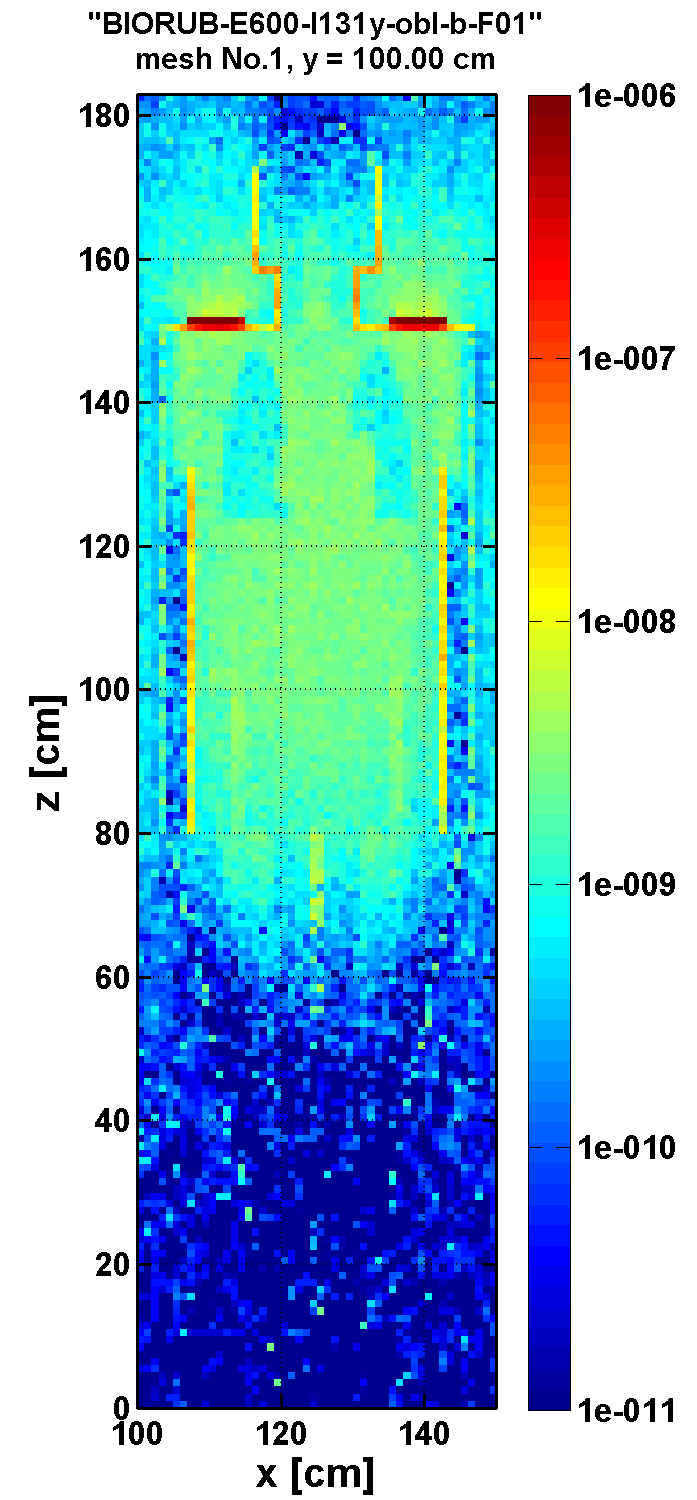

Supplement: Supplementary Materials — The electronic annex presents (1) numerical decrease of the various organs' contribution to the effective dose when protected with individual tested PPE exposed to the other simulated radionuclides dispersed in 10 m3 of the atmosphere in the RAC geometry (Table 2) and (2) visualisations of simulated ORNL phantom energy depositions while only wearing PPE preventing radioactive contamination, and the same PPE together with individual PPE protecting against X- and gamma-ray under it, in a various-dispersed radionuclide's aerosol atmosphere. [file 1641895.f1.zip › 1641895.f1/Electronic annex/Visualization of 2D distributions/Beta contribution/XZ/XZ-b-ppe-I131-BIORUB-E600.png]

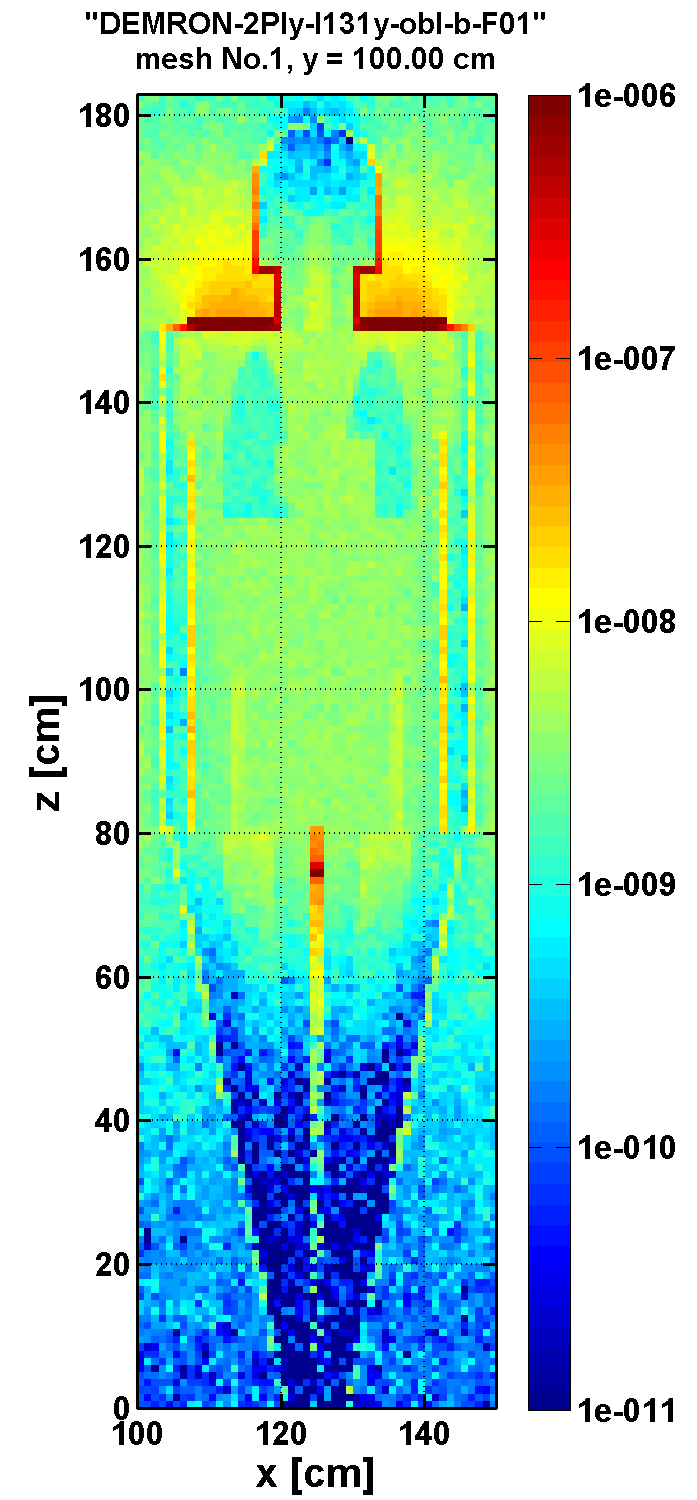

Supplement: Supplementary Materials — The electronic annex presents (1) numerical decrease of the various organs' contribution to the effective dose when protected with individual tested PPE exposed to the other simulated radionuclides dispersed in 10 m3 of the atmosphere in the RAC geometry (Table 2) and (2) visualisations of simulated ORNL phantom energy depositions while only wearing PPE preventing radioactive contamination, and the same PPE together with individual PPE protecting against X- and gamma-ray under it, in a various-dispersed radionuclide's aerosol atmosphere. [file 1641895.f1.zip › 1641895.f1/Electronic annex/Visualization of 2D distributions/Beta contribution/XZ/XZ-b-ppe-I131-DEMRON-2Ply.png]

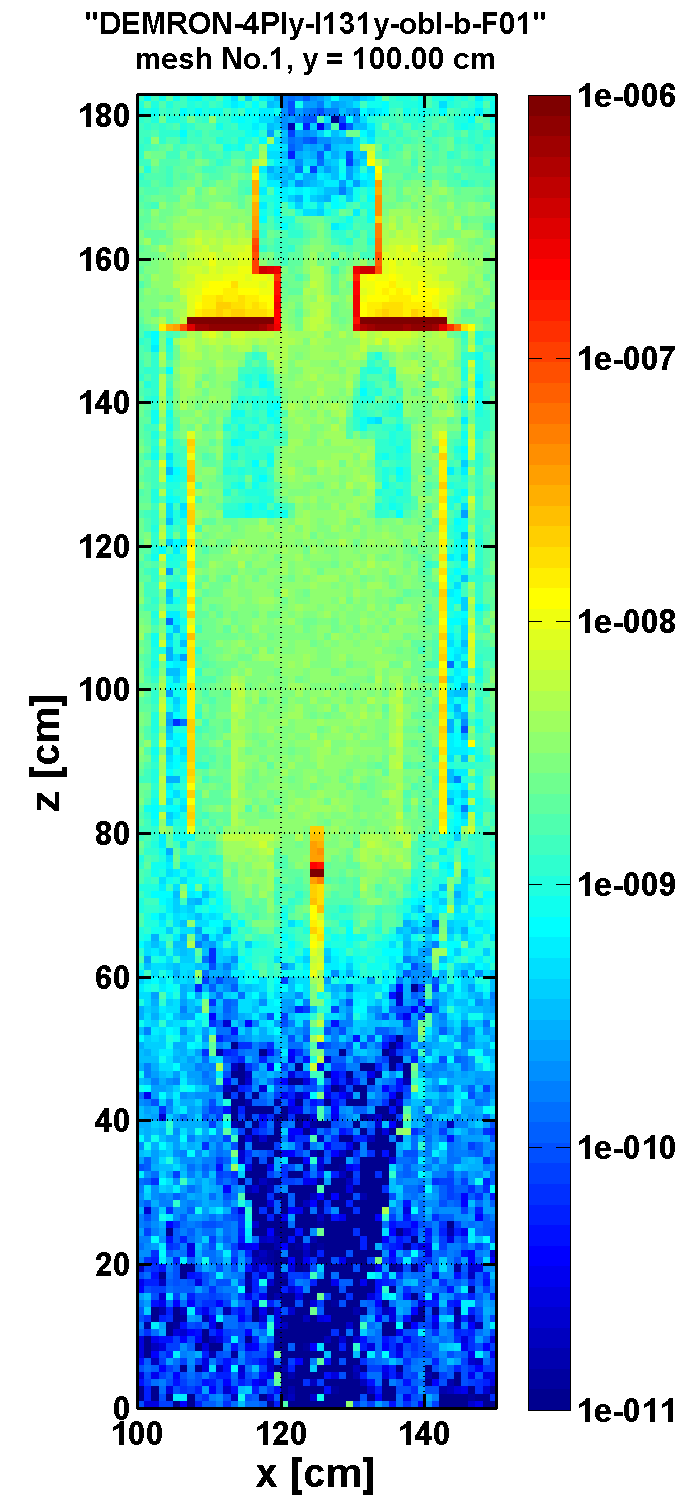

Supplement: Supplementary Materials — The electronic annex presents (1) numerical decrease of the various organs' contribution to the effective dose when protected with individual tested PPE exposed to the other simulated radionuclides dispersed in 10 m3 of the atmosphere in the RAC geometry (Table 2) and (2) visualisations of simulated ORNL phantom energy depositions while only wearing PPE preventing radioactive contamination, and the same PPE together with individual PPE protecting against X- and gamma-ray under it, in a various-dispersed radionuclide's aerosol atmosphere. [file 1641895.f1.zip › 1641895.f1/Electronic annex/Visualization of 2D distributions/Beta contribution/XZ/XZ-b-ppe-I131-DEMRON-4Ply.png]

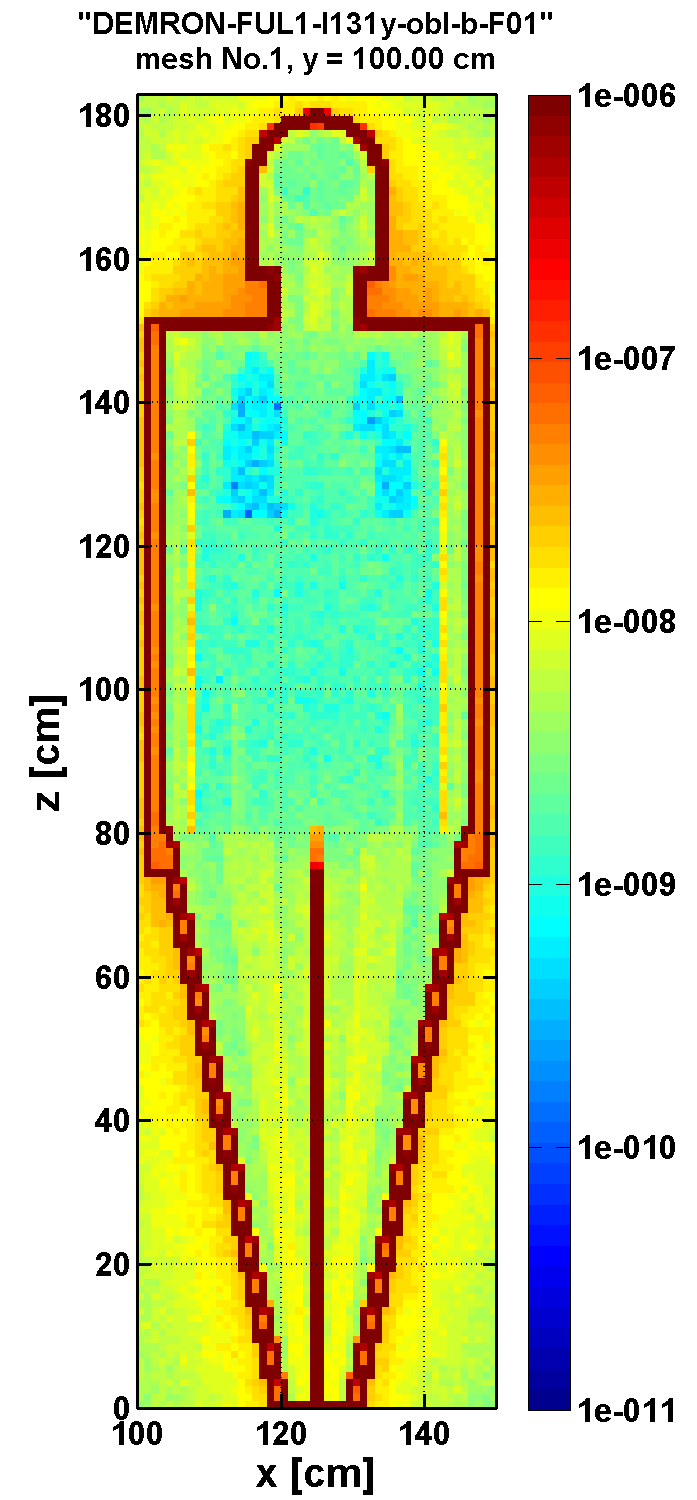

Supplement: Supplementary Materials — The electronic annex presents (1) numerical decrease of the various organs' contribution to the effective dose when protected with individual tested PPE exposed to the other simulated radionuclides dispersed in 10 m3 of the atmosphere in the RAC geometry (Table 2) and (2) visualisations of simulated ORNL phantom energy depositions while only wearing PPE preventing radioactive contamination, and the same PPE together with individual PPE protecting against X- and gamma-ray under it, in a various-dispersed radionuclide's aerosol atmosphere. [file 1641895.f1.zip › 1641895.f1/Electronic annex/Visualization of 2D distributions/Beta contribution/XZ/XZ-b-ppe-I131-DEMRON-FUL1.png]

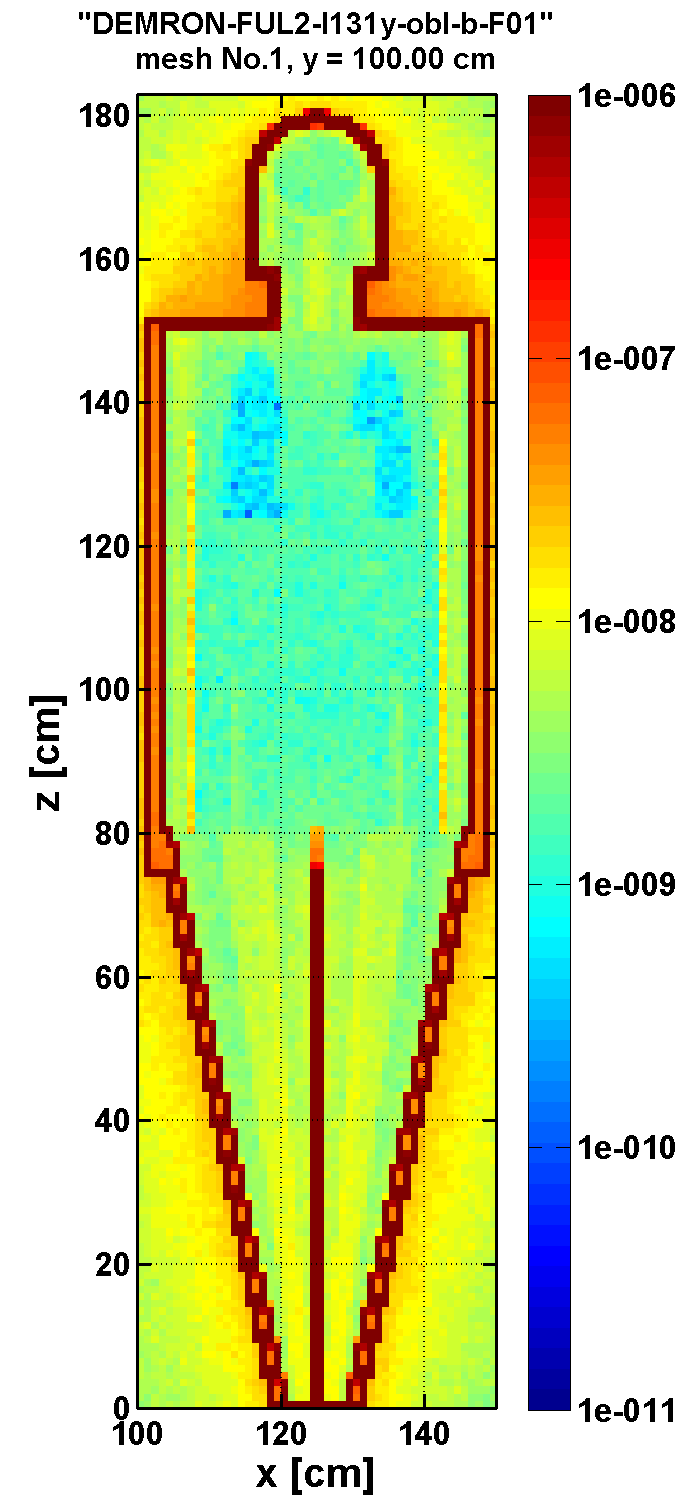

Supplement: Supplementary Materials — The electronic annex presents (1) numerical decrease of the various organs' contribution to the effective dose when protected with individual tested PPE exposed to the other simulated radionuclides dispersed in 10 m3 of the atmosphere in the RAC geometry (Table 2) and (2) visualisations of simulated ORNL phantom energy depositions while only wearing PPE preventing radioactive contamination, and the same PPE together with individual PPE protecting against X- and gamma-ray under it, in a various-dispersed radionuclide's aerosol atmosphere. [file 1641895.f1.zip › 1641895.f1/Electronic annex/Visualization of 2D distributions/Beta contribution/XZ/XZ-b-ppe-I131-DEMRON-FUL2.png]

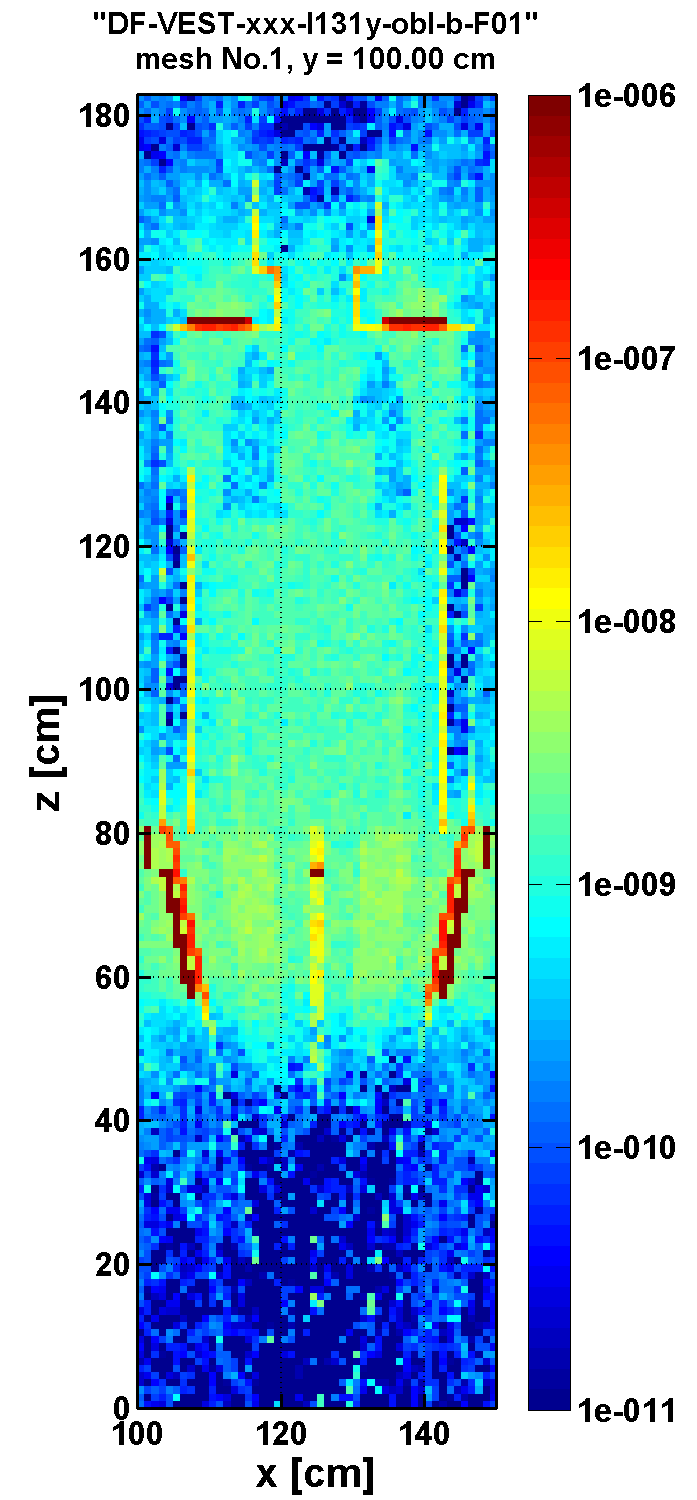

Supplement: Supplementary Materials — The electronic annex presents (1) numerical decrease of the various organs' contribution to the effective dose when protected with individual tested PPE exposed to the other simulated radionuclides dispersed in 10 m3 of the atmosphere in the RAC geometry (Table 2) and (2) visualisations of simulated ORNL phantom energy depositions while only wearing PPE preventing radioactive contamination, and the same PPE together with individual PPE protecting against X- and gamma-ray under it, in a various-dispersed radionuclide's aerosol atmosphere. [file 1641895.f1.zip › 1641895.f1/Electronic annex/Visualization of 2D distributions/Beta contribution/XZ/XZ-b-ppe-I131-DF-VEST.png]

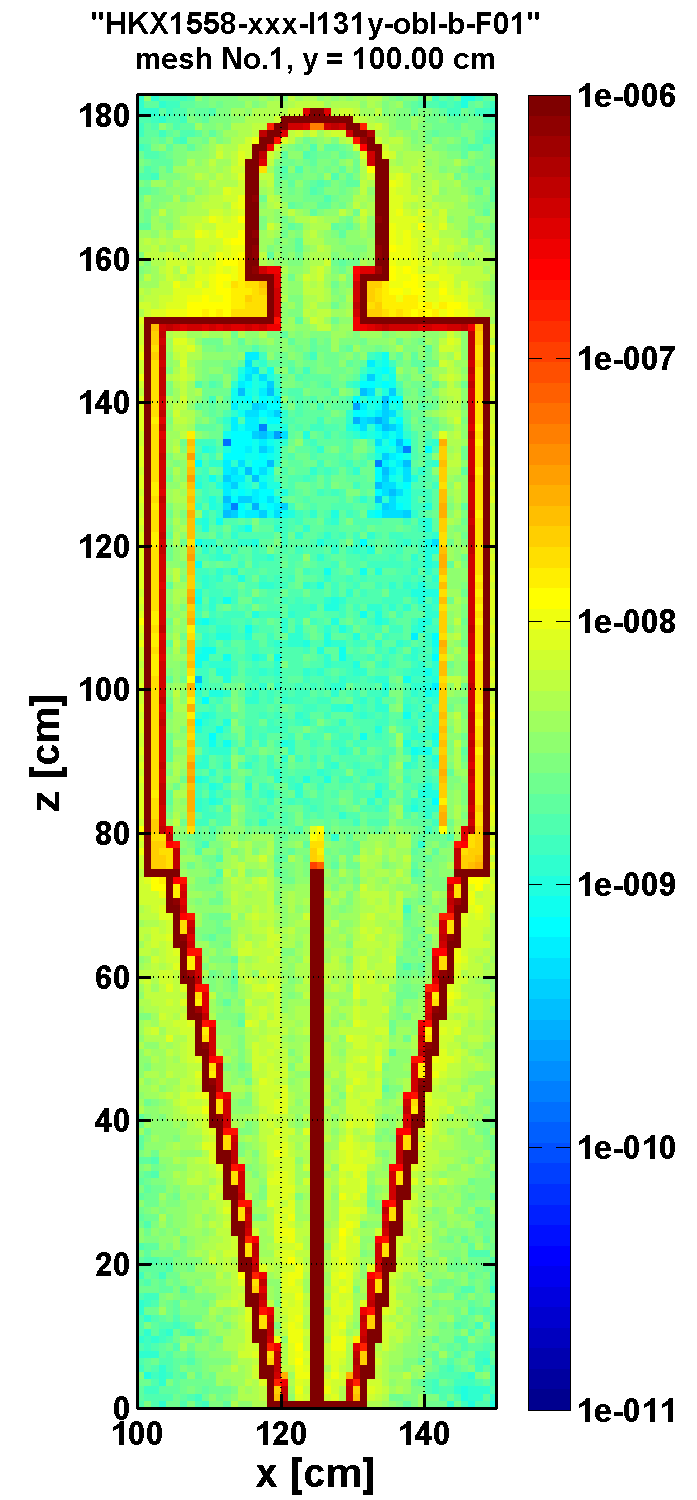

Supplement: Supplementary Materials — The electronic annex presents (1) numerical decrease of the various organs' contribution to the effective dose when protected with individual tested PPE exposed to the other simulated radionuclides dispersed in 10 m3 of the atmosphere in the RAC geometry (Table 2) and (2) visualisations of simulated ORNL phantom energy depositions while only wearing PPE preventing radioactive contamination, and the same PPE together with individual PPE protecting against X- and gamma-ray under it, in a various-dispersed radionuclide's aerosol atmosphere. [file 1641895.f1.zip › 1641895.f1/Electronic annex/Visualization of 2D distributions/Beta contribution/XZ/XZ-b-ppe-I131-HKX1558.png]

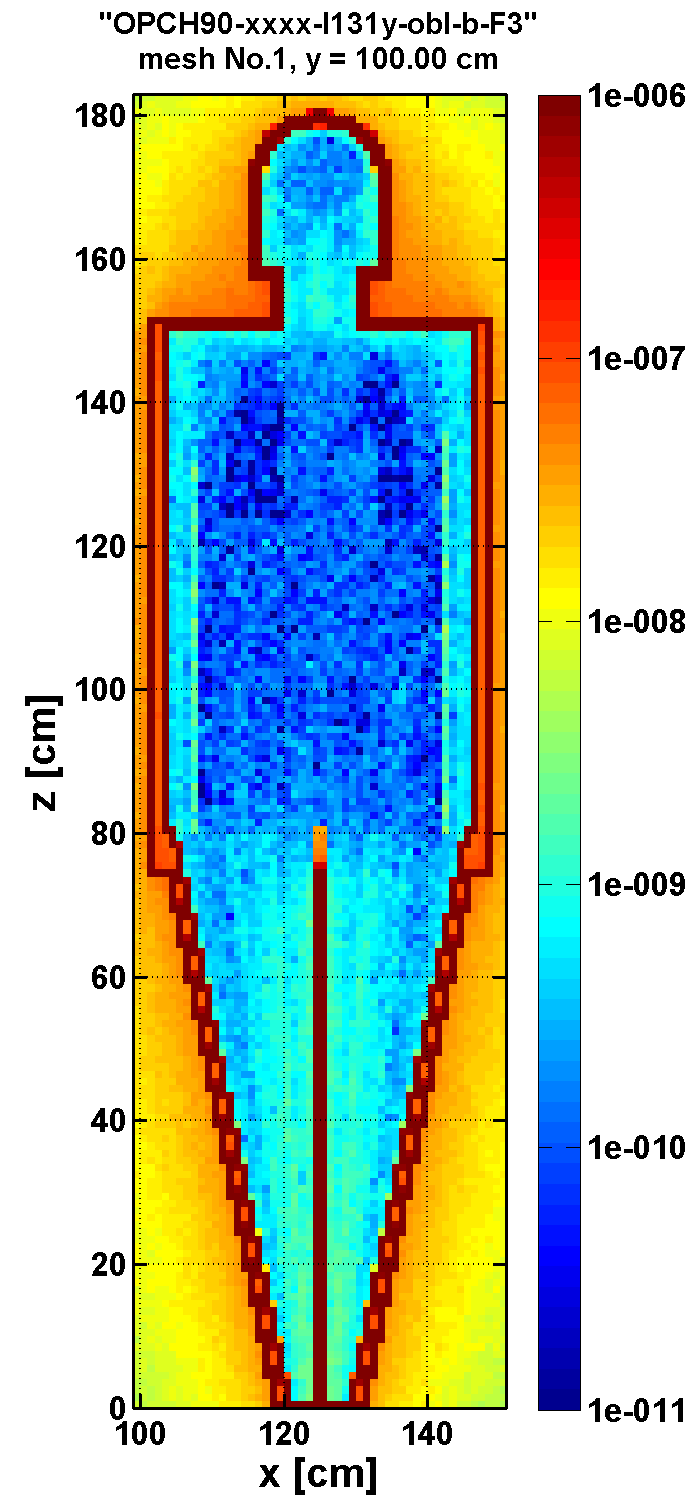

Supplement: Supplementary Materials — The electronic annex presents (1) numerical decrease of the various organs' contribution to the effective dose when protected with individual tested PPE exposed to the other simulated radionuclides dispersed in 10 m3 of the atmosphere in the RAC geometry (Table 2) and (2) visualisations of simulated ORNL phantom energy depositions while only wearing PPE preventing radioactive contamination, and the same PPE together with individual PPE protecting against X- and gamma-ray under it, in a various-dispersed radionuclide's aerosol atmosphere. [file 1641895.f1.zip › 1641895.f1/Electronic annex/Visualization of 2D distributions/Beta contribution/XZ/XZ-b-ppe-I131-OPCH90.png]

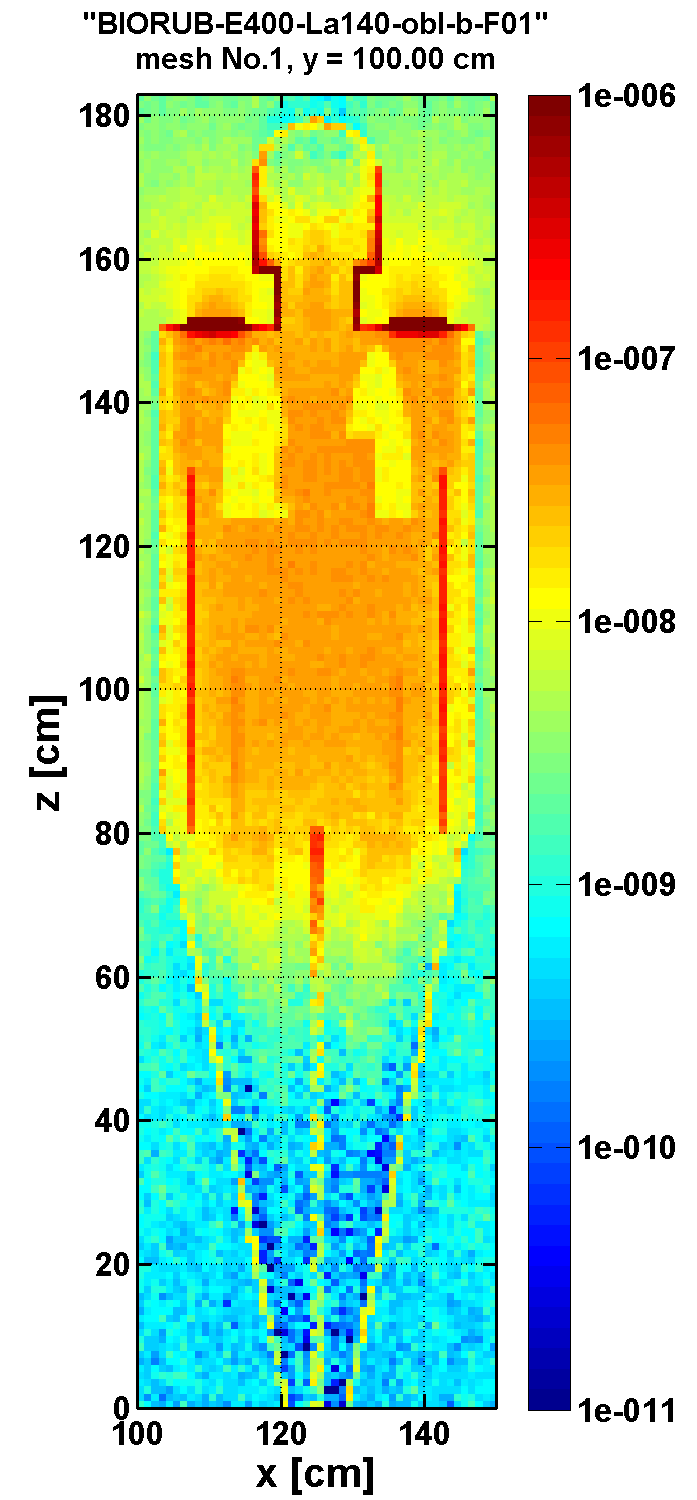

Supplement: Supplementary Materials — The electronic annex presents (1) numerical decrease of the various organs' contribution to the effective dose when protected with individual tested PPE exposed to the other simulated radionuclides dispersed in 10 m3 of the atmosphere in the RAC geometry (Table 2) and (2) visualisations of simulated ORNL phantom energy depositions while only wearing PPE preventing radioactive contamination, and the same PPE together with individual PPE protecting against X- and gamma-ray under it, in a various-dispersed radionuclide's aerosol atmosphere. [file 1641895.f1.zip › 1641895.f1/Electronic annex/Visualization of 2D distributions/Beta contribution/XZ/XZ-b-ppe-La140-BIORUB-E400.png]

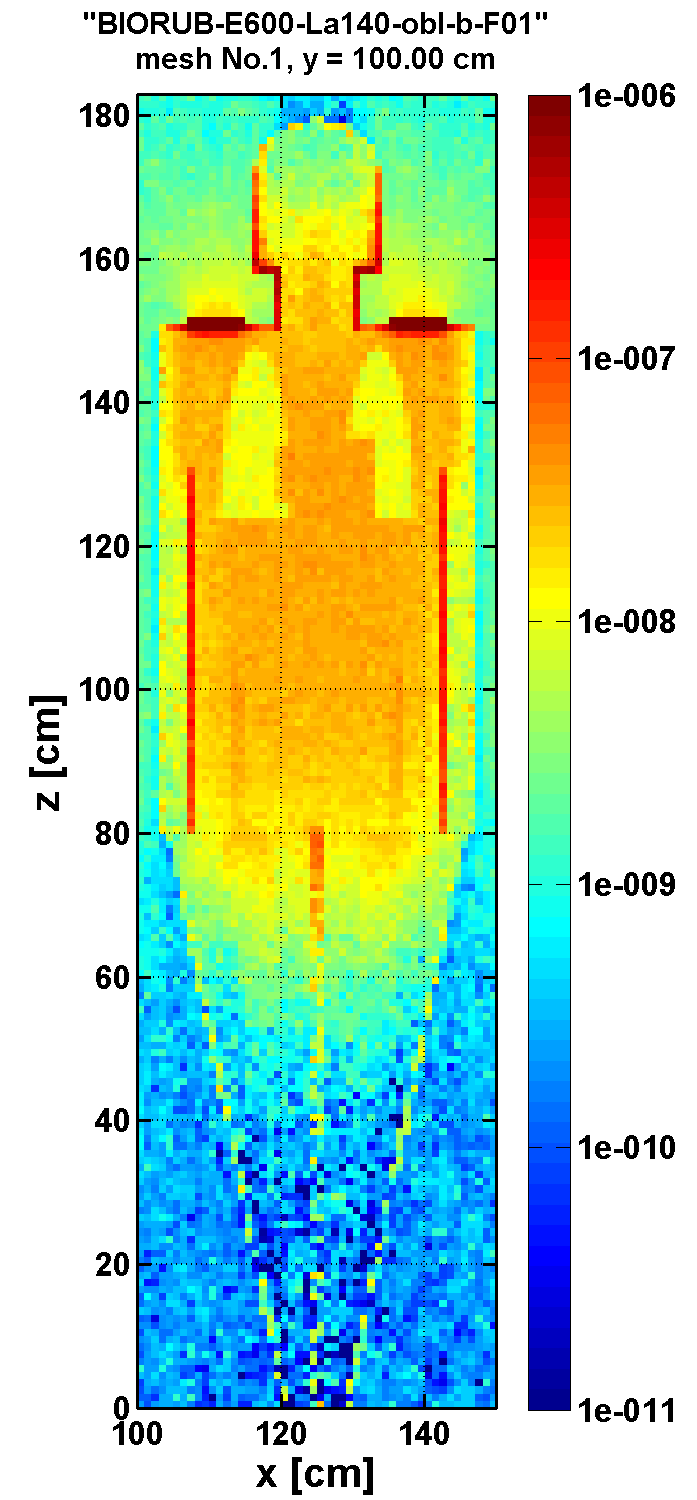

Supplement: Supplementary Materials — The electronic annex presents (1) numerical decrease of the various organs' contribution to the effective dose when protected with individual tested PPE exposed to the other simulated radionuclides dispersed in 10 m3 of the atmosphere in the RAC geometry (Table 2) and (2) visualisations of simulated ORNL phantom energy depositions while only wearing PPE preventing radioactive contamination, and the same PPE together with individual PPE protecting against X- and gamma-ray under it, in a various-dispersed radionuclide's aerosol atmosphere. [file 1641895.f1.zip › 1641895.f1/Electronic annex/Visualization of 2D distributions/Beta contribution/XZ/XZ-b-ppe-La140-BIORUB-E600.png]

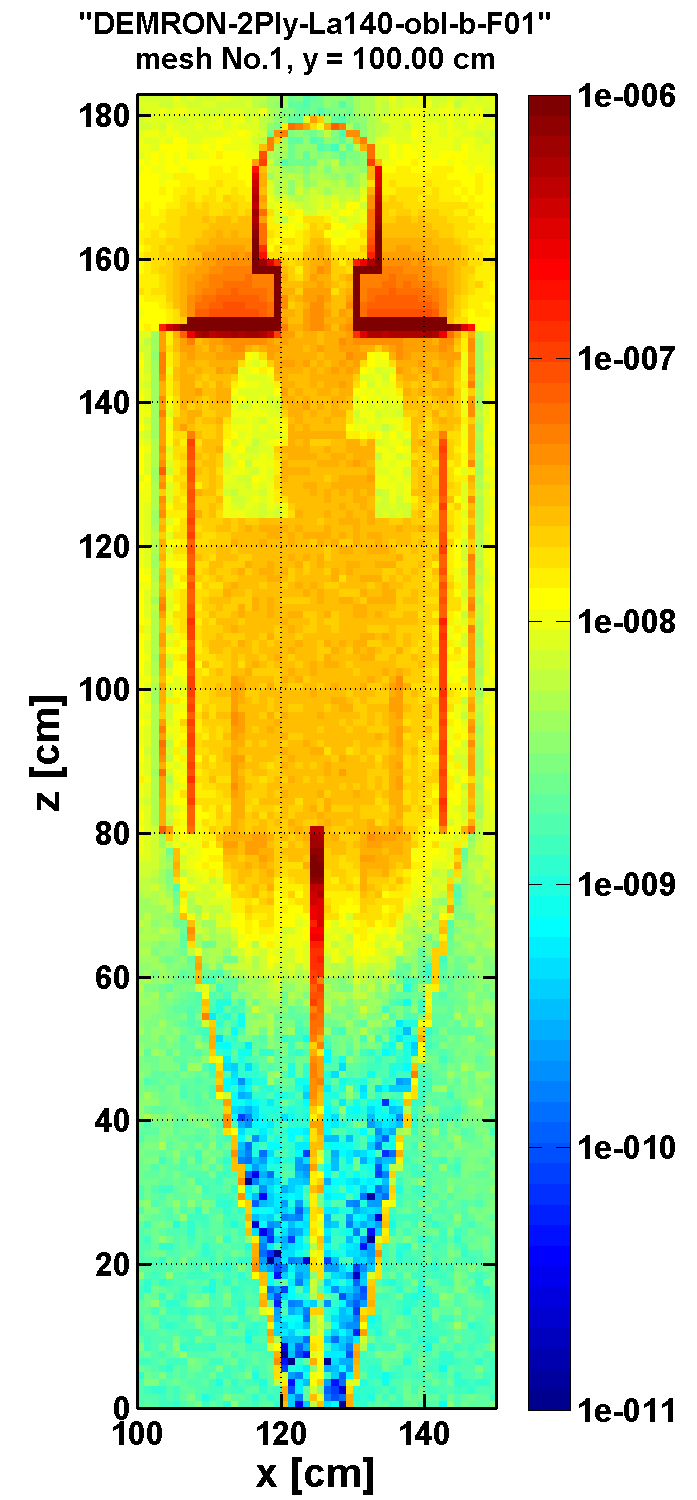

Supplement: Supplementary Materials — The electronic annex presents (1) numerical decrease of the various organs' contribution to the effective dose when protected with individual tested PPE exposed to the other simulated radionuclides dispersed in 10 m3 of the atmosphere in the RAC geometry (Table 2) and (2) visualisations of simulated ORNL phantom energy depositions while only wearing PPE preventing radioactive contamination, and the same PPE together with individual PPE protecting against X- and gamma-ray under it, in a various-dispersed radionuclide's aerosol atmosphere. [file 1641895.f1.zip › 1641895.f1/Electronic annex/Visualization of 2D distributions/Beta contribution/XZ/XZ-b-ppe-La140-DEMRON-2Ply.png]

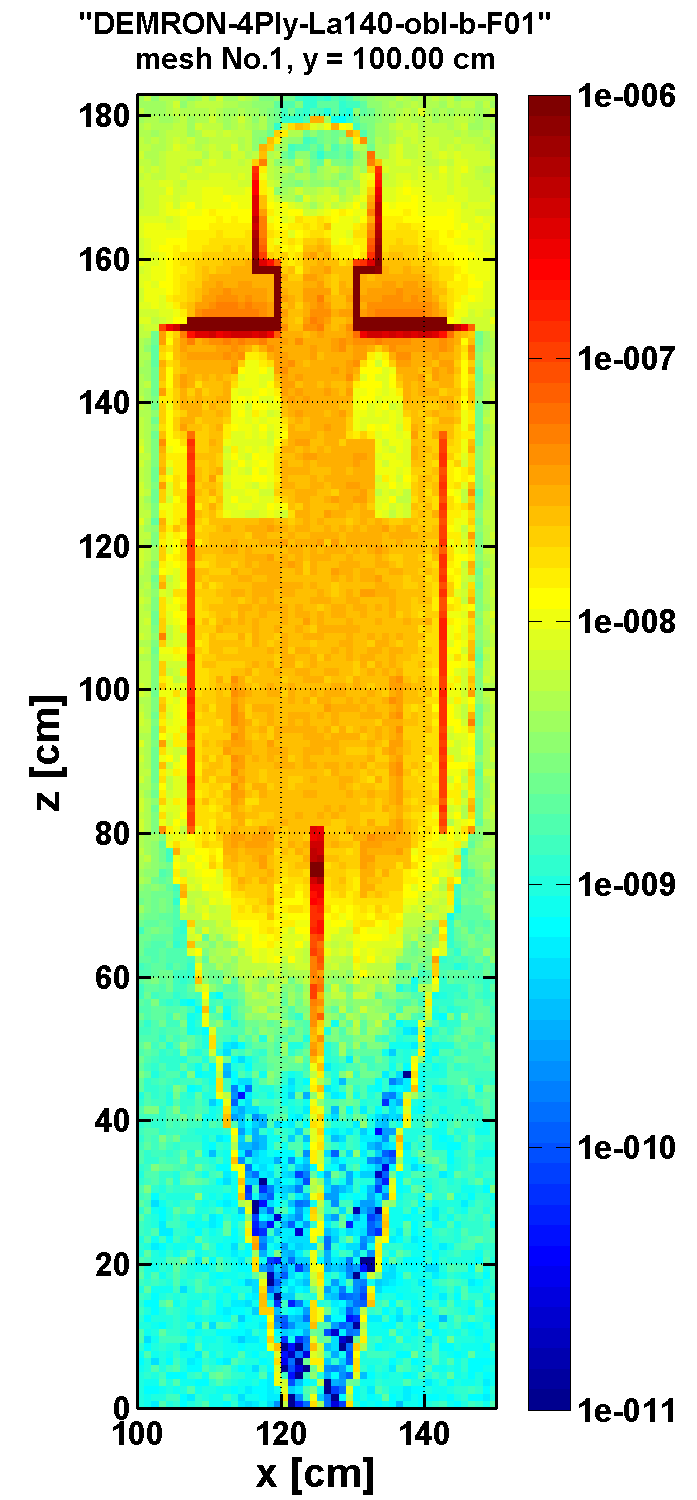

Supplement: Supplementary Materials — The electronic annex presents (1) numerical decrease of the various organs' contribution to the effective dose when protected with individual tested PPE exposed to the other simulated radionuclides dispersed in 10 m3 of the atmosphere in the RAC geometry (Table 2) and (2) visualisations of simulated ORNL phantom energy depositions while only wearing PPE preventing radioactive contamination, and the same PPE together with individual PPE protecting against X- and gamma-ray under it, in a various-dispersed radionuclide's aerosol atmosphere. [file 1641895.f1.zip › 1641895.f1/Electronic annex/Visualization of 2D distributions/Beta contribution/XZ/XZ-b-ppe-La140-DEMRON-4Ply.png]

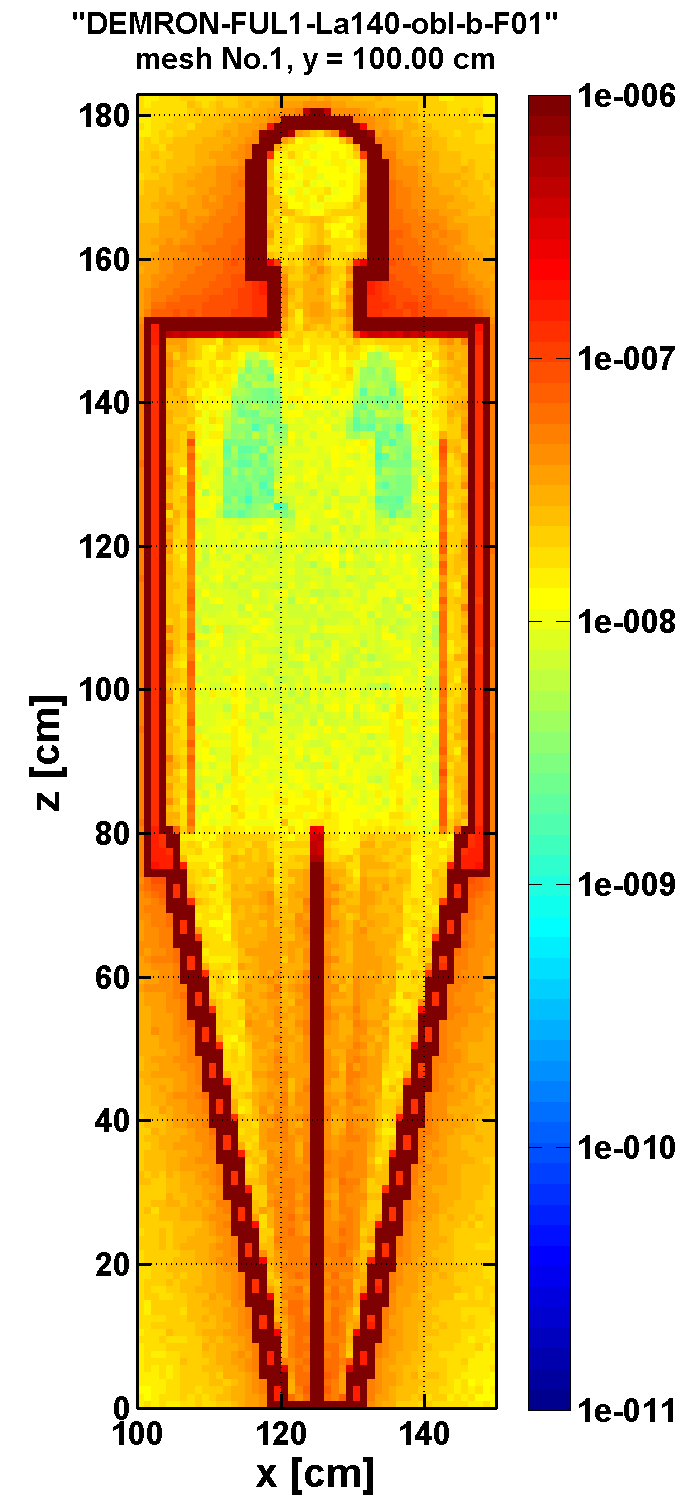

Supplement: Supplementary Materials — The electronic annex presents (1) numerical decrease of the various organs' contribution to the effective dose when protected with individual tested PPE exposed to the other simulated radionuclides dispersed in 10 m3 of the atmosphere in the RAC geometry (Table 2) and (2) visualisations of simulated ORNL phantom energy depositions while only wearing PPE preventing radioactive contamination, and the same PPE together with individual PPE protecting against X- and gamma-ray under it, in a various-dispersed radionuclide's aerosol atmosphere. [file 1641895.f1.zip › 1641895.f1/Electronic annex/Visualization of 2D distributions/Beta contribution/XZ/XZ-b-ppe-La140-DEMRON-FUL1.png]

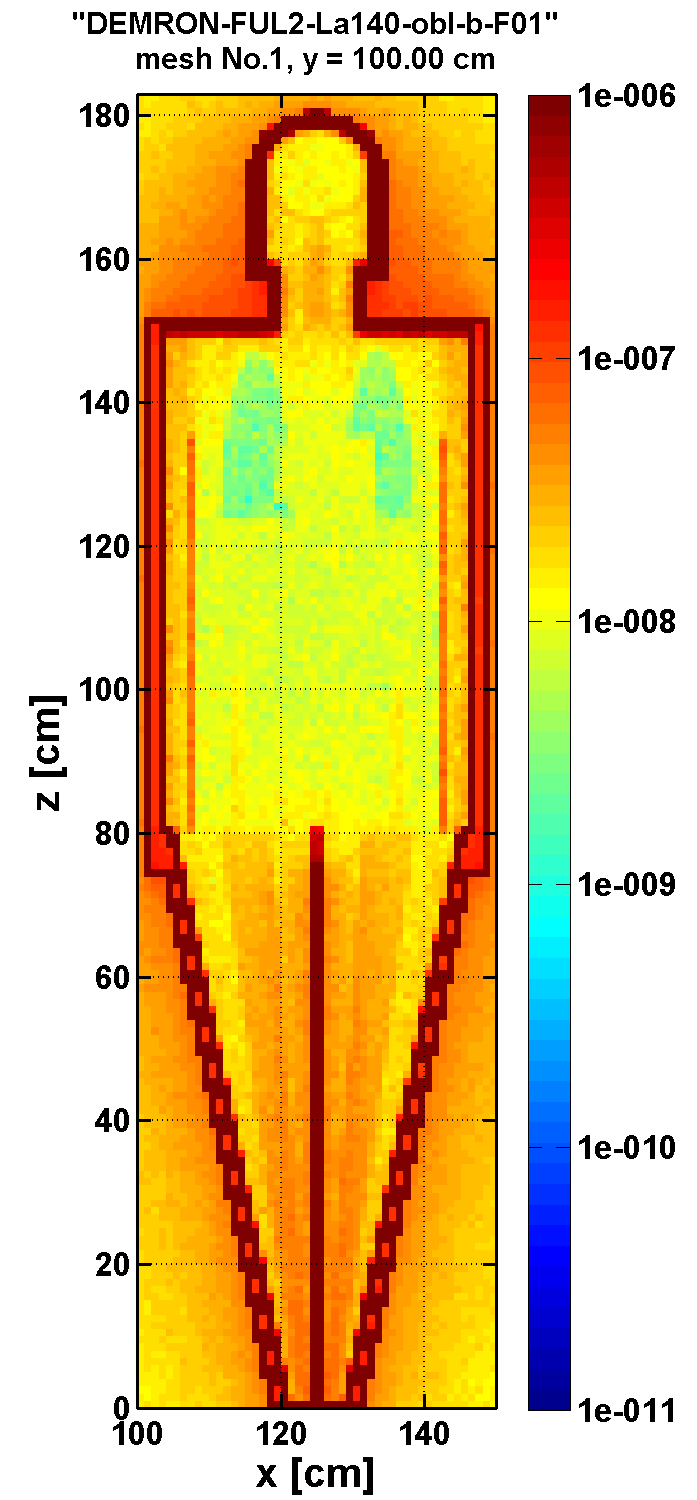

Supplement: Supplementary Materials — The electronic annex presents (1) numerical decrease of the various organs' contribution to the effective dose when protected with individual tested PPE exposed to the other simulated radionuclides dispersed in 10 m3 of the atmosphere in the RAC geometry (Table 2) and (2) visualisations of simulated ORNL phantom energy depositions while only wearing PPE preventing radioactive contamination, and the same PPE together with individual PPE protecting against X- and gamma-ray under it, in a various-dispersed radionuclide's aerosol atmosphere. [file 1641895.f1.zip › 1641895.f1/Electronic annex/Visualization of 2D distributions/Beta contribution/XZ/XZ-b-ppe-La140-DEMRON-FUL2.png]

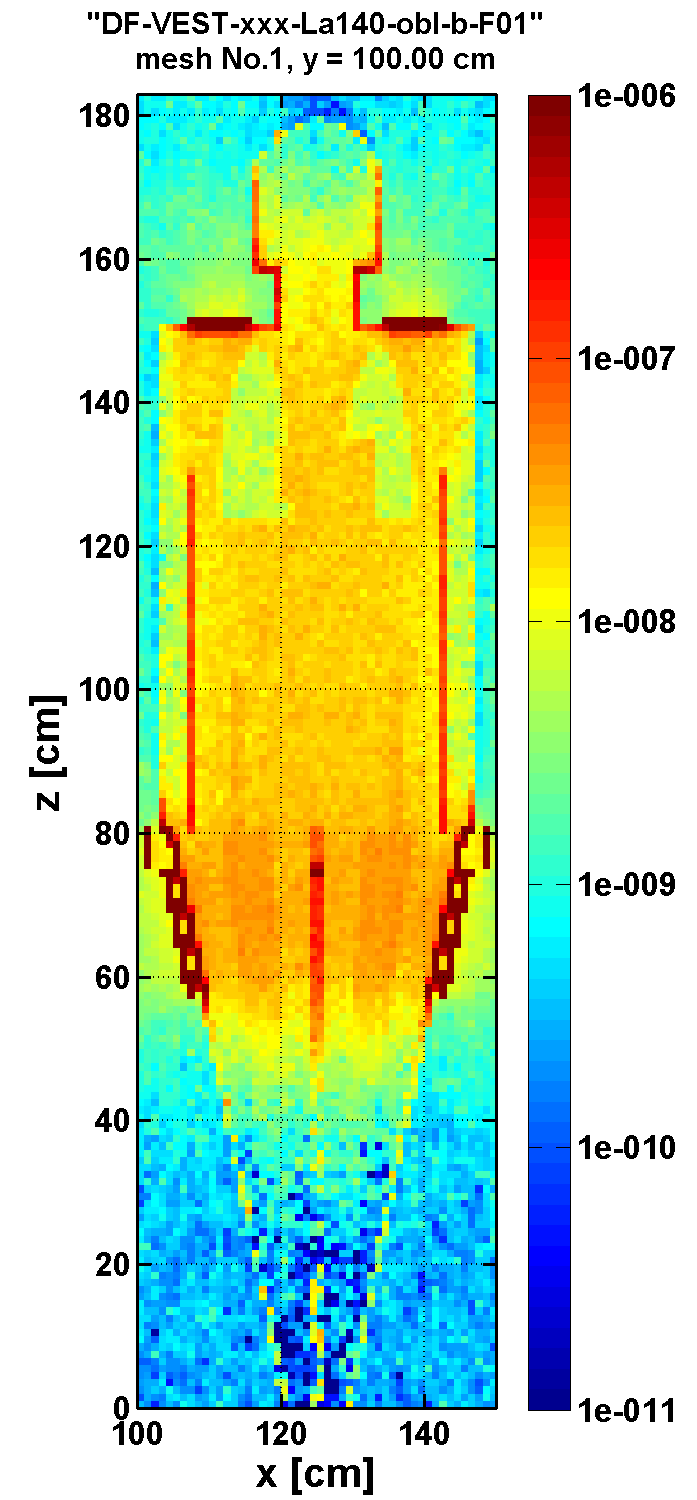

Supplement: Supplementary Materials — The electronic annex presents (1) numerical decrease of the various organs' contribution to the effective dose when protected with individual tested PPE exposed to the other simulated radionuclides dispersed in 10 m3 of the atmosphere in the RAC geometry (Table 2) and (2) visualisations of simulated ORNL phantom energy depositions while only wearing PPE preventing radioactive contamination, and the same PPE together with individual PPE protecting against X- and gamma-ray under it, in a various-dispersed radionuclide's aerosol atmosphere. [file 1641895.f1.zip › 1641895.f1/Electronic annex/Visualization of 2D distributions/Beta contribution/XZ/XZ-b-ppe-La140-DF-VEST.png]

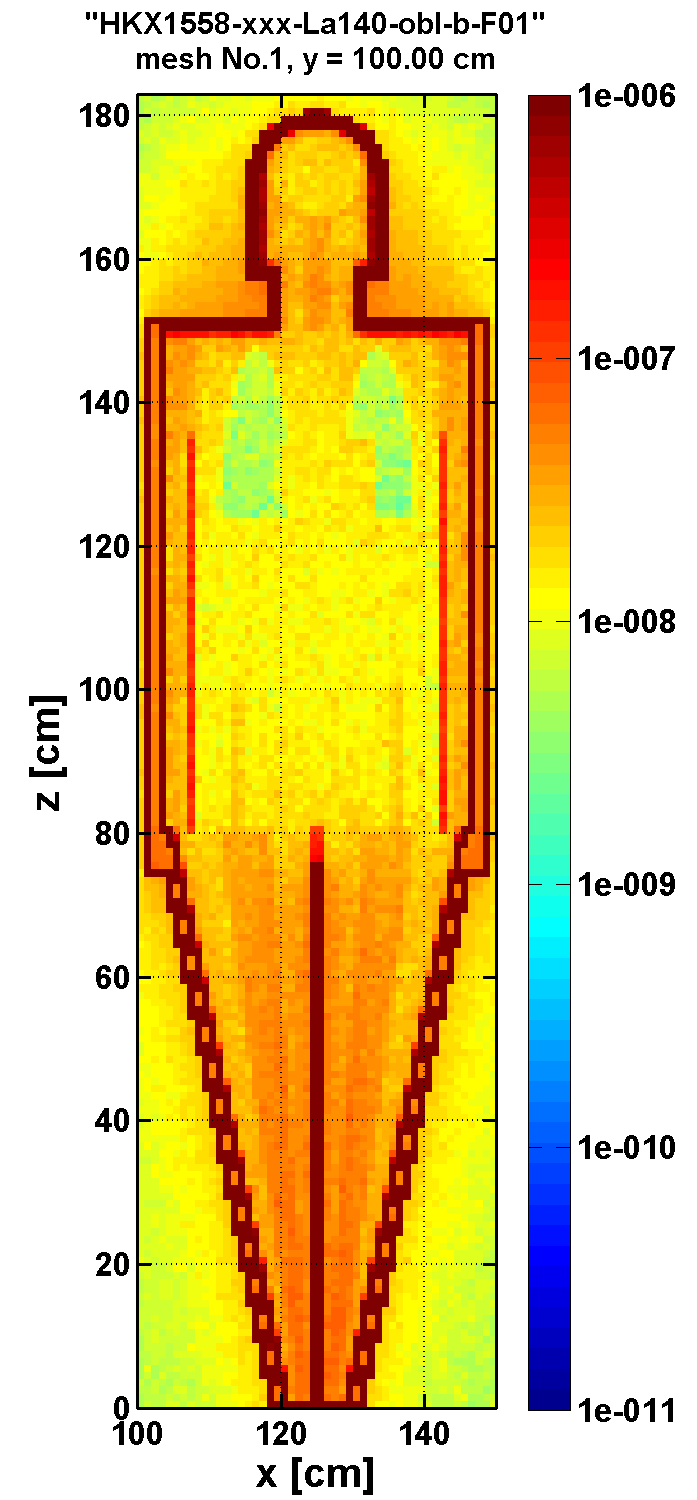

Supplement: Supplementary Materials — The electronic annex presents (1) numerical decrease of the various organs' contribution to the effective dose when protected with individual tested PPE exposed to the other simulated radionuclides dispersed in 10 m3 of the atmosphere in the RAC geometry (Table 2) and (2) visualisations of simulated ORNL phantom energy depositions while only wearing PPE preventing radioactive contamination, and the same PPE together with individual PPE protecting against X- and gamma-ray under it, in a various-dispersed radionuclide's aerosol atmosphere. [file 1641895.f1.zip › 1641895.f1/Electronic annex/Visualization of 2D distributions/Beta contribution/XZ/XZ-b-ppe-La140-HKX1558.png]

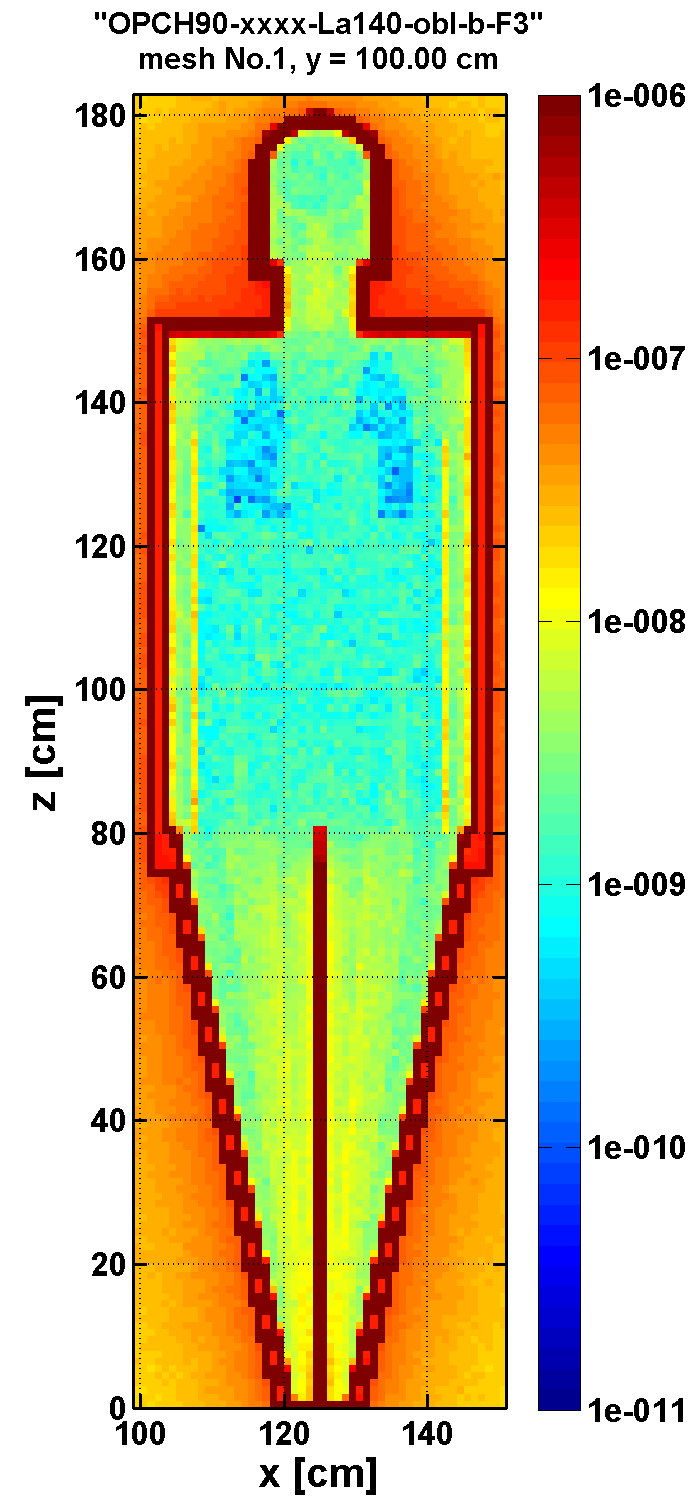

Supplement: Supplementary Materials — The electronic annex presents (1) numerical decrease of the various organs' contribution to the effective dose when protected with individual tested PPE exposed to the other simulated radionuclides dispersed in 10 m3 of the atmosphere in the RAC geometry (Table 2) and (2) visualisations of simulated ORNL phantom energy depositions while only wearing PPE preventing radioactive contamination, and the same PPE together with individual PPE protecting against X- and gamma-ray under it, in a various-dispersed radionuclide's aerosol atmosphere. [file 1641895.f1.zip › 1641895.f1/Electronic annex/Visualization of 2D distributions/Beta contribution/XZ/XZ-b-ppe-La140-OPCH90.png]

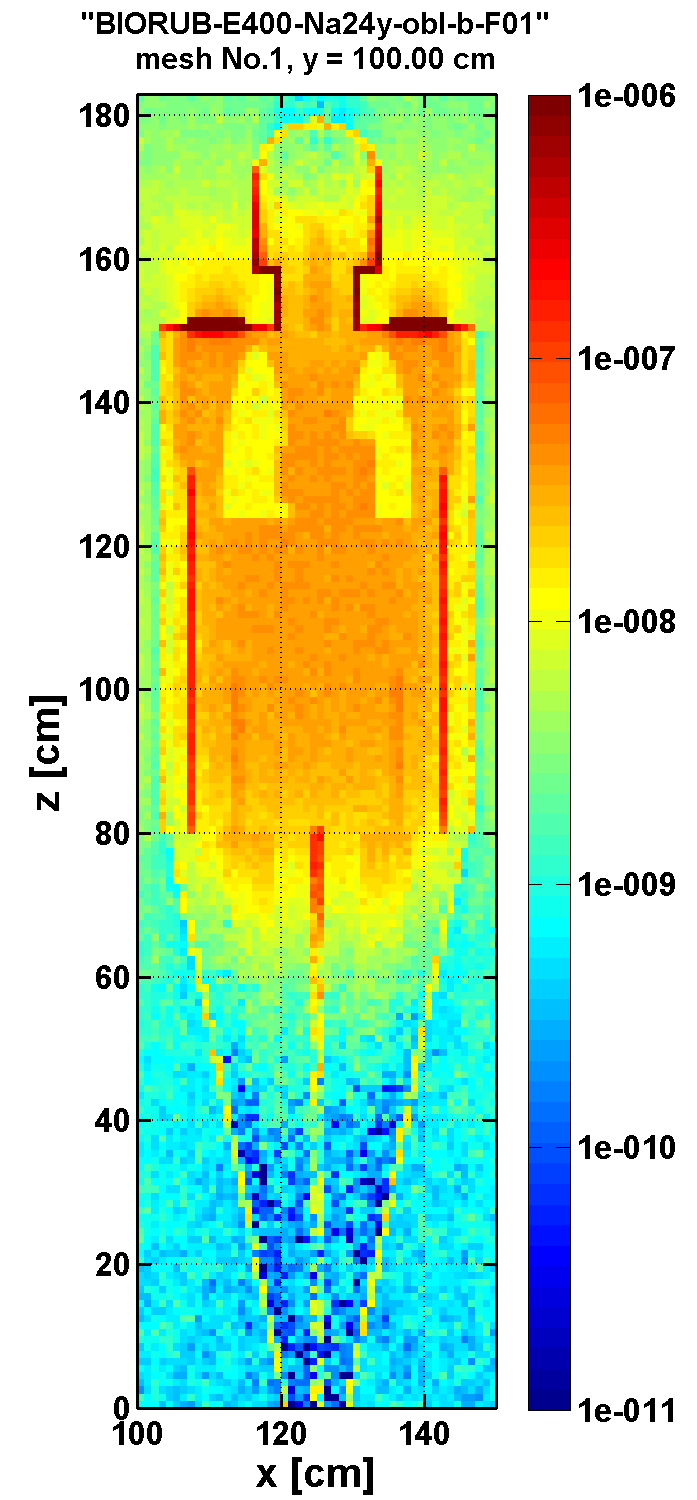

Supplement: Supplementary Materials — The electronic annex presents (1) numerical decrease of the various organs' contribution to the effective dose when protected with individual tested PPE exposed to the other simulated radionuclides dispersed in 10 m3 of the atmosphere in the RAC geometry (Table 2) and (2) visualisations of simulated ORNL phantom energy depositions while only wearing PPE preventing radioactive contamination, and the same PPE together with individual PPE protecting against X- and gamma-ray under it, in a various-dispersed radionuclide's aerosol atmosphere. [file 1641895.f1.zip › 1641895.f1/Electronic annex/Visualization of 2D distributions/Beta contribution/XZ/XZ-b-ppe-Na24-BIORUB-E400.png]

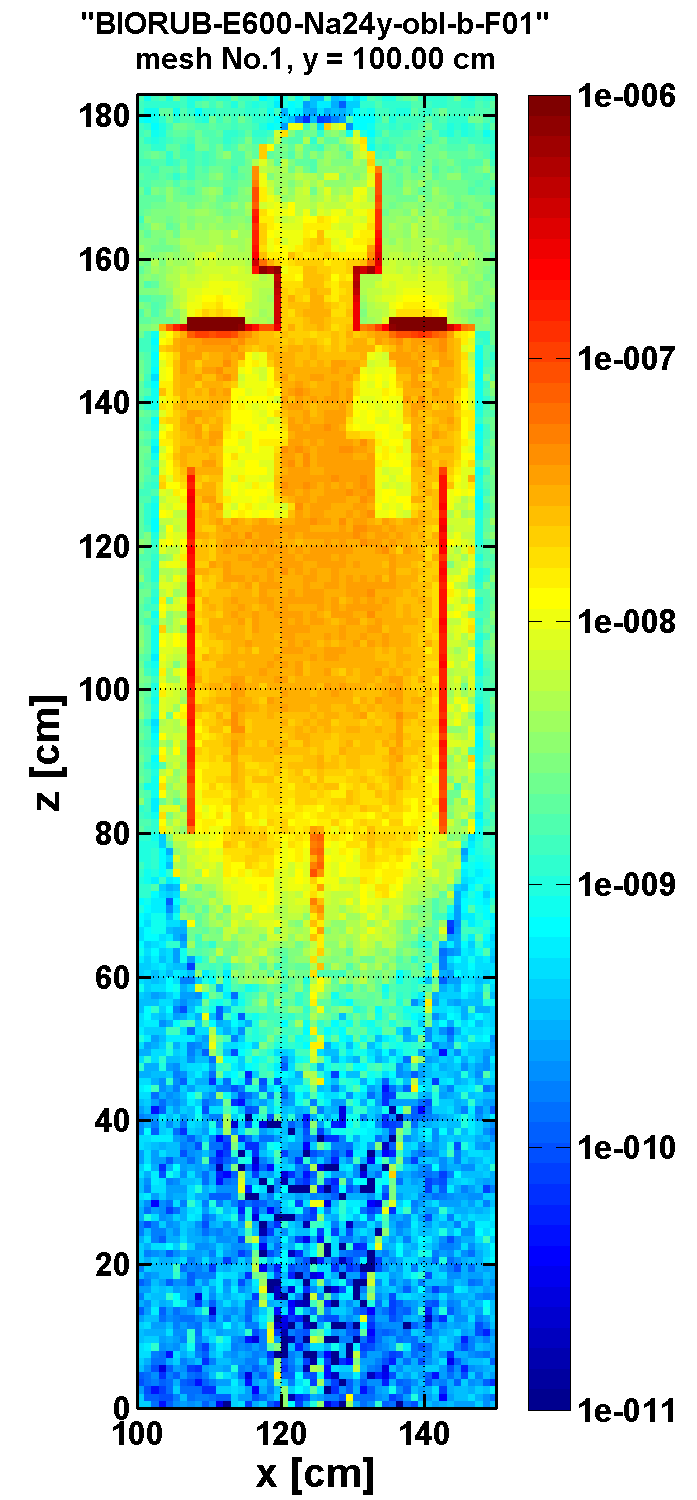

Supplement: Supplementary Materials — The electronic annex presents (1) numerical decrease of the various organs' contribution to the effective dose when protected with individual tested PPE exposed to the other simulated radionuclides dispersed in 10 m3 of the atmosphere in the RAC geometry (Table 2) and (2) visualisations of simulated ORNL phantom energy depositions while only wearing PPE preventing radioactive contamination, and the same PPE together with individual PPE protecting against X- and gamma-ray under it, in a various-dispersed radionuclide's aerosol atmosphere. [file 1641895.f1.zip › 1641895.f1/Electronic annex/Visualization of 2D distributions/Beta contribution/XZ/XZ-b-ppe-Na24-BIORUB-E600.png]

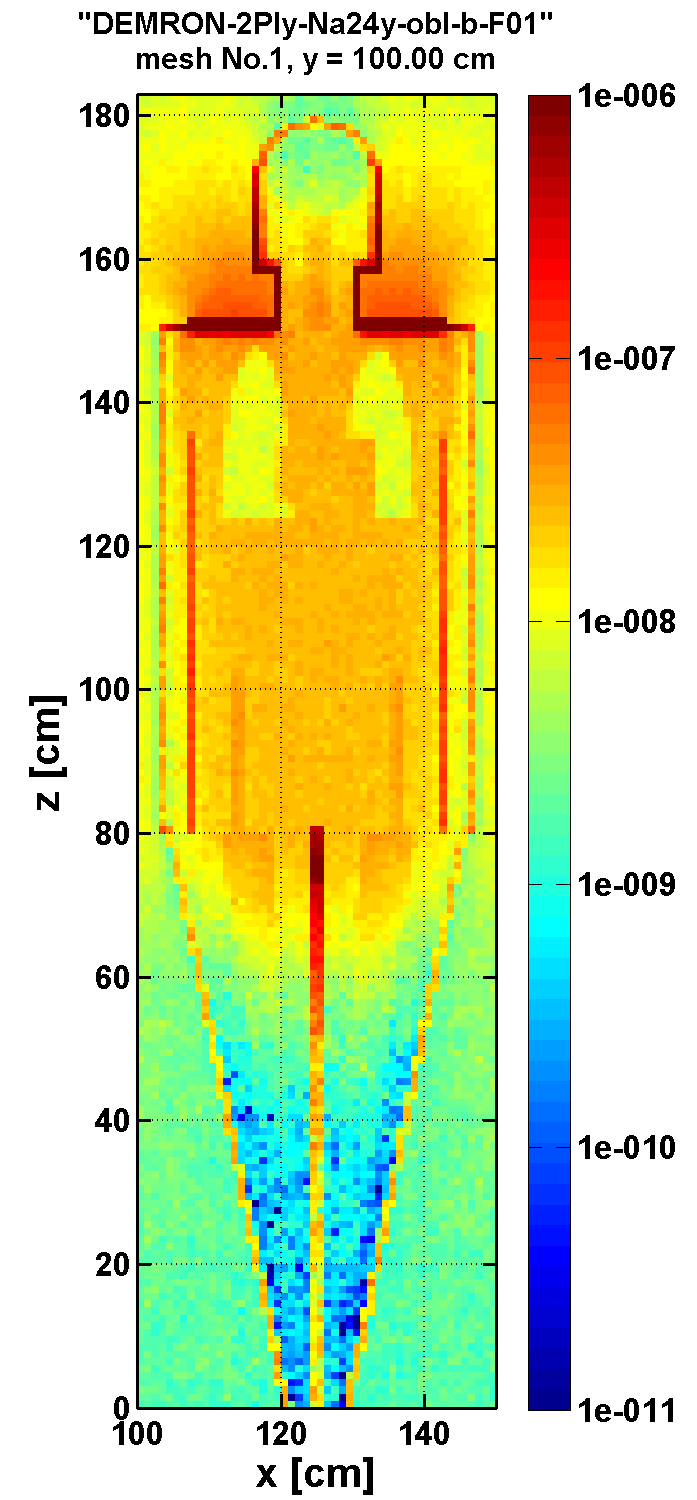

Supplement: Supplementary Materials — The electronic annex presents (1) numerical decrease of the various organs' contribution to the effective dose when protected with individual tested PPE exposed to the other simulated radionuclides dispersed in 10 m3 of the atmosphere in the RAC geometry (Table 2) and (2) visualisations of simulated ORNL phantom energy depositions while only wearing PPE preventing radioactive contamination, and the same PPE together with individual PPE protecting against X- and gamma-ray under it, in a various-dispersed radionuclide's aerosol atmosphere. [file 1641895.f1.zip › 1641895.f1/Electronic annex/Visualization of 2D distributions/Beta contribution/XZ/XZ-b-ppe-Na24-DEMRON-2Ply.png]

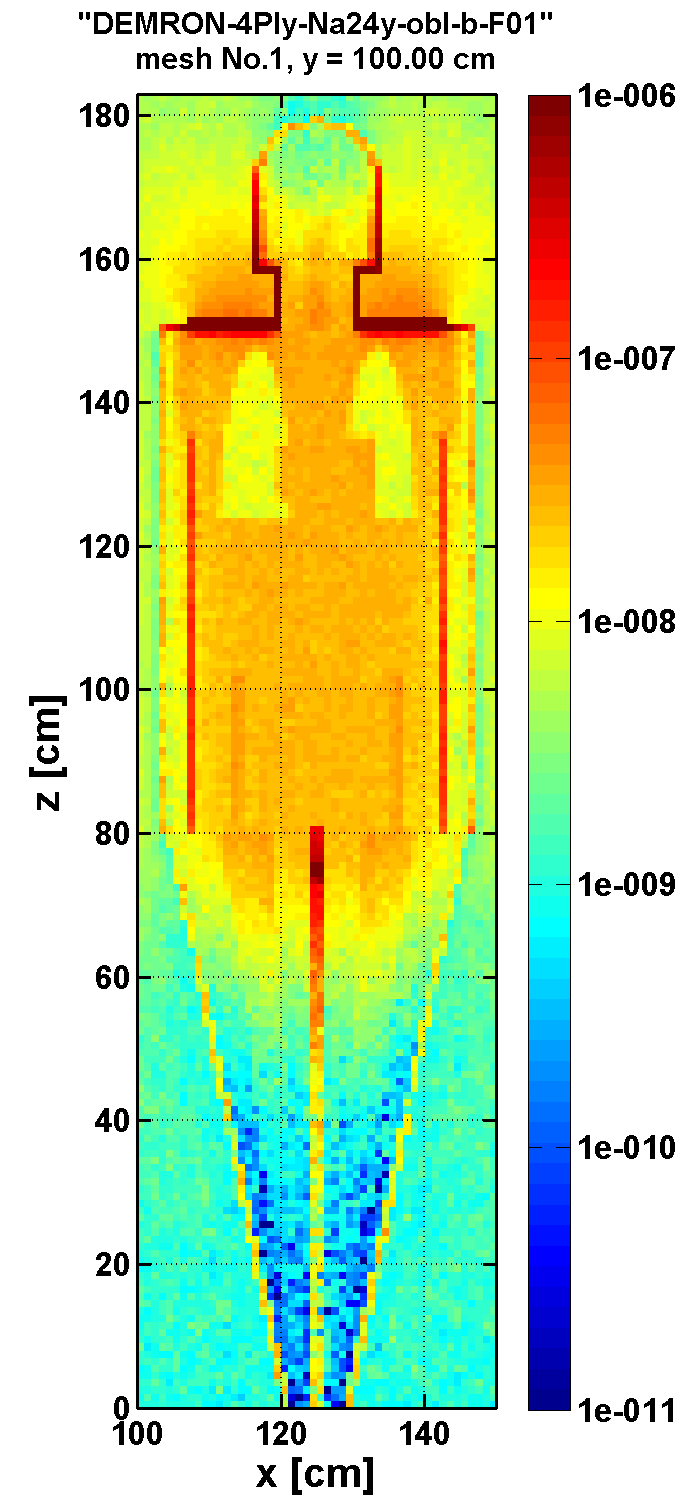

Supplement: Supplementary Materials — The electronic annex presents (1) numerical decrease of the various organs' contribution to the effective dose when protected with individual tested PPE exposed to the other simulated radionuclides dispersed in 10 m3 of the atmosphere in the RAC geometry (Table 2) and (2) visualisations of simulated ORNL phantom energy depositions while only wearing PPE preventing radioactive contamination, and the same PPE together with individual PPE protecting against X- and gamma-ray under it, in a various-dispersed radionuclide's aerosol atmosphere. [file 1641895.f1.zip › 1641895.f1/Electronic annex/Visualization of 2D distributions/Beta contribution/XZ/XZ-b-ppe-Na24-DEMRON-4Ply.png]

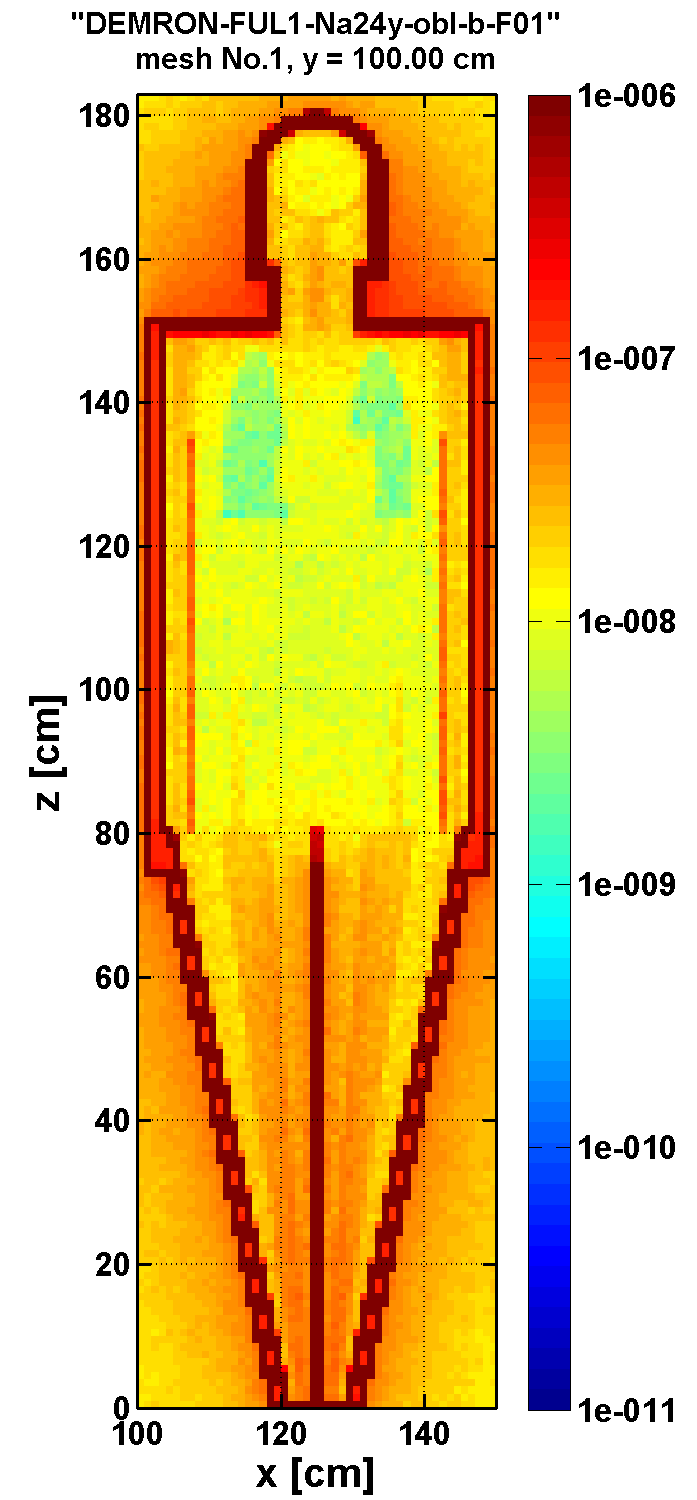

Supplement: Supplementary Materials — The electronic annex presents (1) numerical decrease of the various organs' contribution to the effective dose when protected with individual tested PPE exposed to the other simulated radionuclides dispersed in 10 m3 of the atmosphere in the RAC geometry (Table 2) and (2) visualisations of simulated ORNL phantom energy depositions while only wearing PPE preventing radioactive contamination, and the same PPE together with individual PPE protecting against X- and gamma-ray under it, in a various-dispersed radionuclide's aerosol atmosphere. [file 1641895.f1.zip › 1641895.f1/Electronic annex/Visualization of 2D distributions/Beta contribution/XZ/XZ-b-ppe-Na24-DEMRON-FUL1.png]

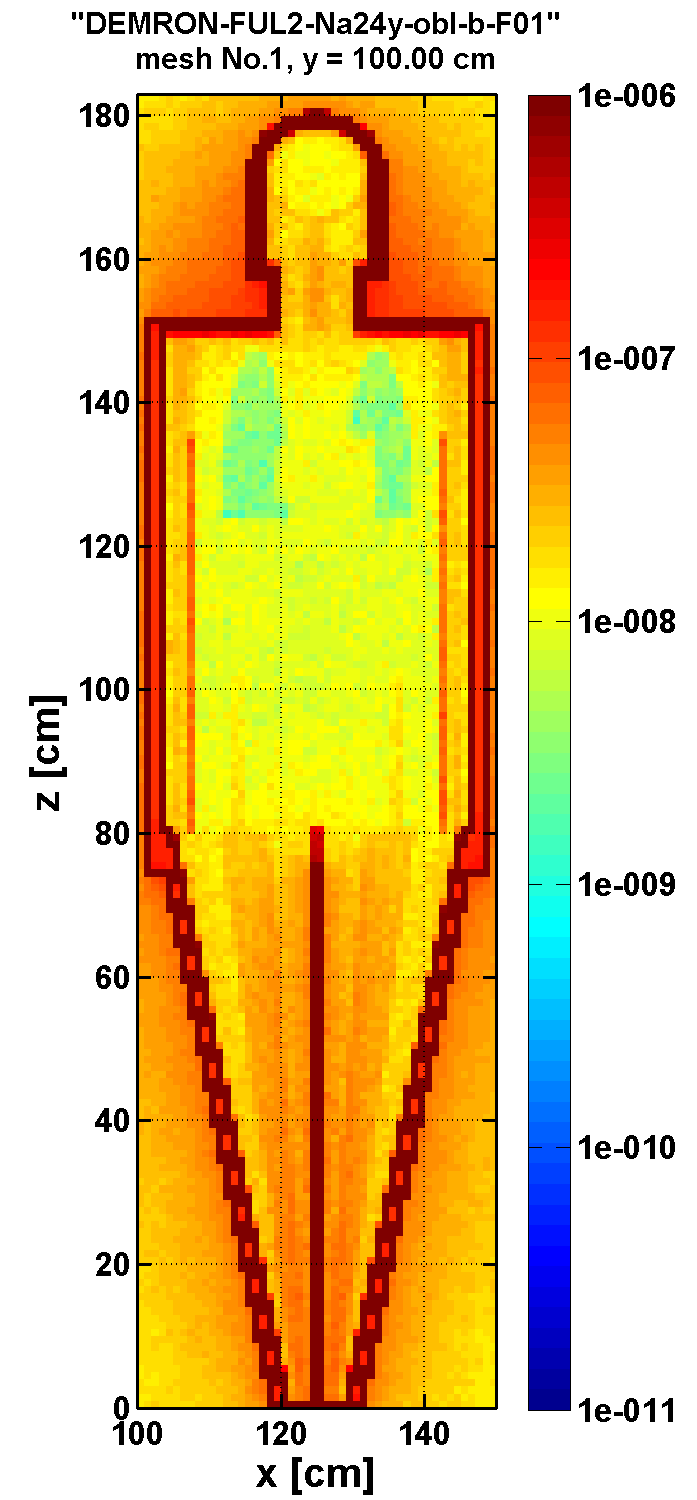

Supplement: Supplementary Materials — The electronic annex presents (1) numerical decrease of the various organs' contribution to the effective dose when protected with individual tested PPE exposed to the other simulated radionuclides dispersed in 10 m3 of the atmosphere in the RAC geometry (Table 2) and (2) visualisations of simulated ORNL phantom energy depositions while only wearing PPE preventing radioactive contamination, and the same PPE together with individual PPE protecting against X- and gamma-ray under it, in a various-dispersed radionuclide's aerosol atmosphere. [file 1641895.f1.zip › 1641895.f1/Electronic annex/Visualization of 2D distributions/Beta contribution/XZ/XZ-b-ppe-Na24-DEMRON-FUL2.png]

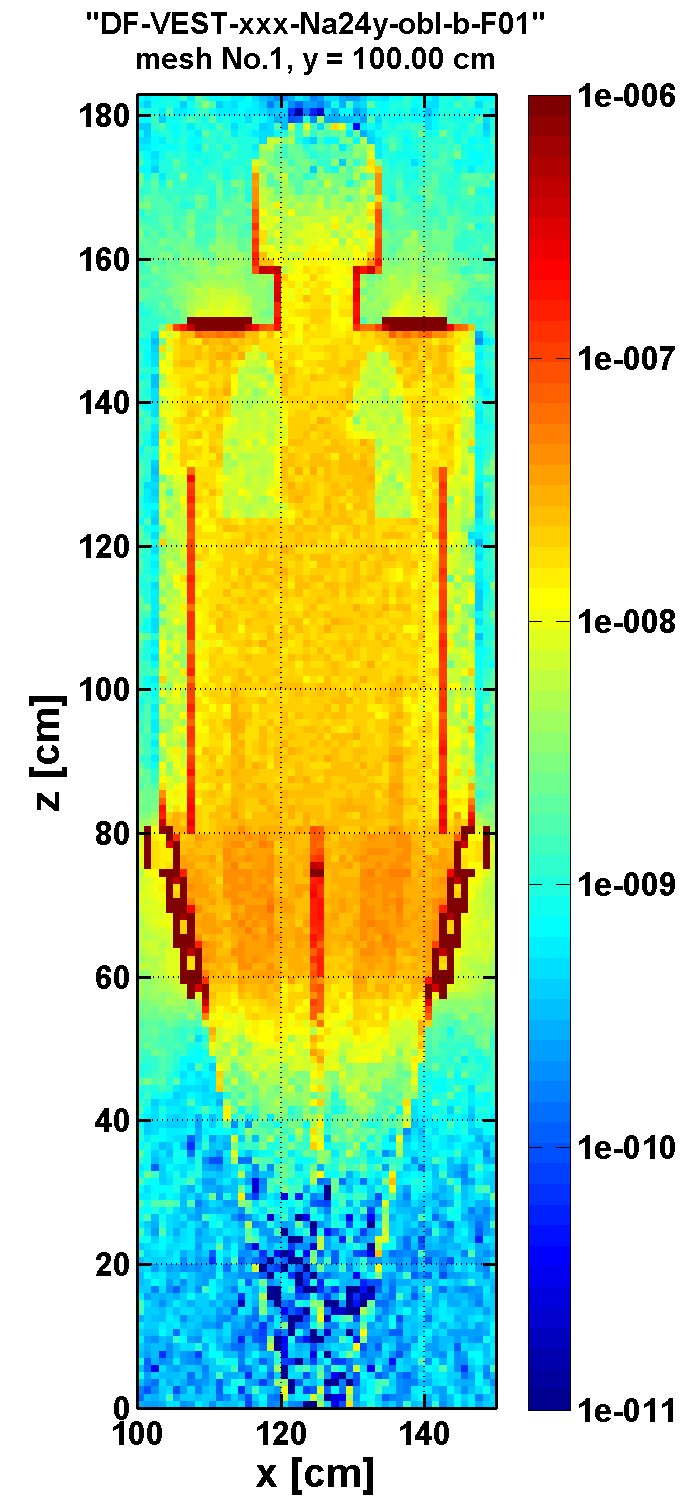

Supplement: Supplementary Materials — The electronic annex presents (1) numerical decrease of the various organs' contribution to the effective dose when protected with individual tested PPE exposed to the other simulated radionuclides dispersed in 10 m3 of the atmosphere in the RAC geometry (Table 2) and (2) visualisations of simulated ORNL phantom energy depositions while only wearing PPE preventing radioactive contamination, and the same PPE together with individual PPE protecting against X- and gamma-ray under it, in a various-dispersed radionuclide's aerosol atmosphere. [file 1641895.f1.zip › 1641895.f1/Electronic annex/Visualization of 2D distributions/Beta contribution/XZ/XZ-b-ppe-Na24-DF-VEST.png]

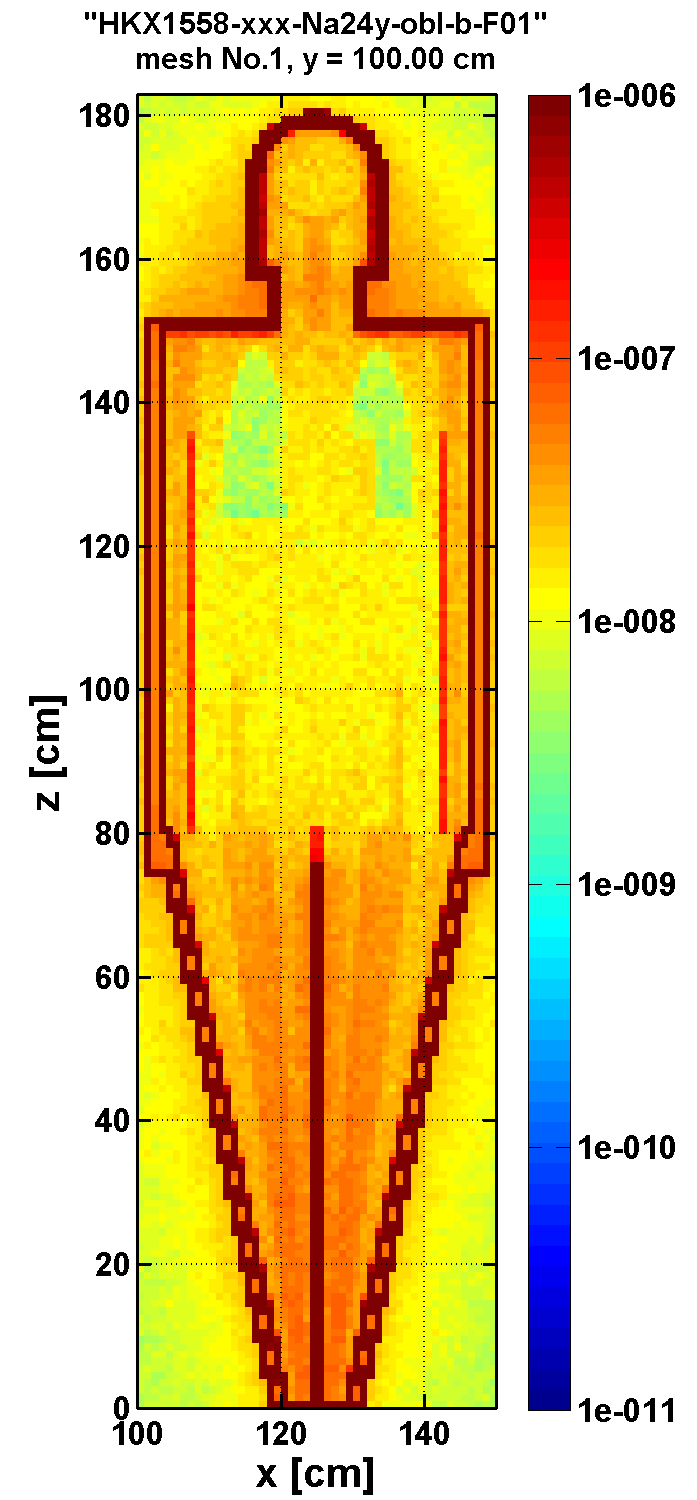

Supplement: Supplementary Materials — The electronic annex presents (1) numerical decrease of the various organs' contribution to the effective dose when protected with individual tested PPE exposed to the other simulated radionuclides dispersed in 10 m3 of the atmosphere in the RAC geometry (Table 2) and (2) visualisations of simulated ORNL phantom energy depositions while only wearing PPE preventing radioactive contamination, and the same PPE together with individual PPE protecting against X- and gamma-ray under it, in a various-dispersed radionuclide's aerosol atmosphere. [file 1641895.f1.zip › 1641895.f1/Electronic annex/Visualization of 2D distributions/Beta contribution/XZ/XZ-b-ppe-Na24-HKX1558.png]

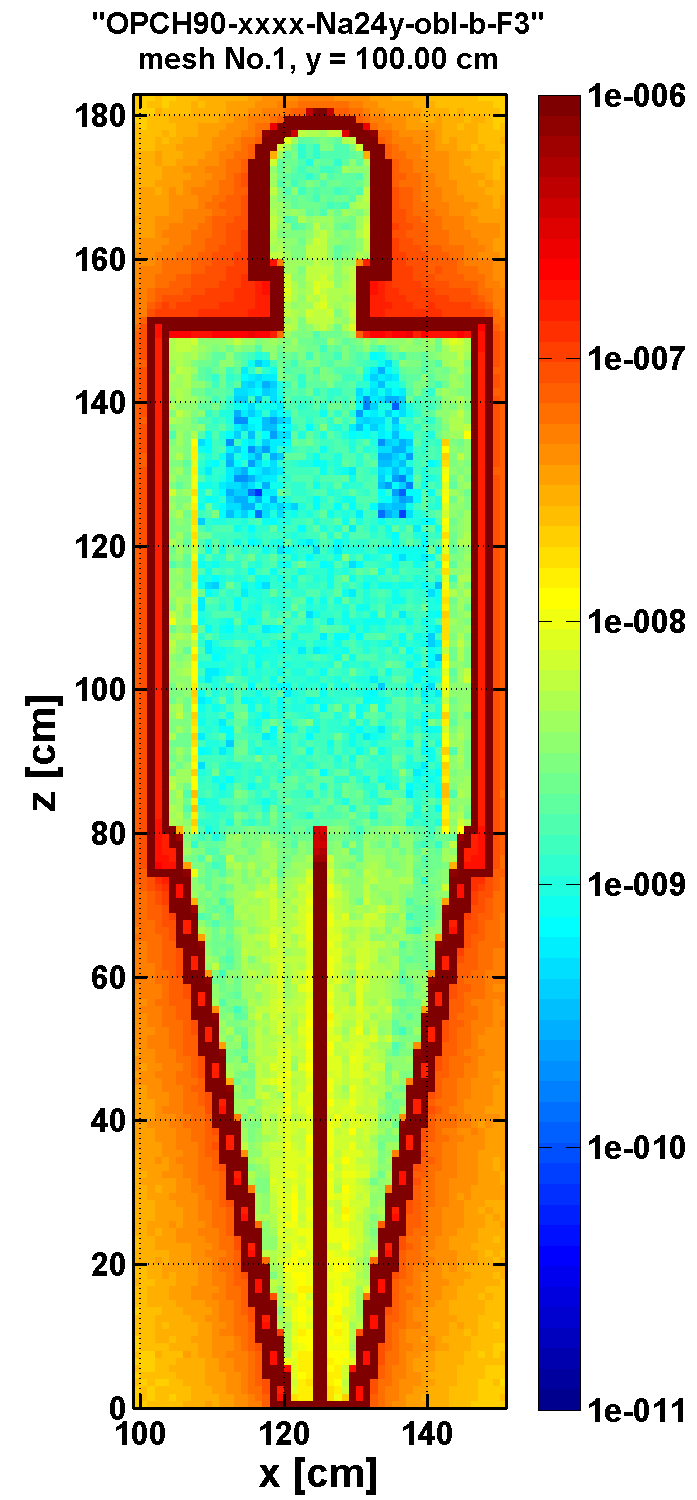

Supplement: Supplementary Materials — The electronic annex presents (1) numerical decrease of the various organs' contribution to the effective dose when protected with individual tested PPE exposed to the other simulated radionuclides dispersed in 10 m3 of the atmosphere in the RAC geometry (Table 2) and (2) visualisations of simulated ORNL phantom energy depositions while only wearing PPE preventing radioactive contamination, and the same PPE together with individual PPE protecting against X- and gamma-ray under it, in a various-dispersed radionuclide's aerosol atmosphere. [file 1641895.f1.zip › 1641895.f1/Electronic annex/Visualization of 2D distributions/Beta contribution/XZ/XZ-b-ppe-Na24-OPCH90.png]

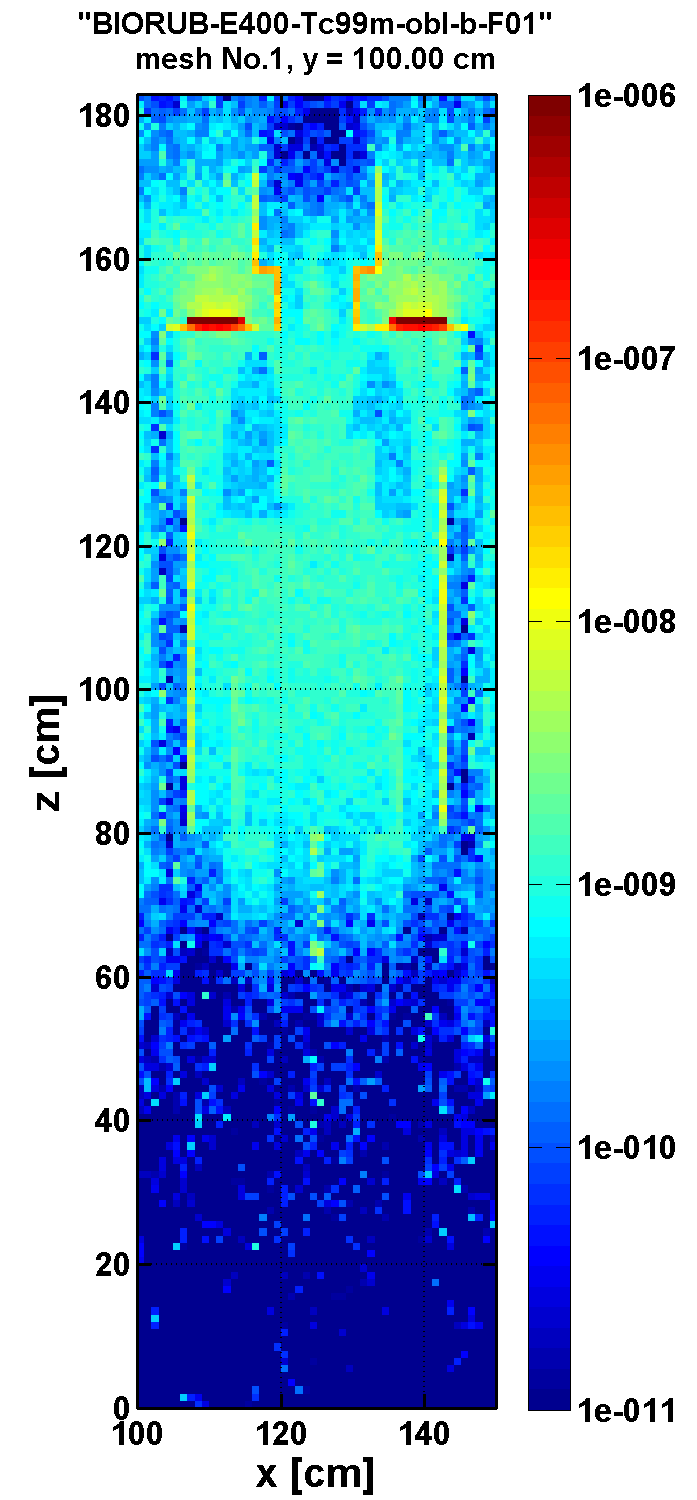

Supplement: Supplementary Materials — The electronic annex presents (1) numerical decrease of the various organs' contribution to the effective dose when protected with individual tested PPE exposed to the other simulated radionuclides dispersed in 10 m3 of the atmosphere in the RAC geometry (Table 2) and (2) visualisations of simulated ORNL phantom energy depositions while only wearing PPE preventing radioactive contamination, and the same PPE together with individual PPE protecting against X- and gamma-ray under it, in a various-dispersed radionuclide's aerosol atmosphere. [file 1641895.f1.zip › 1641895.f1/Electronic annex/Visualization of 2D distributions/Beta contribution/XZ/XZ-b-ppe-Tc99m-BIORUB-E400.png]

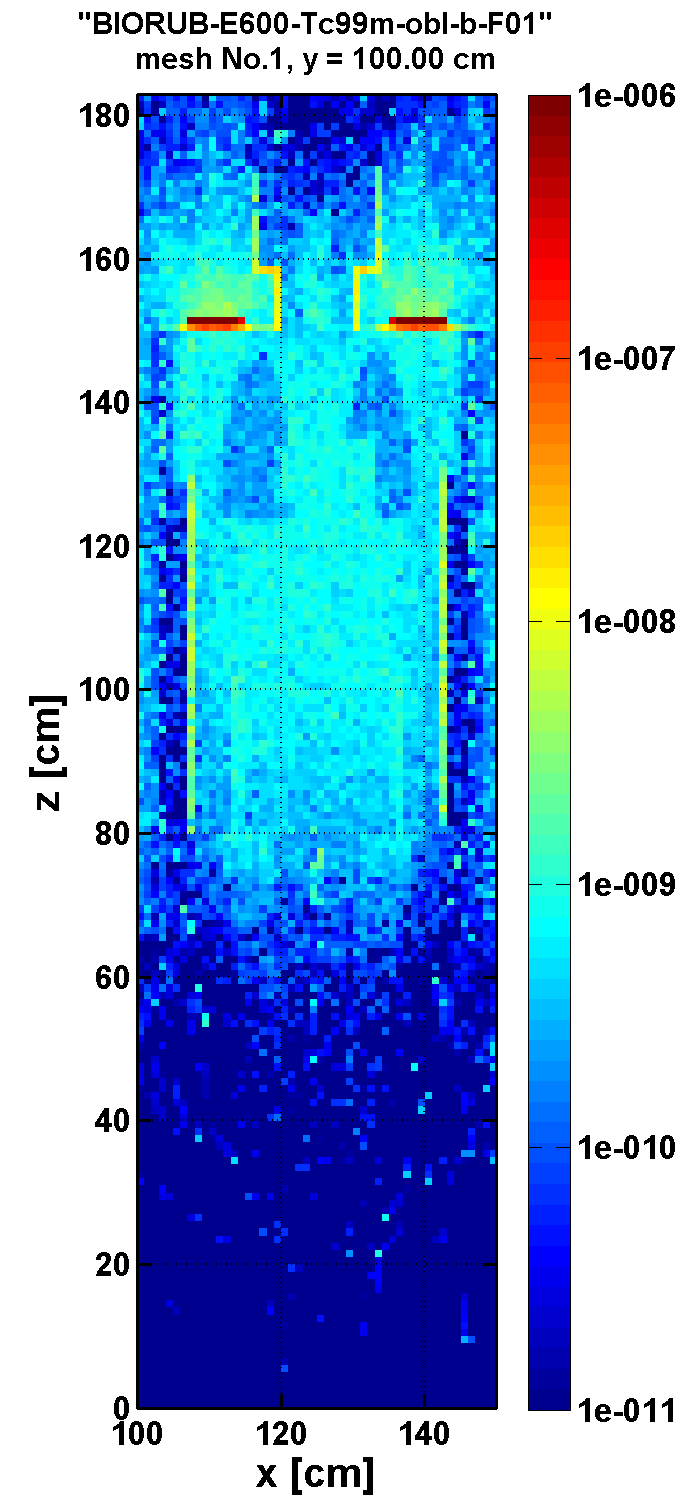

Supplement: Supplementary Materials — The electronic annex presents (1) numerical decrease of the various organs' contribution to the effective dose when protected with individual tested PPE exposed to the other simulated radionuclides dispersed in 10 m3 of the atmosphere in the RAC geometry (Table 2) and (2) visualisations of simulated ORNL phantom energy depositions while only wearing PPE preventing radioactive contamination, and the same PPE together with individual PPE protecting against X- and gamma-ray under it, in a various-dispersed radionuclide's aerosol atmosphere. [file 1641895.f1.zip › 1641895.f1/Electronic annex/Visualization of 2D distributions/Beta contribution/XZ/XZ-b-ppe-Tc99m-BIORUB-E600.png]

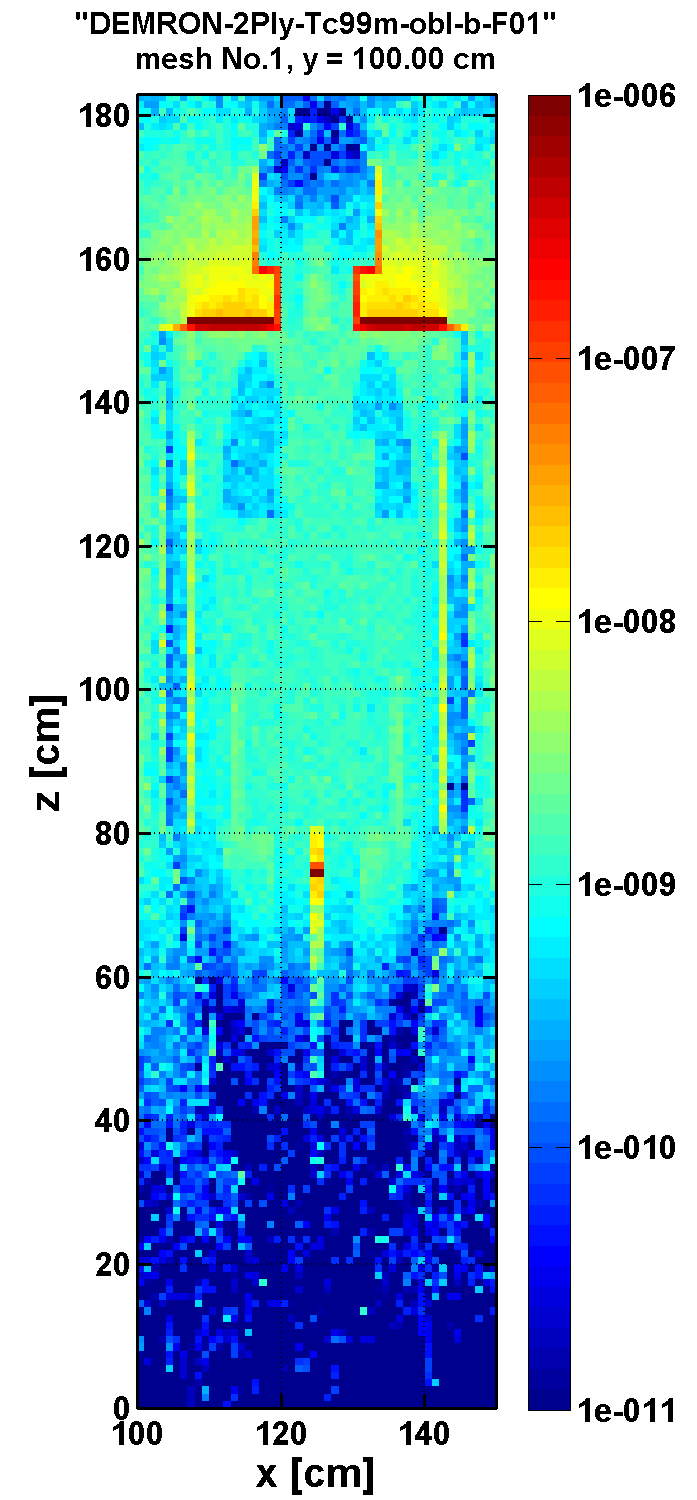

Supplement: Supplementary Materials — The electronic annex presents (1) numerical decrease of the various organs' contribution to the effective dose when protected with individual tested PPE exposed to the other simulated radionuclides dispersed in 10 m3 of the atmosphere in the RAC geometry (Table 2) and (2) visualisations of simulated ORNL phantom energy depositions while only wearing PPE preventing radioactive contamination, and the same PPE together with individual PPE protecting against X- and gamma-ray under it, in a various-dispersed radionuclide's aerosol atmosphere. [file 1641895.f1.zip › 1641895.f1/Electronic annex/Visualization of 2D distributions/Beta contribution/XZ/XZ-b-ppe-Tc99m-DEMRON-2Ply.png]

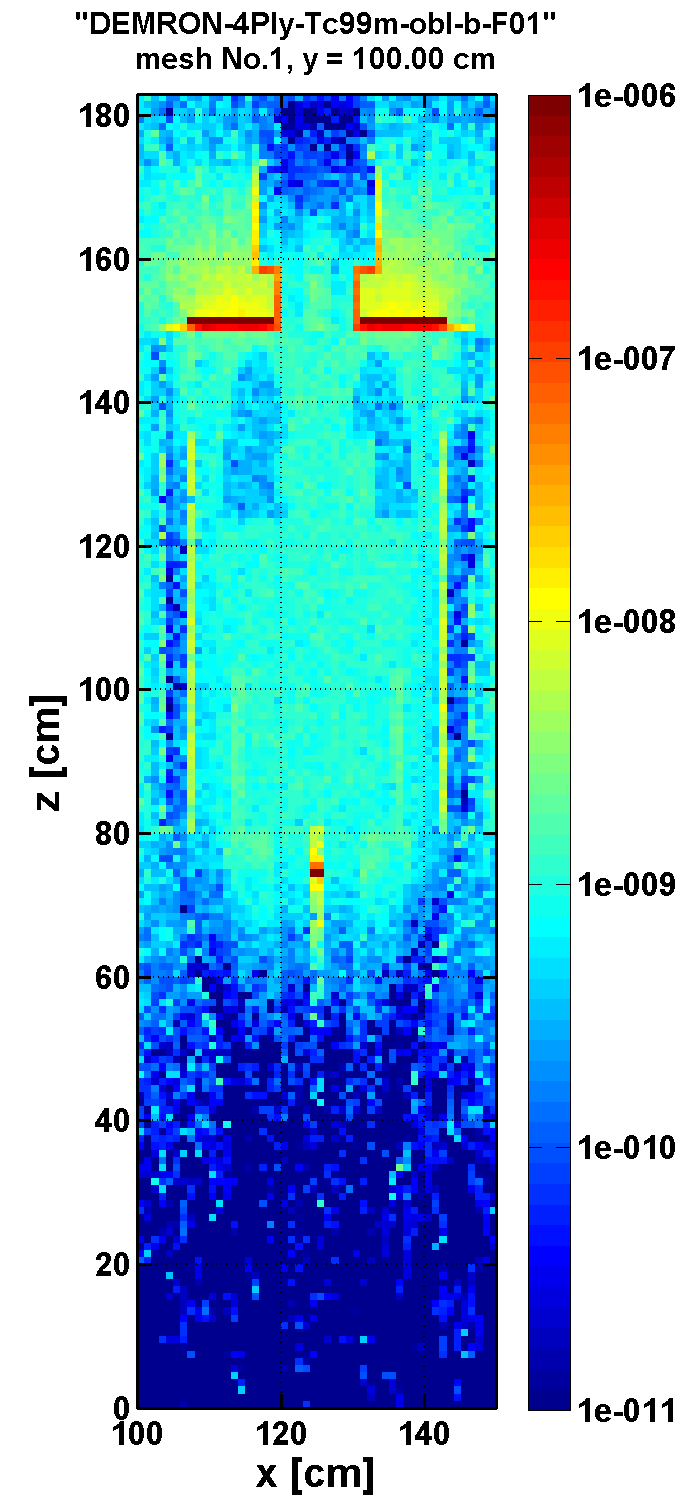

Supplement: Supplementary Materials — The electronic annex presents (1) numerical decrease of the various organs' contribution to the effective dose when protected with individual tested PPE exposed to the other simulated radionuclides dispersed in 10 m3 of the atmosphere in the RAC geometry (Table 2) and (2) visualisations of simulated ORNL phantom energy depositions while only wearing PPE preventing radioactive contamination, and the same PPE together with individual PPE protecting against X- and gamma-ray under it, in a various-dispersed radionuclide's aerosol atmosphere. [file 1641895.f1.zip › 1641895.f1/Electronic annex/Visualization of 2D distributions/Beta contribution/XZ/XZ-b-ppe-Tc99m-DEMRON-4Ply.png]

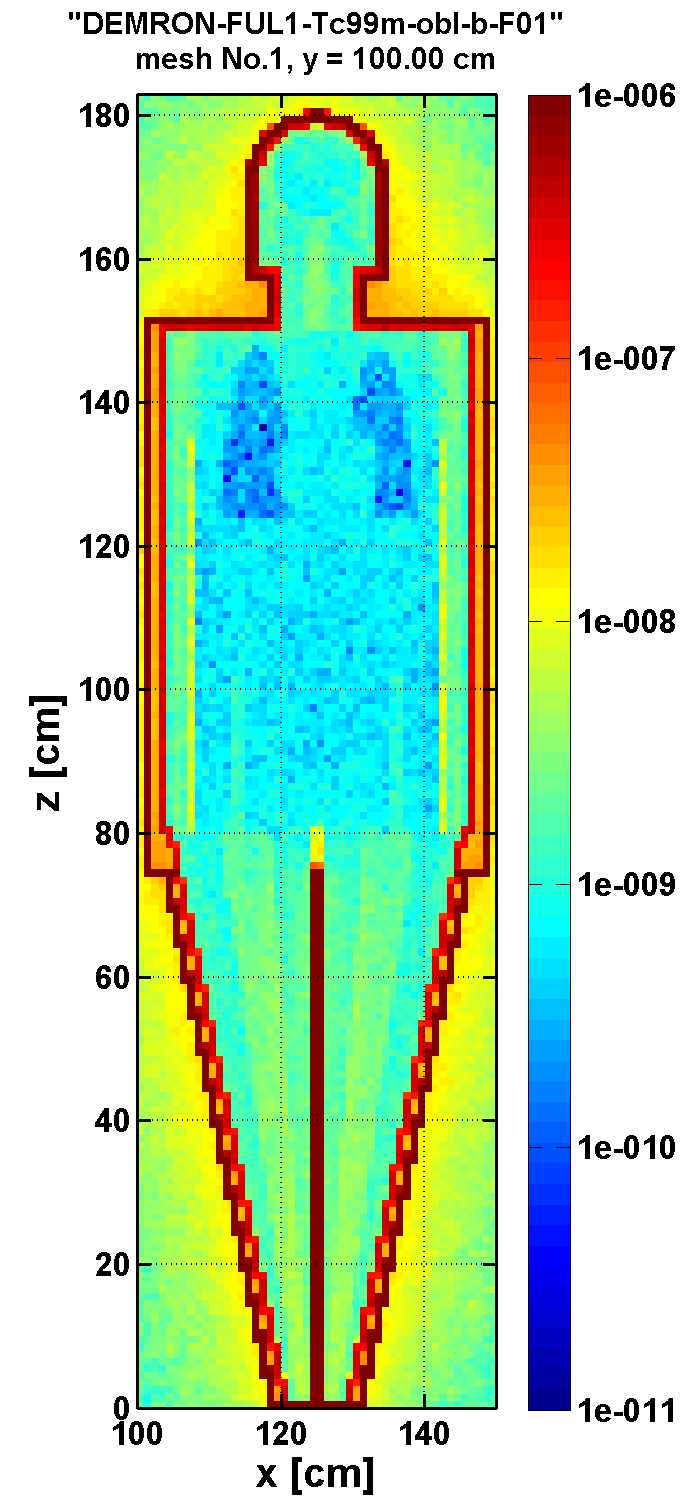

Supplement: Supplementary Materials — The electronic annex presents (1) numerical decrease of the various organs' contribution to the effective dose when protected with individual tested PPE exposed to the other simulated radionuclides dispersed in 10 m3 of the atmosphere in the RAC geometry (Table 2) and (2) visualisations of simulated ORNL phantom energy depositions while only wearing PPE preventing radioactive contamination, and the same PPE together with individual PPE protecting against X- and gamma-ray under it, in a various-dispersed radionuclide's aerosol atmosphere. [file 1641895.f1.zip › 1641895.f1/Electronic annex/Visualization of 2D distributions/Beta contribution/XZ/XZ-b-ppe-Tc99m-DEMRON-FUL1.png]

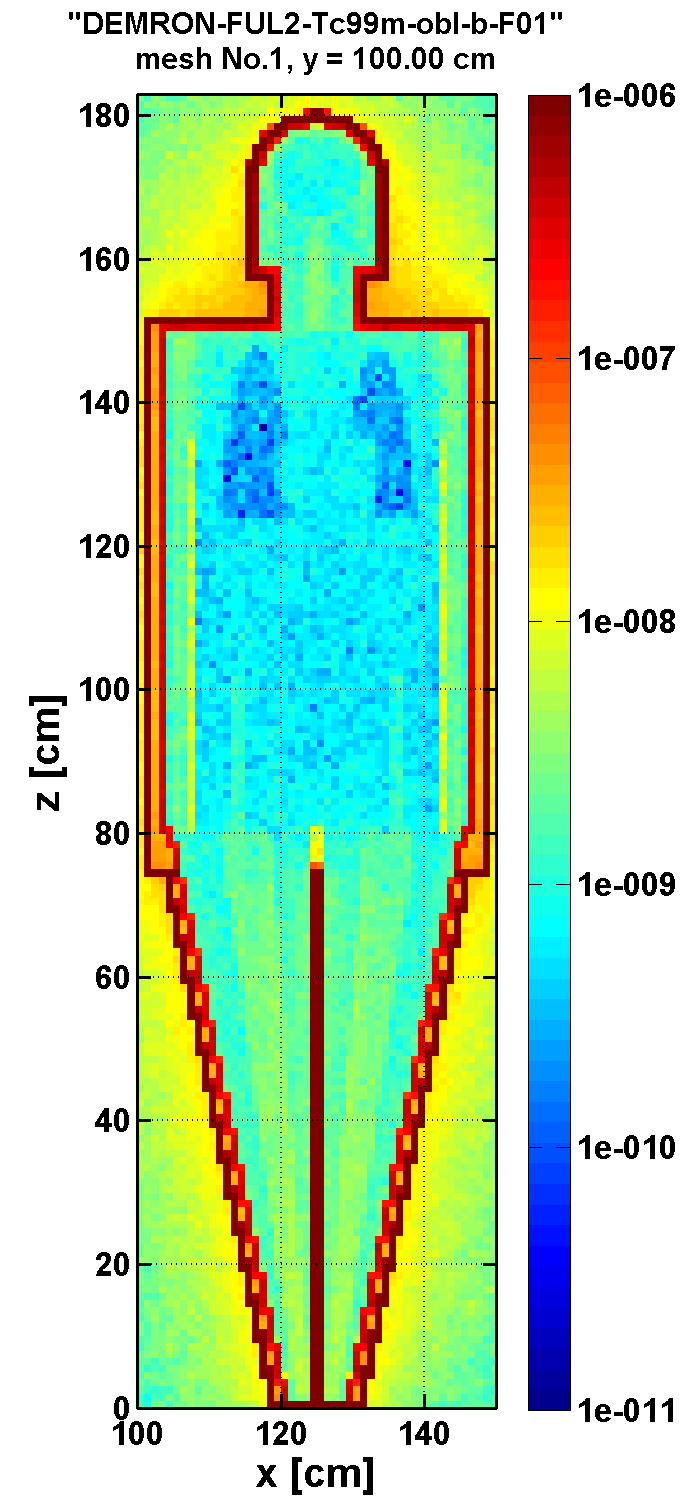

Supplement: Supplementary Materials — The electronic annex presents (1) numerical decrease of the various organs' contribution to the effective dose when protected with individual tested PPE exposed to the other simulated radionuclides dispersed in 10 m3 of the atmosphere in the RAC geometry (Table 2) and (2) visualisations of simulated ORNL phantom energy depositions while only wearing PPE preventing radioactive contamination, and the same PPE together with individual PPE protecting against X- and gamma-ray under it, in a various-dispersed radionuclide's aerosol atmosphere. [file 1641895.f1.zip › 1641895.f1/Electronic annex/Visualization of 2D distributions/Beta contribution/XZ/XZ-b-ppe-Tc99m-DEMRON-FUL2.png]

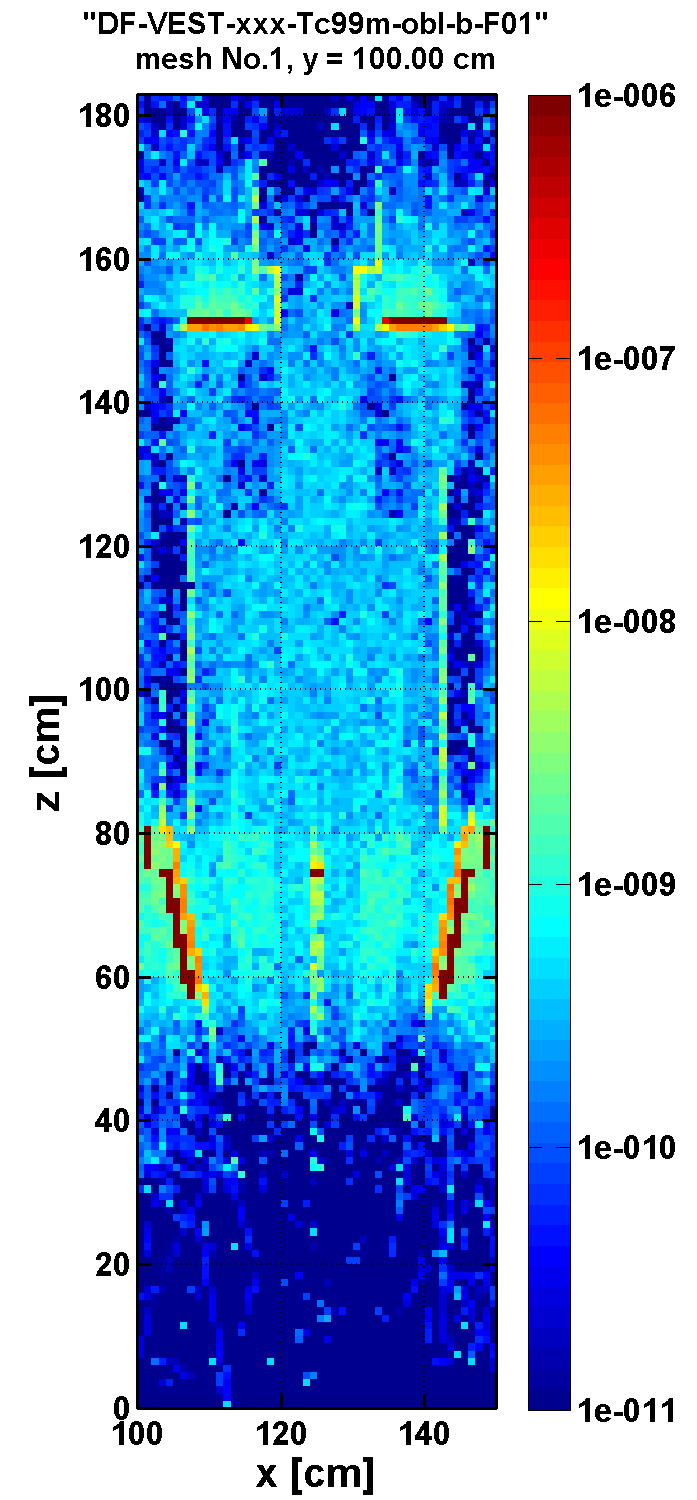

Supplement: Supplementary Materials — The electronic annex presents (1) numerical decrease of the various organs' contribution to the effective dose when protected with individual tested PPE exposed to the other simulated radionuclides dispersed in 10 m3 of the atmosphere in the RAC geometry (Table 2) and (2) visualisations of simulated ORNL phantom energy depositions while only wearing PPE preventing radioactive contamination, and the same PPE together with individual PPE protecting against X- and gamma-ray under it, in a various-dispersed radionuclide's aerosol atmosphere. [file 1641895.f1.zip › 1641895.f1/Electronic annex/Visualization of 2D distributions/Beta contribution/XZ/XZ-b-ppe-Tc99m-DF-VEST.png]

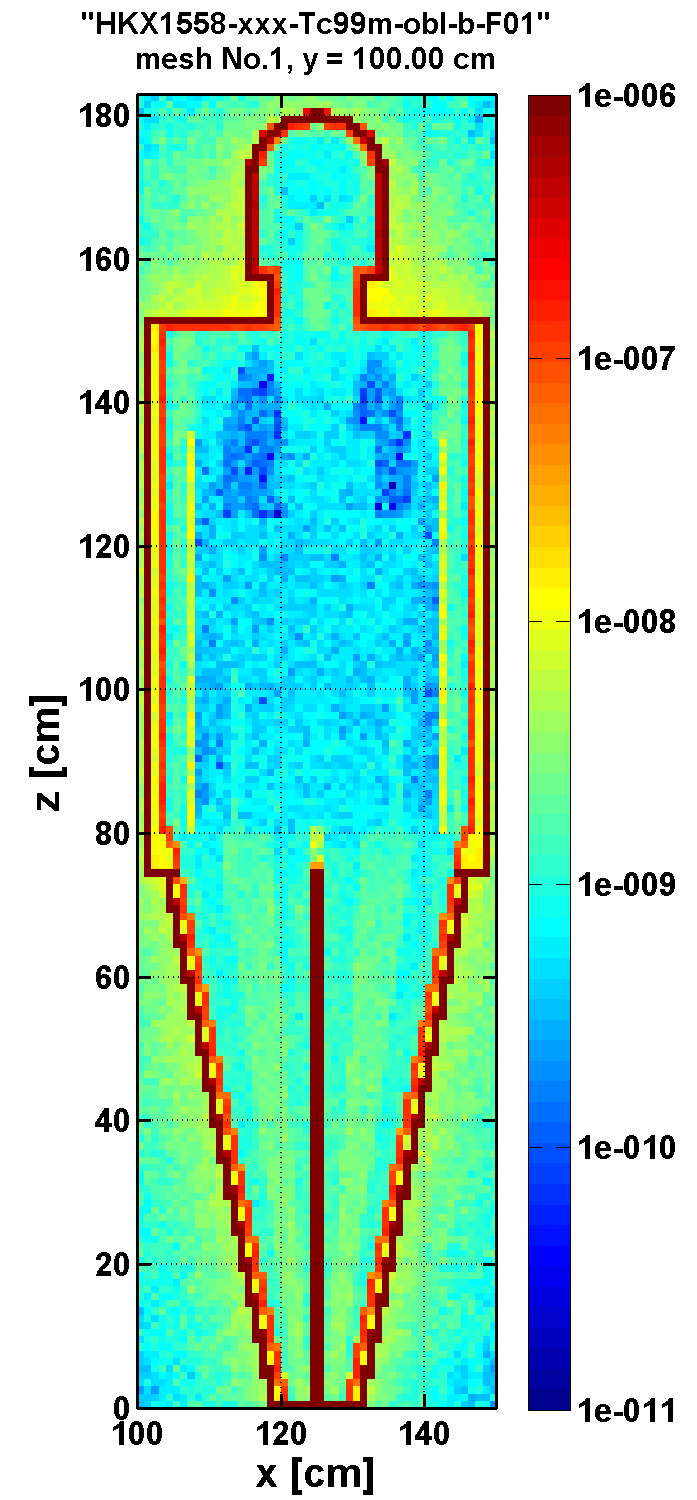

Supplement: Supplementary Materials — The electronic annex presents (1) numerical decrease of the various organs' contribution to the effective dose when protected with individual tested PPE exposed to the other simulated radionuclides dispersed in 10 m3 of the atmosphere in the RAC geometry (Table 2) and (2) visualisations of simulated ORNL phantom energy depositions while only wearing PPE preventing radioactive contamination, and the same PPE together with individual PPE protecting against X- and gamma-ray under it, in a various-dispersed radionuclide's aerosol atmosphere. [file 1641895.f1.zip › 1641895.f1/Electronic annex/Visualization of 2D distributions/Beta contribution/XZ/XZ-b-ppe-Tc99m-HKX1558.png]

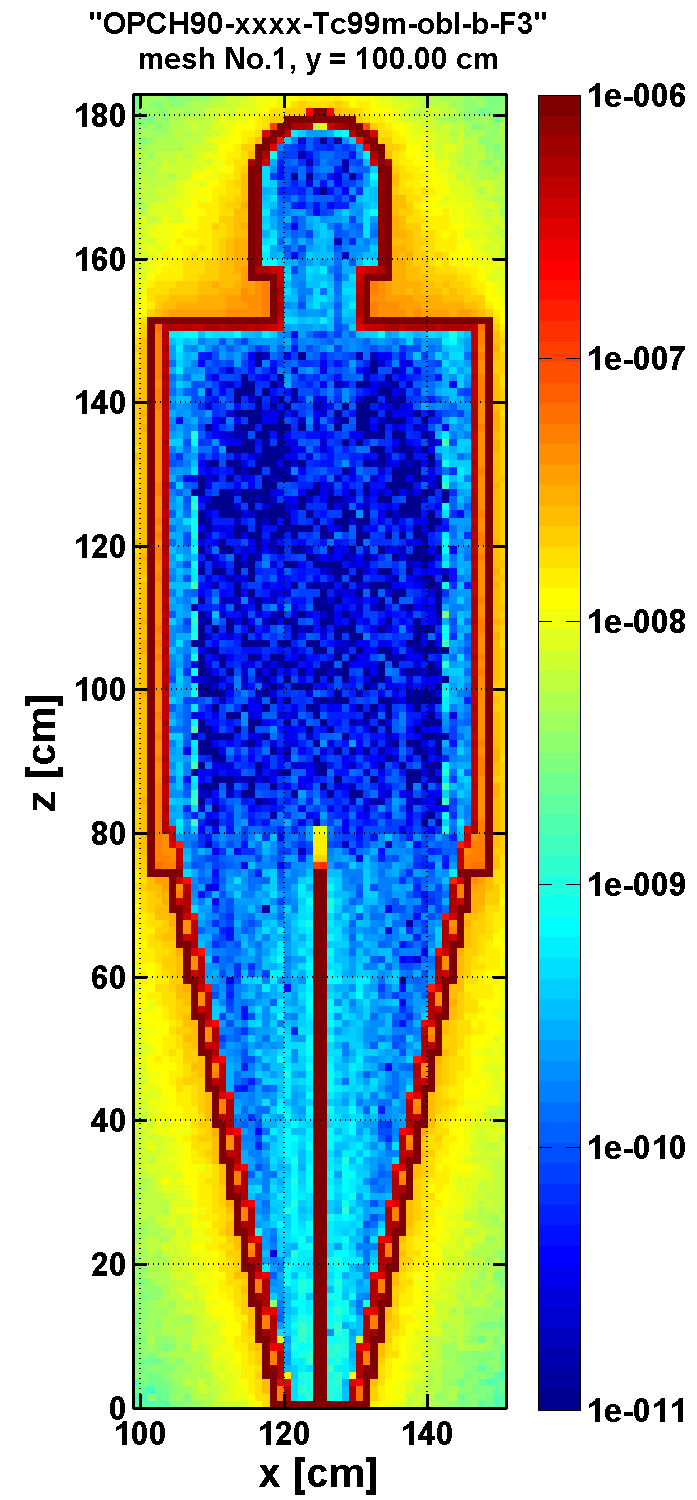

Supplement: Supplementary Materials — The electronic annex presents (1) numerical decrease of the various organs' contribution to the effective dose when protected with individual tested PPE exposed to the other simulated radionuclides dispersed in 10 m3 of the atmosphere in the RAC geometry (Table 2) and (2) visualisations of simulated ORNL phantom energy depositions while only wearing PPE preventing radioactive contamination, and the same PPE together with individual PPE protecting against X- and gamma-ray under it, in a various-dispersed radionuclide's aerosol atmosphere. [file 1641895.f1.zip › 1641895.f1/Electronic annex/Visualization of 2D distributions/Beta contribution/XZ/XZ-b-ppe-Tc99m-OPCH90.png]

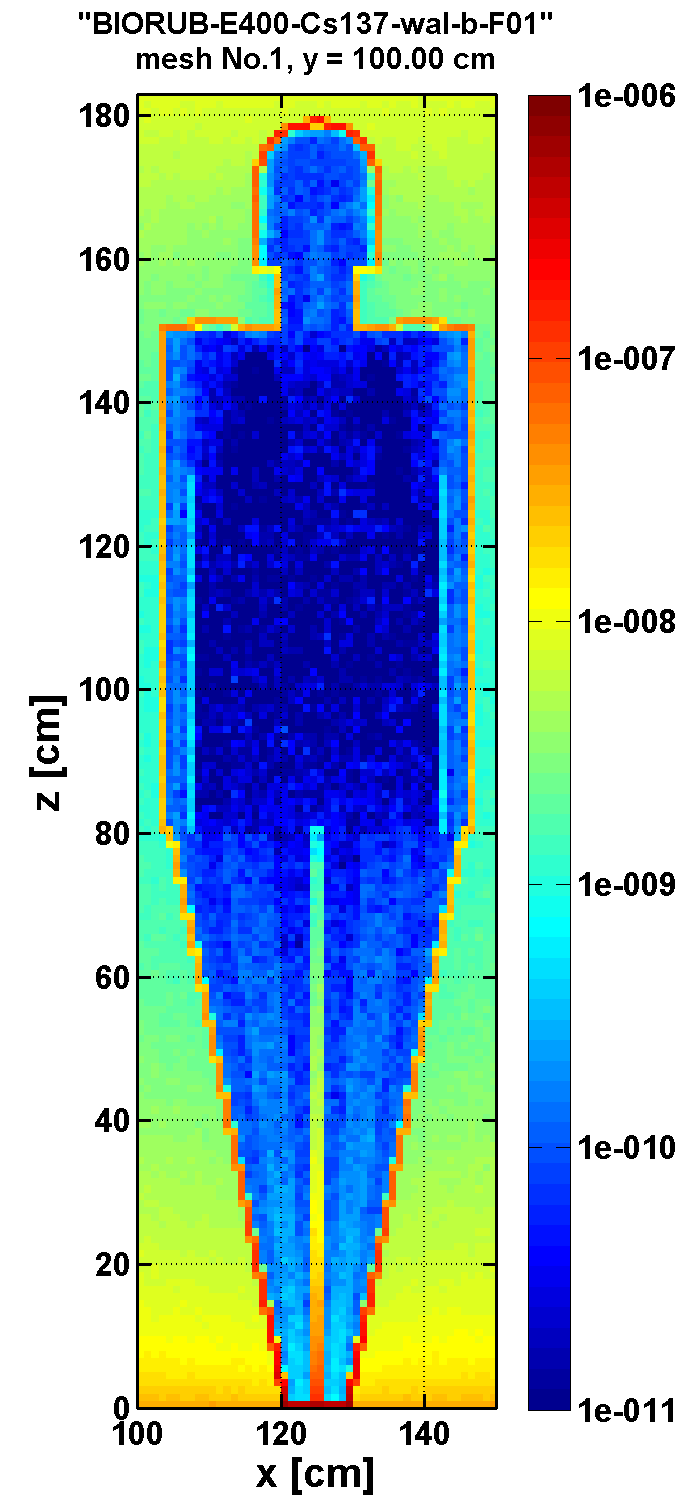

Supplement: Supplementary Materials — The electronic annex presents (1) numerical decrease of the various organs' contribution to the effective dose when protected with individual tested PPE exposed to the other simulated radionuclides dispersed in 10 m3 of the atmosphere in the RAC geometry (Table 2) and (2) visualisations of simulated ORNL phantom energy depositions while only wearing PPE preventing radioactive contamination, and the same PPE together with individual PPE protecting against X- and gamma-ray under it, in a various-dispersed radionuclide's aerosol atmosphere. [file 1641895.f1.zip › 1641895.f1/Electronic annex/Visualization of 2D distributions/Beta contribution/XZ/XZ-b-wal-Cs137-BIORUB-E400.png]

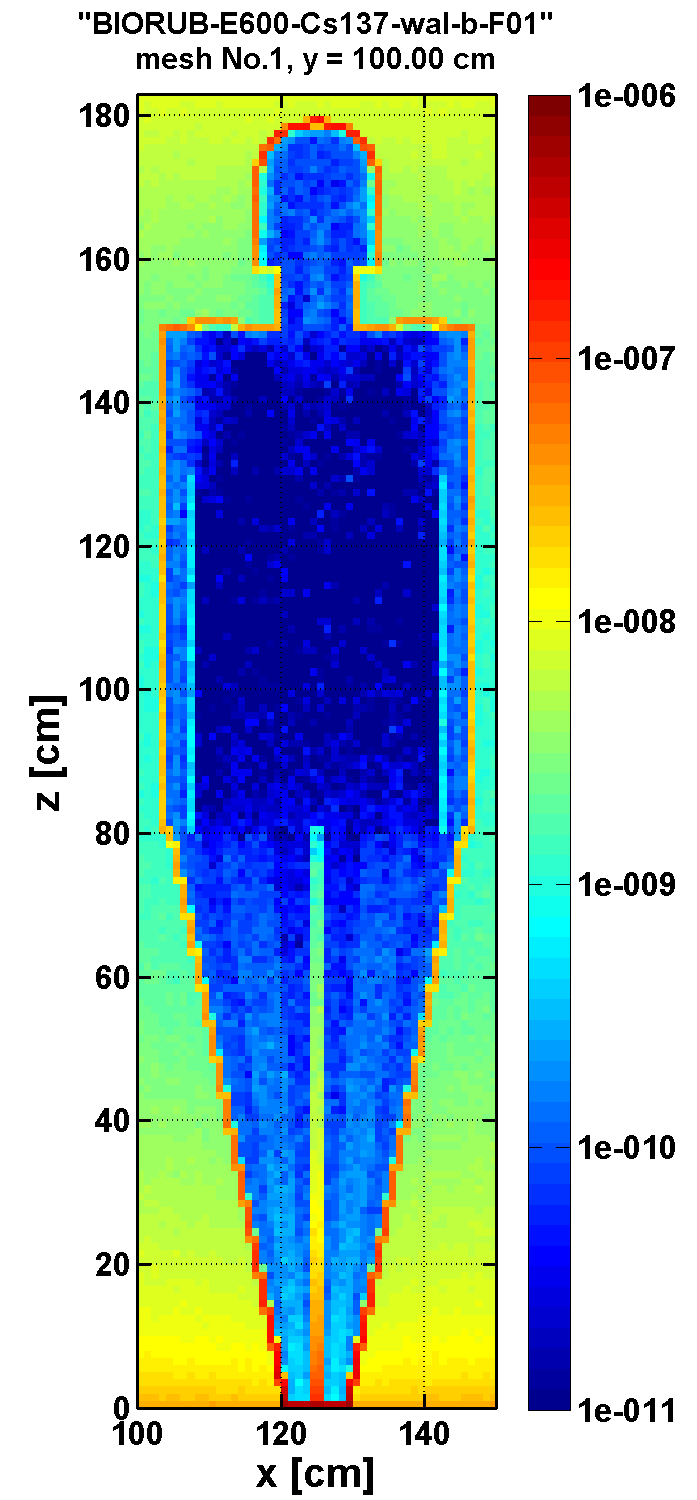

Supplement: Supplementary Materials — The electronic annex presents (1) numerical decrease of the various organs' contribution to the effective dose when protected with individual tested PPE exposed to the other simulated radionuclides dispersed in 10 m3 of the atmosphere in the RAC geometry (Table 2) and (2) visualisations of simulated ORNL phantom energy depositions while only wearing PPE preventing radioactive contamination, and the same PPE together with individual PPE protecting against X- and gamma-ray under it, in a various-dispersed radionuclide's aerosol atmosphere. [file 1641895.f1.zip › 1641895.f1/Electronic annex/Visualization of 2D distributions/Beta contribution/XZ/XZ-b-wal-Cs137-BIORUB-E600.png]

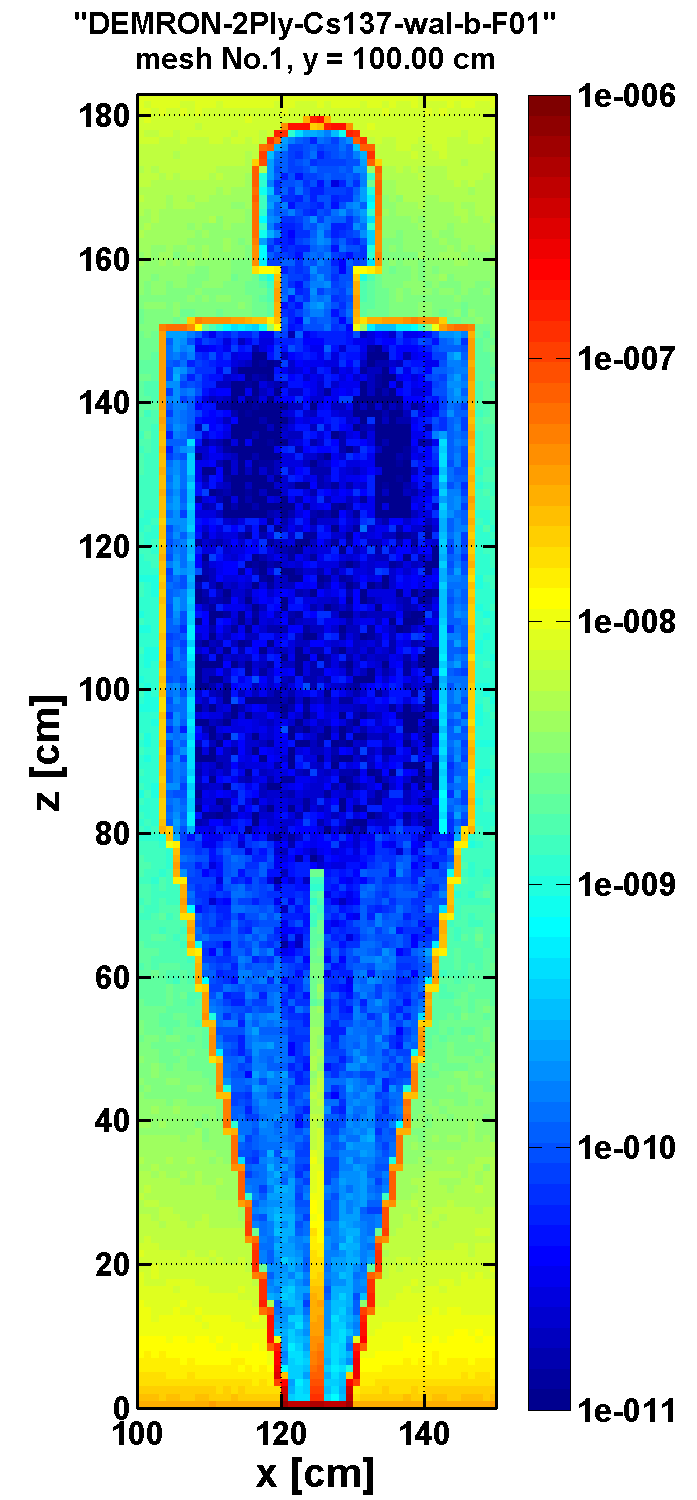

Supplement: Supplementary Materials — The electronic annex presents (1) numerical decrease of the various organs' contribution to the effective dose when protected with individual tested PPE exposed to the other simulated radionuclides dispersed in 10 m3 of the atmosphere in the RAC geometry (Table 2) and (2) visualisations of simulated ORNL phantom energy depositions while only wearing PPE preventing radioactive contamination, and the same PPE together with individual PPE protecting against X- and gamma-ray under it, in a various-dispersed radionuclide's aerosol atmosphere. [file 1641895.f1.zip › 1641895.f1/Electronic annex/Visualization of 2D distributions/Beta contribution/XZ/XZ-b-wal-Cs137-DEMRON-2Ply.png]

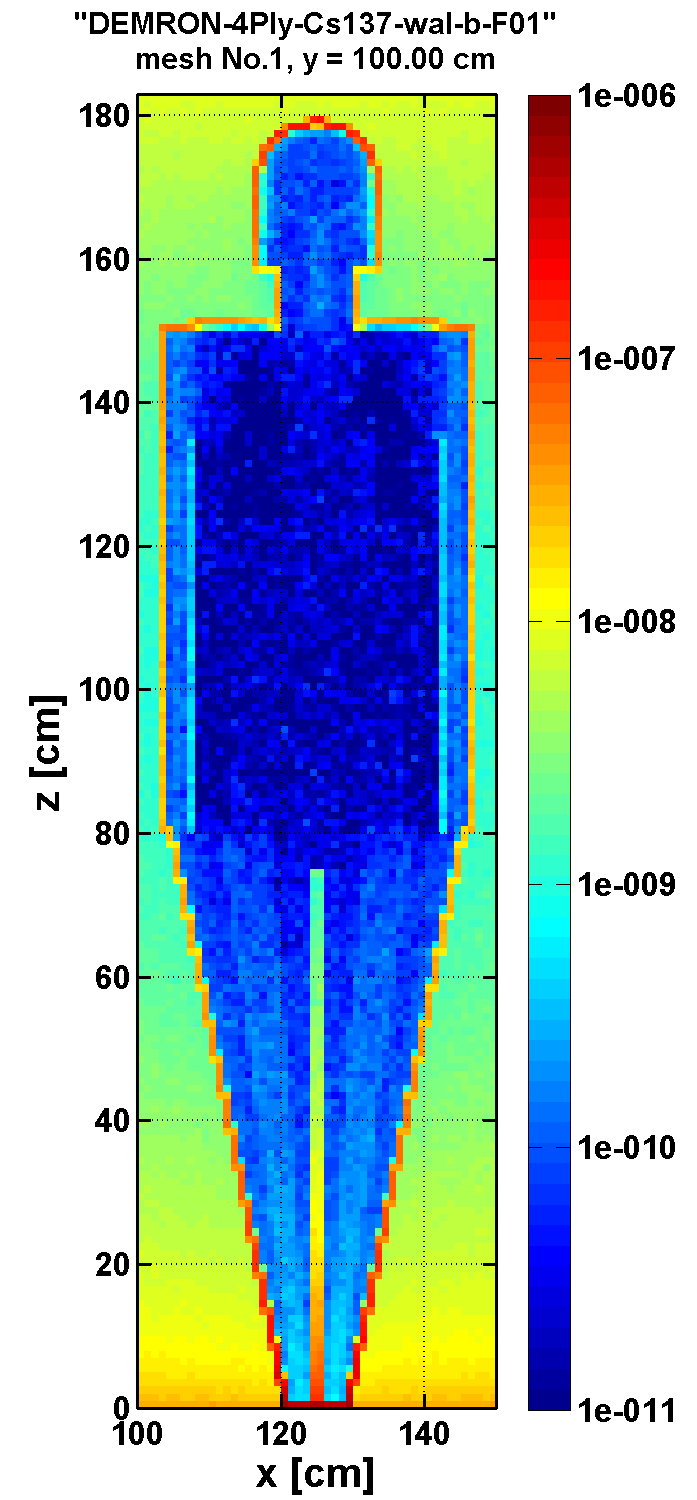

Supplement: Supplementary Materials — The electronic annex presents (1) numerical decrease of the various organs' contribution to the effective dose when protected with individual tested PPE exposed to the other simulated radionuclides dispersed in 10 m3 of the atmosphere in the RAC geometry (Table 2) and (2) visualisations of simulated ORNL phantom energy depositions while only wearing PPE preventing radioactive contamination, and the same PPE together with individual PPE protecting against X- and gamma-ray under it, in a various-dispersed radionuclide's aerosol atmosphere. [file 1641895.f1.zip › 1641895.f1/Electronic annex/Visualization of 2D distributions/Beta contribution/XZ/XZ-b-wal-Cs137-DEMRON-4Ply.png]

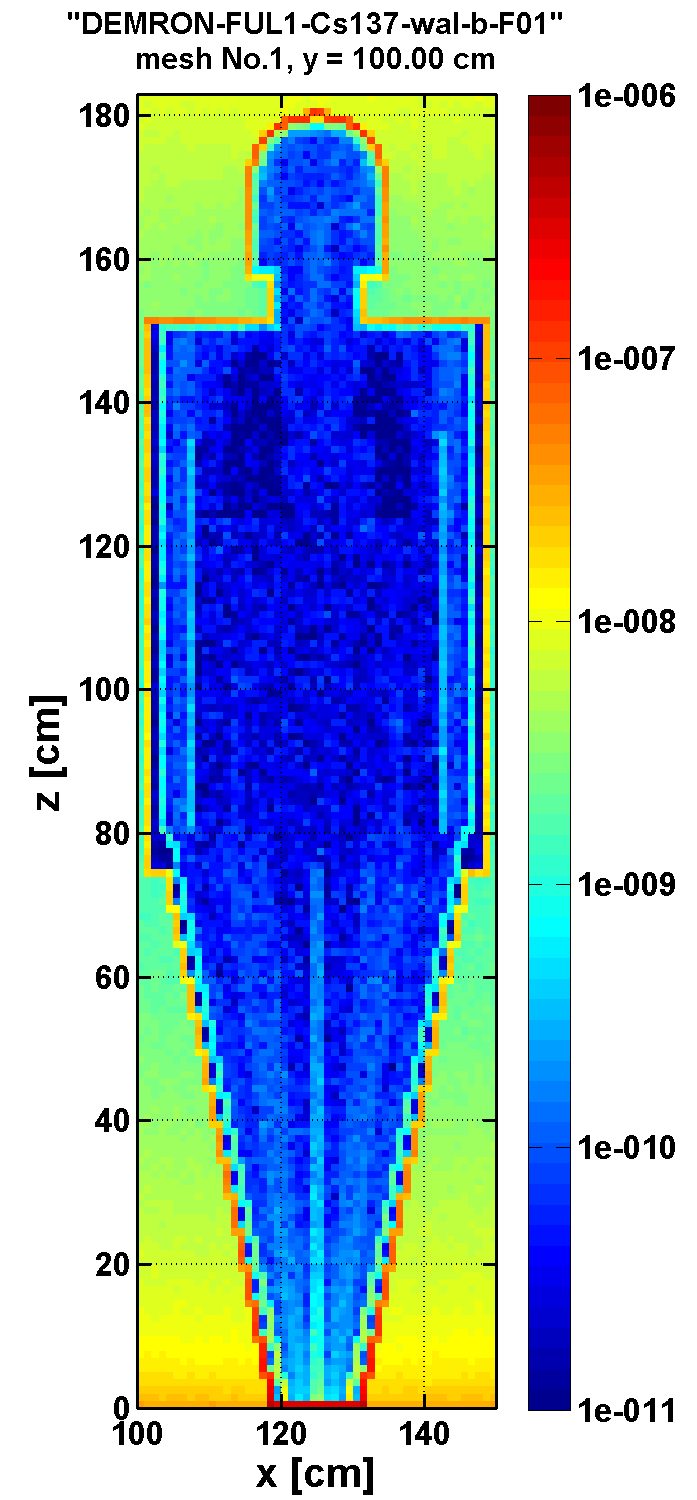

Supplement: Supplementary Materials — The electronic annex presents (1) numerical decrease of the various organs' contribution to the effective dose when protected with individual tested PPE exposed to the other simulated radionuclides dispersed in 10 m3 of the atmosphere in the RAC geometry (Table 2) and (2) visualisations of simulated ORNL phantom energy depositions while only wearing PPE preventing radioactive contamination, and the same PPE together with individual PPE protecting against X- and gamma-ray under it, in a various-dispersed radionuclide's aerosol atmosphere. [file 1641895.f1.zip › 1641895.f1/Electronic annex/Visualization of 2D distributions/Beta contribution/XZ/XZ-b-wal-Cs137-DEMRON-FUL1.png]
